# Monsoon forced evolution of savanna and the spread of agro-pastoralism in peninsular India

**DOI:** 10.1038/s41598-021-88550-8

**Published:** 2021-04-27

**Authors:** Nils Riedel, Dorian Q. Fuller, Norbert Marwan, Constantin Poretschkin, Nathani Basavaiah, Philip Menzel, Jayashree Ratnam, Sushma Prasad, Dirk Sachse, Mahesh Sankaran, Saswati Sarkar, Martina Stebich

**Affiliations:** 1 Research Station of Quaternary Palaeontology, Senckenberg Research Institute, Am Jakobskirchhof 4, 99423 Weimar, Germany; 2 Institute of Archaeology, University College London, 31-34 Gordon Square, London, WC1H 0PY UK; 3 Potsdam Institute of Climate Impact Research, Telegrafenberg A56, 14412 Potsdam, Germany; 4 Nees Institute for Biodiversity of Plants, University of Bonn, Meckenheimer Allee 170, 53115 Bonn, Germany; 5 Indian Institute of Geomagnetism, Nanabhai Moos Marg, Navy Nagar, Colaba, Mumbai, Maharashtra 400005 India; 6 Centre for Marine and Atmospheric Sciences, University of Hamburg, Bundesstraße 53, 20146 Hamburg, Germany; 7 National Centre for Biological Sciences, Tata Institute of Fundamental Research, GKVK Campus, Bellary Road, Canara Bank Layout, Rajiv Gandhi Nagar, Kodigehalli, Bengaluru, Karnataka 560065 India; 8 Institute of Earth and Environmental Sciences, University of Potsdam, Karl-Liebknecht-Straße 24/25, 14476 Potsdam, Germany

**Keywords:** Biogeochemistry, Ecology, Biogeochemistry, Biogeography, Climate-change ecology, Fire ecology, Forest ecology, Grassland ecology, Palaeoecology, Stable isotope analysis, Ecology, Biogeochemistry, Climate-change ecology, Fire ecology, Forest ecology, Grassland ecology, Palaeoecology, Stable isotope analysis, Tropical ecology, Climate sciences, Biogeochemistry, Palaeoclimate

## Abstract

An unresolved issue in the vegetation ecology of the Indian subcontinent is whether its savannas, characterized by relatively open formations of deciduous trees in C_4_-grass dominated understories, are natural or anthropogenic. Historically, these ecosystems have widely been regarded as anthropogenic-derived, degraded descendants of deciduous forests. Despite recent work showing that modern savannas in the subcontinent fall within established bioclimatic envelopes of extant savannas elsewhere, the debate persists, at least in part because the regions where savannas occur also have a long history of human presence and habitat modification. Here we show for the first time, using multiple proxies for vegetation, climate and disturbances from high-resolution, well-dated lake sediments from Lonar Crater in peninsular India, that neither anthropogenic impact nor fire regime shifts, but monsoon weakening during the past ~ 6.0 kyr cal. BP, drove the expansion of savanna at the expense of forests in peninsular India. Our results provide unambiguous evidence for a climate-induced origin and spread of the modern savannas of peninsular India at around the mid-Holocene. We further propose that this savannization preceded and drove the introduction of agriculture and development of sedentism in this region, rather than vice-versa as has often been assumed.

## Introduction

### The savannas of peninsular India: natural or anthropogenic?

Large tracts of peninsular India are characterized by savanna vegetation, with a continuous ground-layer, predominantly of C_4_-grasses and a woody layer of broadleaf or fine-leafed deciduous C_3_-trees^1,2^. Although evidence for C_4_-vegetation in India, possibly grasslands, dates back to the late Miocene^3^, two lines of evidence suggest that the today existing savannas of the subcontinent may be much younger: first, the savannas of India feature only very few tree species solely restricted to the savannas as may be expected for very old ecosystems; second, many tree species of the moist and wet forests show a clearly disjunct distribution between the moist Western Ghats of southern India and the Himalayas of North and East India, separated by extensive tracts of dry savanna vegetation^4,5^. At present even though recent work establishes that modern savannas in India fall within the climatic envelopes for natural savannas elsewhere in the world^6^, it remains unresolved if the modern savannas of peninsular India are man-made, anthropogenic derivates of deciduous forests. Further, the relative extent to which their evolution has been influenced by Holocene climate fluctuations versus the emergence of agro-pastoralism and fire activity in this region remains debated. This debate has culminated in two contrasting hypotheses about the origin of these savannas: (1) anthropogenic activities such as livestock-grazing, forest clearing and burning since the Neolithic have led to a gradual degradation of what were deciduous forests to the savannas we see today^2,7,8^; or, (2) alterations in the monsoonal moisture supply drove the establishment of savanna vegetation and in turn compelled some hunter-gatherer communities to introduce agriculture to cope with diminishing food resources^9^ while also creating attractive habitat for immigrant pastoralists^10^. However, there is no complete, paleoenvironmental dataset that has clearly differentiated between climate and anthropogenic drivers.

At continental scales, moisture availability is the primary factor controlling the distribution of savannas relative to tropical forests^11^. However, analyses of tree cover in forests and savannas of sub-Saharan Africa suggests only partially linear relationships between tree- and grass-cover relative to moisture availability, because as trees close their canopies, they shade out C_4_-grasses, while productive C_4_-grasses increase fire activity, which reduces tree cover^12,13^. Under certain climate and fire regimes, forests and savannas can therefore form alternative stable states of vegetation^14,15^. Indeed, this is the case for vast regions of the mesic tropics, including peninsular India, such that forests and savannas may occur together in the same landscape, further contributing to the interpretation of savannas as degraded forests^6^.

Globally, a mid-Holocene weakening of tropical circulation systems has been shown to be responsible for significant changes in the distribution of terrestrial biomes across the tropics, with a decline in forest extent relative to savannas, and the spread of deserts at the lower hygric limits of savanna^16–18^. Moreover, these climate and vegetation changes are considered to be the driving factors for the cultural evolution of early societies in Africa^19^. In South Asia also, a weakening in the Indian Summer Monsoon (ISM) becomes obvious from about 6.0 kyr BP, in response to decreasing solar insolation^20,21^ and has been associated with extension of farming settlements from the Indus valley towards the Ganges plains^22^.

In peninsular India potential anthropogenic drivers could include anthropogenic burning by hunter-gatherers or the clearance of forest for the establishment of agricultural fields and settlements. Archaeological research has established that the establishment of the first farming villages in the Northern Deccan (western Maharashtra, and Madhya Pradesh) took place around 4.5 kyr BP, while further to the northwest in Rajasthan and the Saurashtra Peninsula this could have begun from 5.5 kyr BP^23,24^. Farming villages did not appear on the Narmada river, however, until 4.0 kyr BP and later. Further south along the Tungabhadra and the region where it meets the Krishna river, the first food production systems were focused on livestock pastoralism and seasonal ashmound sites with sedentary villages appearing 4.2–4.0 kyr BP^24–26^. On this basis we expect anthropogenic savannas to emerge 4.5–4.0 kyr BP. Prior to these periods the region was occupied by bands of hunter-gatherers. While it is plausible that hunters may maintain more open vegetation through burning, there is no evidence that such practices were not already well-established in Late Pleistocene or Early Holocene times. Indeed, where micro-charcoal records are available in India they show marked declines in evidence for burning before the advent of farming, such as ca. 6.5 kyr BP in Rajasthan (Thar Desert) at Lunkaransar^27–29^. In the Thar Desert and in the Ganges plains the highest micro-charcoal levels predate 8.0 kyr BP, associated periods of microlith-using hunter-gatherer sites in both regions^28–30^. The predominance of microliths occurs on sites in the Deccan from ~ 30,000 BP up to the Neolithic^31^, and therefore provides no indication for a shift in hunter-gatherer culture that might correlate with anthropogenic creation of savannas.

**Figure 1:**
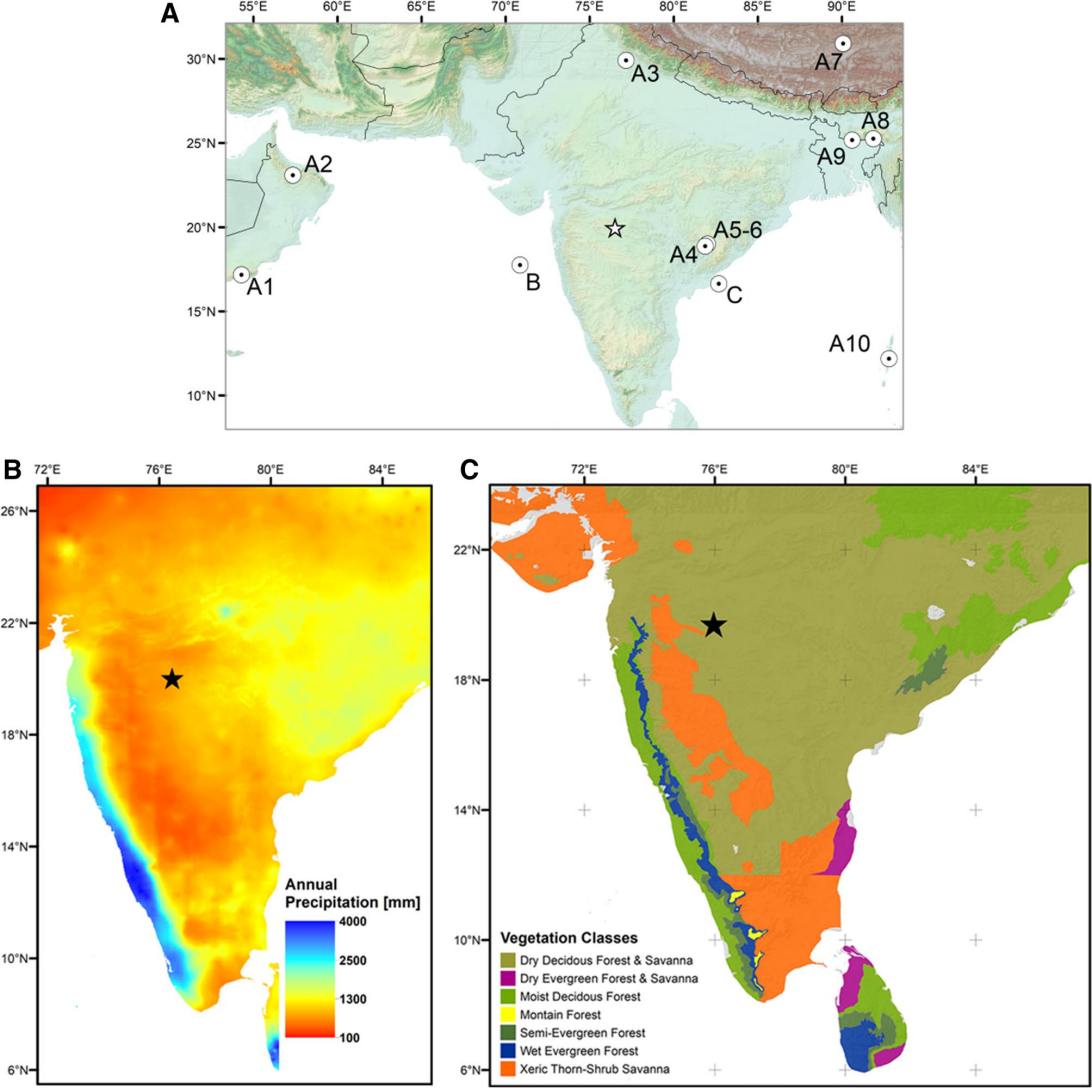
(**A**) Map of South Asia and the location of climate and environmental archives shown in Figs. 2 and 3. A1-10: speleothem records used to construct Fig. 2: B^36–40^. B: marine core SK-148/55 from^41^ (Fig. 2: C). C: marine core NGHP-16A^42^ (Fig. 3: B); Asterisk: location of the Lonar Crater Lake. (**B**) Mean annual precipitation (MAP) based on^43^, and (**C**) potential natural tropical vegetation of peninsular India modified from^44^ and geo-referentiated using ArcGIS 10.7.1 (ESRI, 2019)^45^.

A direct linkage between the evolution of the savannas of peninsular India and Holocene climate change, fire and the emergence of agriculture on regional to subcontinental scale still remains unproven, due to the lack of high resolution terrestrial environmental data. To decipher these linkages we investigated a Holocene lacustrine sedimentary sequence from the Lonar Meteorite Crater Lake (19.98 N; 76.51 E, Fig. 1A), located in the semi-arid savanna zone of Peninsular India^32^. Changes in tree flora and fire activity were reconstructed using pollen analysis and sediment micro-charcoal counts, respectively. To identify changes in the contribution of terrestrial C_3_- and C_4_-plants (i. e. forest vs. savanna type vegetation), we used a record of leaf wax n-alkane δ^13^C-values^33^. Finally, we correlated our results with available climate reconstructions and the spatial distribution of more than 800 independently-dated Neolithic archaeological records (SI: Tab. 2) from peninsular India to decouple the relative influences of Holocene climate change and anthropogenic activities on the establishment of savanna vegetation in the region. Our study is of significant interest for the future management of India’s savannas and tropical forest ecosystems, as savannas in this region are expected to be replaced by moist forests under different climate change scenarios, which predict between 15 and 40% increase in mean annual precipitation (MAP) in peninsular India until the end of the twenty-first century^34,35^.

### Vegetation in peninsular India and Holocene fluctuations of the Indian Summer Monsoon

The climate of India is primarily controlled by the ISM which is a manifestation of the pole-ward migration of the Intertropical Convergence Zone (ITCZ) on an intra-annual timescale^46^. High insolation over India and the Tibetan Plateau during north-hemispheric summer leads to a strong atmospheric pressure gradient between the Asian landmass and the Indian Ocean, which drives moisture-laden winds towards the subcontinent^46,47^. Effective rainfall in peninsular India is thus widely restricted from June to September, while the remaining part of the year receives little to no rainfall.

Due to the orographic setting of peninsular India, a gradient in mean annual precipitation (MAP) and vegetation cover exists from the SW to NE (Fig. 1B, C). Tropical evergreen and semi-evergreen forests are restricted to the SW of the Western Ghats that receive MAP > 2000 mm, while interior peninsular India is covered with savanna vegetation. Based on the woody components, these savanna formations can be distinguished into deciduous broadleaf savannas under moister conditions, with MAP between 650 and 1800 mm, and xeric fine-leaved and thorn savannas that receive MAP < 650 mm^44^ (Fig. 1C). On the northern part of the Deccan characteristic vegetation formations of the broadleaf savannas mainly comprise the *Acacia-Anogeissus*-series (on very dry sites), *Terminalia*-*Anogeissus-Tectona*-series (on dry sites), the *Tectona-Terminalia*-Series and the *Tectona-Terminalia-Adina-Anogeissus*-series (on intermediate sites), and the *Tectona-Dillenia-Lagerstroemia-Terminalia*-series (on moist sites^8,28^). The *Acacia-Capparis*-series is the predominant vegetation-series of the xeric savanna types on the Deccan^8,28^. At the upper moisture limits of the savannas, moist deciduous forests of the *Tectona*–*Dillenia*–*Lagerstroemia*–*Terminalia*-series occur, which consist of deciduous as well as semi-evergreen and evergreen tree species^8^. C_4_-species comprise the grass layer in the deciduous and xeric savanna types, while C_3_-grasses, especially bamboos, are common constituents of the moist deciduous, semi-evergreen and evergreen forests^4,48^.

**Figure 2:**
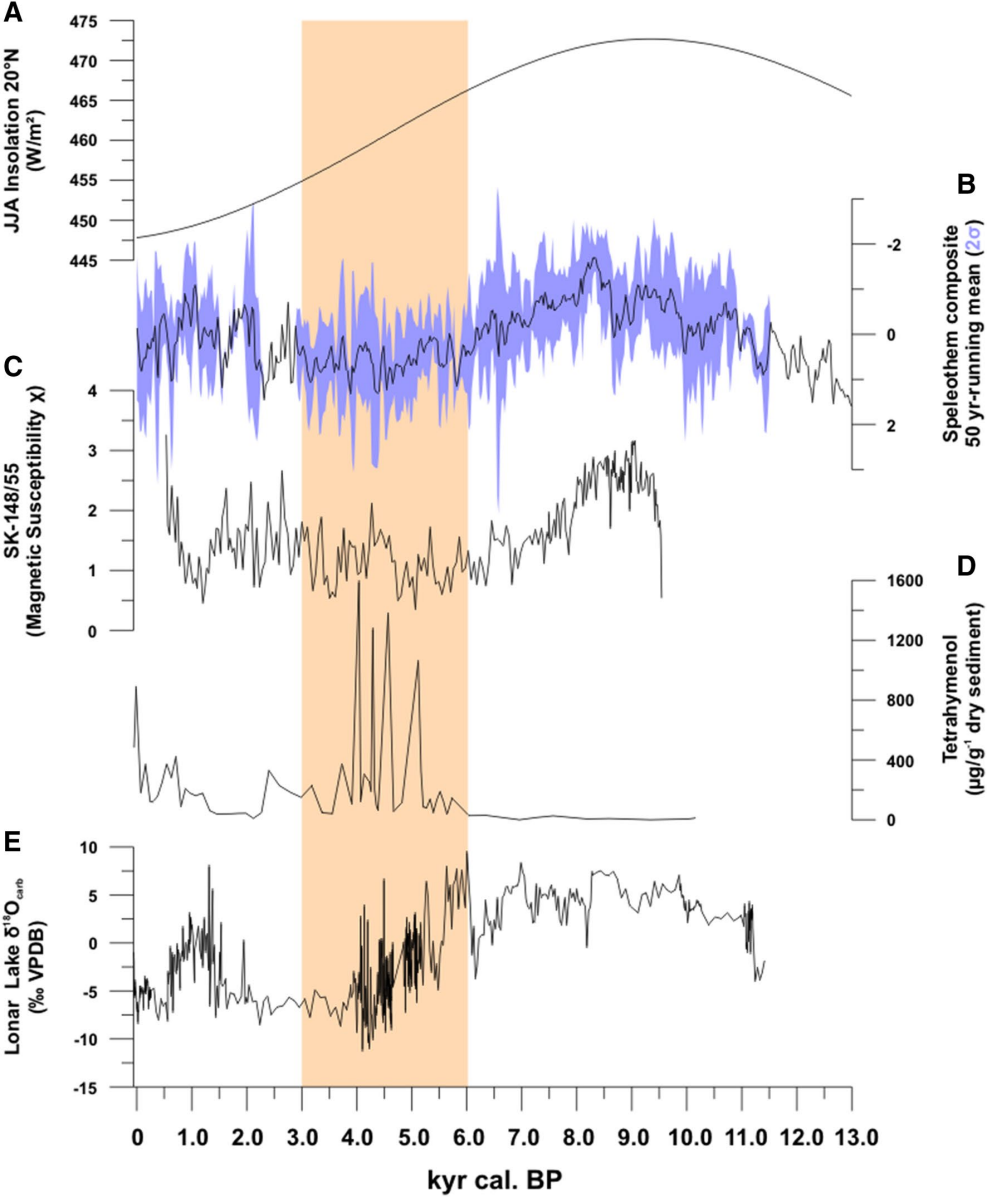
Phases of ISM-activity since the Late Glacial. (**A**) Holocene solar insolation at 20° N based on^54^; (**B**) speleothem composite 50-years running mean (2σ) as proxies for ISM rainfall based on^36–40^ (SI: Fig. 3); (**C**) magnetic susceptibility of marine sediment core SK-148/55^41^ as proxy for freshwater influx; and Lonar Lake sediment core L-23, with (**D**) tetrahyamenol flux as proxy for past lake water salinity and (**E**) oxygen isotope ratios of lake sediment carbonates as proxy for past lake levels. Sand-colored box: onset of Holocene ISM weakening following^52^.

In recent decades, a growing number of high resolution terrestrial and marine Late Glacial and Holocene climate proxy records have been established throughout the ISM-realm. Within the ISM domain, the early to mid-Holocene is the wettest phase of the entire Holocene^49^. Arabian Sea proxy records document increased freshwater input from 9.0 until at least 7.5 kyr cal. BP^41^. The δ^18^O-values in speleothems from NE India^36^ and Oman^50^ reflect highest rainfall between ca. 11.0 and 6.0 kyr cal BP. These reconstructions are in agreement with the wettest conditions in Lonar between 11.0 and 6.3 kyr cal. BP (Fig. 2). Geomorphological evidence^51^ for higher precipitation and river runoff on the Deccan Plateau is also seen in increased fluvial activity between 9.0 and 5.0 kyr BP. Throughout South Asia, ISM weakening is apparent at the beginning of the Mid-Holocene in response to decreasing north-hemispheric insolation. Increasing δ^18^O-values in speleothems point to moisture reduction in both NE India and S-Arabia between 6.3 and 5.8 kyr cal. BP^36,37^. Multiproxy data from Lonar sediments indicate the start of this drying trend at ca. 6.2 kyr cal. BP^52^ with the weakening ISM culminating in two multi-centennial droughts between 4.7 and 3.5 kyr cal. BP and 2.0–0.6 kyr cal. BP. These abrupt droughts, linked to the El Niño-Southern Oscillation (ENSO) and shifts in the position of the Indo Pacific Warm Pool^52^, have also been documented in terrestrial ISM proxy records from India and Arabia^36,38,52,53^.

## Results

### Holocene vegetation shifts and the evolution of Savanna after 5.0 kyr cal. BP

**Figure 3:**
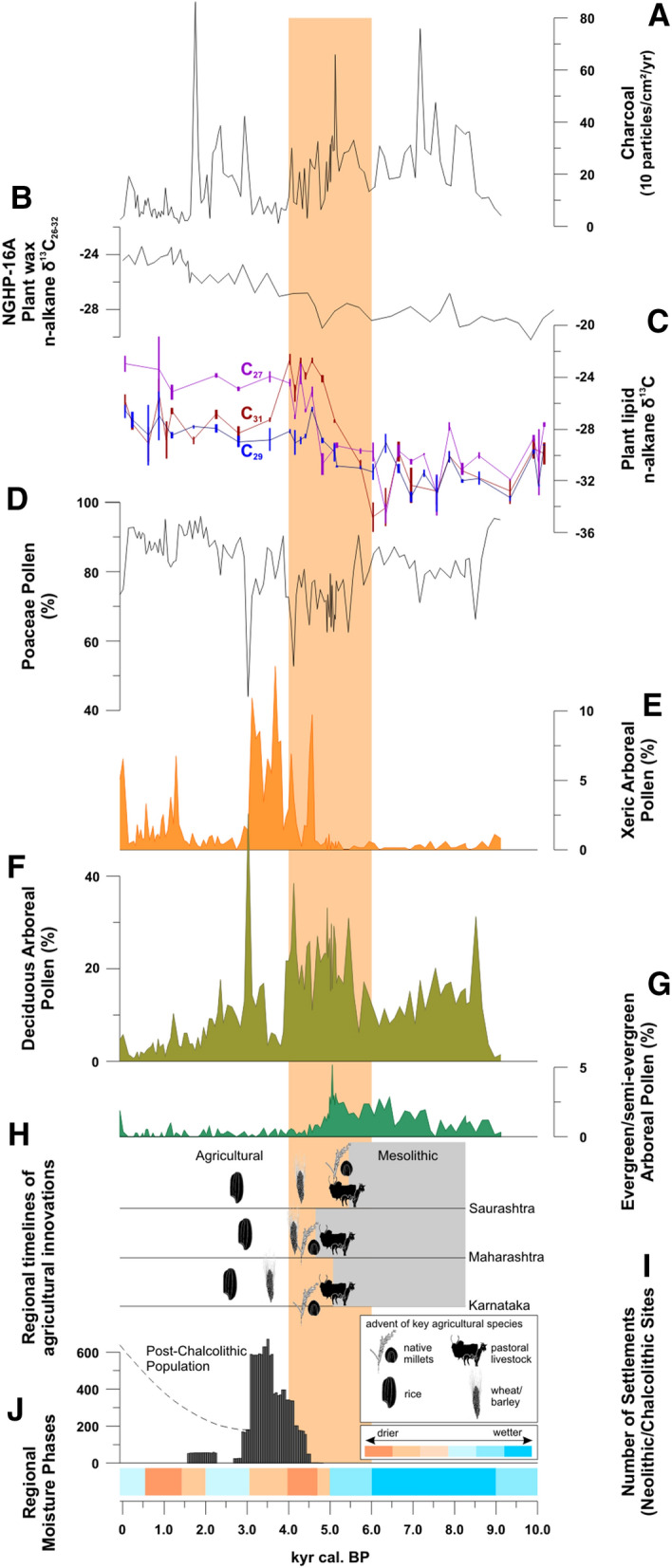
Holocene vegetation development and fire activity derived from Lonar Lake sediment core L-23: (**A**) Micro-charcoal influx as indicator of fire activity; (**C**) N-alkane δ^13^C as proxy for photosynthesis types of terrestrial plants, (**D**–**G**) proportions of terrestrial plant pollen types, indicative for past vegetation (SI: Tab. 1, SI: Fig. 2). (**B**) Leaf wax N-alkane δ^13^C as proxy for photosynthesis types of terrestrial plants from marine core NGHP-16A^42^. (**H**) Regional timelines of agricultural innovations from^57^, and (**I**) number of Neolithic/Chalcolithic archaeological sites in South India (based on SI: Tab. 2). (**J**) Regional moisture phases reconstructed from the Lonar pollen assemblage and the geochemistry of core L-23^52^. Sand-colored box: onset of Holocene ISM weakening based on^52^. Figures in (**H**) drawn by D. F.

**Figure 4:**
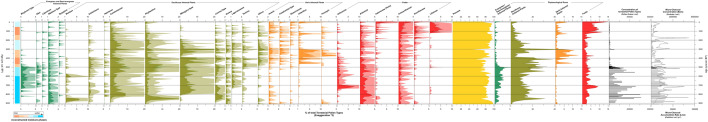
Simplified pollen and micro-charcoal diagram of Lonar Lake L-23 composite sediment core. Attribution of pollen types to vegetation classes follows Tab. 1, SI. Regional moisture phases reconstructed from the pollen assemblage and geochemistry of core L-23^52^. The phytoecological pollen sums consist of: evergreen/semi-evergreen pollen types: *Maytenus, Mallotus, Ligustrum, Olea, Syzygium*. Deciduous pollen types: Annona*, Bombax, Citrus*, Combretaceae, Cordia*, Dendrophthoe*, Dodonea*, Grewia, Helicteres*, Lagerstroemia, Lannea, Madhuca*, Mitragyna, Morinda*, Phyllanthus, Radermachera*, Schleichera, Spondias*, Tectona, Woodfordia*, Wrightia*. Xeric pollen types: Acacia, Ailanthus, Azadirachta, Bauhinia*, Cassia*, Erythrina*, Mimosa*, Paracalyx*, Prosopis, Rhamnaceae, Xeromphis*. Forb pollen types: Amaranthaceae, Asteraceae: Asteraceae indet, *Xanthium, Echinops*; *Artemisia*, Caryophyllaceae*, Fabaceae indet*, Tiliaceae indet*, Corchorus*, Triumfetta*, Euphorbiaceae*, Chrozophora*, Ricinus*, *Solanum*,* Aspidopterys*, Boerhavia*, Calligonum*, Enicostema*, Cucurbitaceae indet*, Lagenaria*, Luffa*, Cadaba*, Tinospora*, Pedalium*, Neanotis*, Cleome*, Anacardiaceae indet*, Acanthaceae: Acanthaceae indet*, Adhatoda*, Asystasia*, Justicia*, Lepidaghatis*, Peristophe*, Rostellularia*, Rungia*; Boraginaceae*, Brassicaceae*, Apiaceae*, Lamiaceae*, Malvaceae*, Convolvulaceae*, Plantaginaceae*, Clematis*, Ranunculus acris*, Dipsacaceae*, Ephedra distachia*, E. fragilis*, Cistaceae*, Verbascum*, Impatiens*, Liliaceae, Urticaceae. *Pollen with < 0.5% to the terrestrial pollen count are presented only as part of the phytocecological sums.

Resulting from lower sedimentation rates^52^, pollen and charcoal-particle concentration is significantly higher in the lower sequence of core L-23, dated to between 9.1 and ca. 5.0 kyr cal. BP (Fig. 4). The lowest part of the pollen profile, dated to the early Holocene between 9.1 and 8.5 kyr cal. BP, is composed of low percentages of evergreen and semi-evergreen arboreal elements along with some xeric taxa and increasing amounts of deciduous tree pollen types, e. g. *Phyllanthus*, *Bombax* and Combretaceae (Figs. 3, 4). From 8.8 kyr cal. BP, moist deciduous vegetation was established in the Lonar region as indicated by increasing percentages of evergreen/semi-evergreen pollen types, e. g. *Maytenus*, alongside the occurrence of deciduous tree pollen types, thus reflecting significantly enhanced moisture during the early Holocene. N-alkane ^13^C-ratios between − 33 and − 31 ‰ reflect minimal contribution of C_4_-plants to the terrestrial organic matter in the Lonar Lake sediments, thus C_3_-only-vegetation persisted throughout this time period (Fig. 3). High pollen percentages of the woody evergreen and semi-evergreen elements *Maytenus*, *Olea* and *Ligustrum* continued after 6.3 kyr cal. BP, alongside increasing contributions of deciduous pollen types, e. g. *Phyllanthus*, *Mitragyna* and Combretaceae (Fig. 4), in response to reduction in ISM-derived rainfall or prolongation of the dry season^55^. However, the pollen record suggest that initially the annual rainfall amount continued to be high enough to support growth of evergreen and semi-evergreen forest elements sensitive to low rainfall conditions and more pronounced seasonality. Leaf wax δ^13^C values, begin to increase at ca. 5.9 kyr cal. BP, in particular for the *n*C_31_ alkane, which is often more prominent in grasses^56^, indicating an increase in C_4_-grass cover. From 5.7 kyr cal. BP onwards, the pollen assemblage reveals a significant change in vegetation. While proportions of deciduous pollen types further rise, most evergreen and semi-evergreen taxa disappear after 5.0 kyr cal. BP, reflecting the overall reduction of MAP, but also suggesting prolonged dry seasons. Until 4.7 kyr cal. BP leaf wax δ^13^C values for all long-chain n-alkanes shifted to values between -23‰ (for *n*C_31_) and -28‰ (*n*C_29_) (weighted average of ca. -27‰) indicative of mixed C_3_-C_4_-vegetation^56^, thus suggesting a displacement of C_3_ elements by C_4_-grasses and the formation of savanna. By 4.7 kyr cal. BP pollen percentages of xeric elements, e. g. *Acacia*, *Ailanthus,* and later *Prosopis* increase noticeably, in line with the rapid shift to dry conditions^52^. However, until 3.8 kyr cal. BP, and again after 3.4 kyr cal. BP, significant abundance of deciduous arboreal pollen and the variability of δ^13^C values among the different leaf wax n-alkane homologues suggest that the savannas likely featured a relatively dense woody cover. While deciduous tree pollen types steadily decrease from 2.5 kyr cal. BP, proportions of xeric elements rise after 1.3 kyr cal. BP. Subsequently, after 1.2 kyr cal. BP, a rapid increase in forb pollen types, e.g. members of Asteraceae, Acanthaceae and Liliaceae (Fig. 4), which also comprise species indicative for grazing pressure^48^, is interpreted as the first direct evidence for anthropogenic impact on the vegetation at Lonar.

### Fluctuations in Holocene fire activity

Between 8.5 and 4.5 kyr cal. BP micro-charcoal fluxes in Lonar Lake sediments, a proxy for past fire occurrence, show high but rather constant fire activity (Figs. 3, 4). By 3.9 kyr cal. BP a rapid loss in deciduous arboreal vegetation can be inferred from the pollen spectra, confirming a further reduction of MAP. From 4.4 to 3.1 kyr cal. BP decreasing micro-charcoal fluxes reveal a reduction in fire activity. Following this phase, increasing values of deciduous arboreal pollen and decreasing values of xeric pollen types indicate a period of woody thickening from 3.4 until 2.0 kyr cal. BP. The leaf wax δ^13^C values provide no evidence for a re-appearence of the initial C_3_-only-vegetation; thus savanna vegetation prevailed throughout this interval of somewhat higher ISM activity. From 3.0 until 1.8 kyr cal. BP, higher fire activity can again be deduced from the micro-charcoal fluxes. The second phase of prolonged aridity between 2.0 and 0.6 kyr cal. BP, as deduced from evaporitic and isotopic proxies in Lonar sediments^31^, corresponds to a decline in deciduous tree pollen percentages. From 1.7 kyr cal. BP till modern times, fire activity was relatively low as inferred from low micro-charcoal fluxes to the Lonar Lake sediments.

## Discussion

### Mid-Holocene savannization of peninsular India: the roles of climate and fire

Our new data suggest that in contrast to the semi-arid vegetation in modern times, moist deciduous forests with significant proportion of tropical evergreen and semi-evergreen elements covered the central Deccan during the early to early mid-Holocene. This is in line with paleo-botanical data from other parts of India, which suggest moister-than-present vegetation between 9.0 and 5.6 kyr cal. BP^58,59^. Subsequently, savanna formation occurred after 5.0 kyr cal. BP in the Deccan, with the step-wise reduction in ISM activity and associated MAP leading to a gradual spread of deciduous trees and the widespread expansion of C_4_-grasses, which represent functional savanna vegetation.

The global distribution of savannas relative to forests is controlled primarily by moisture availability, including effective rainfall and rainfall seasonality^11,60^. A review of the modern distribution of savannas in India suggests an upper hygric limit of ~ 1750 mm MAP^6^, which is close to the lower hygric limit for the occurrence of tropical wet evergreen and semi-evergreen tree species in the woodlands of South India (1800 mm MAP)^44^. Since the modern distribution of trees species relative to the climate in India is relatively well known^8,44^, the occurrence of certain tree pollen types in the Lonar pollen record may thus serve as a robust indicative tool to delineate MAP during the Holocene. Based on our results, the appearance of the evergreen tree pollen types *Olea* and *Ligustrum* at Lonar between 7.2 and 5.0 kyr cal. BP (Fig. 4) indicate a minimum MAP of ca. 1800 mm during the early to early mid-Holocene^44^. The subsequent disappearance of these pollen types at 5.0 kyr cal. BP suggests a drop of MAP below the critical rainfall value corresponding to the wet end of savanna vegetation in India^6,11,44^. Since these pollen types do not reappear after 5.0 kyr cal. BP, our data reveal that climate conditions favoured savanna vegetation through the mid- and late Holocene. Moreover, stable carbon isotope data recovered offshore the Godavari-Krishna river sediment fan at the Bay of Bengal, also reveals a spread of C_4_-vegetation from 6 kyr BP onwards (Fig. 3B)^42^. Those results clearly reflect that the process of savannization was not solely restricted to the Lonar Lake region, but occurred in large parts of the Indian peninsular.

After moisture availability, fire is a key factor maintaining savanna vegetation in regions that might otherwise be wet enough to support forests^15^. Studies from Africa, Australia and SouthAmerica reveal that fire intensity is generally higher in moist savannas compared to tropical forests, because of the large amounts of flammable biomass provided by fire-adapted C_4_-grasses^15^. Moreover, since amounts of grass biomass and the fire regime are positively related, fire intensity tends to be higher in moist savannas compared to dry savanna types^11,61^. In contrast, not only peninsular India’s woody deciduous savannas, but also its moist deciduous forests feature 4 to 10-times higher fuel loads, compared to open savanna vegetation^62^. In the moist deciduous forests, the leaf litter shed during the dry season serves as the primary fuel for frequent forest fires^62^. Consequently most deciduous, but also some evergreen forest trees, e.g. *Olea* and *Syzygium*, feature good morphological adaptations to fire damage^63^. Between 8.8 and 5.0 kyr cal. BP deciduous tree cover was relatively dense as revealed by the pollen assemblage. Thus, a high flux of dry leaf biomass can be expected, leading to recurrent fire events. The high fire activity during the early and early mid-Holocene is furthermore consistent with the hypothesis that Indian Mesolithic hunter-gatherers used fire to manage landscapes for foraging and hunting^9,28^. We cannot exclude, that the occurrence of fire, at least in parts, can also be attributed to Mesolithic hunter-gatherers, but in the absence of any other comparable paleo-record from South India this hypothesis can yet not be tested.

However, as evident from the persistent high fluxes of charcoal particles in conjunction with the fossil pollen assemblage, our data gives no indication that the fire regime per se led to significant changes in the vegetation composition during the early to mid-Holocene period. Subsequently from ~ 4.5 kyr cal. BP, under reduced MAP, increasing fractions of xeric arboreal and grass pollen, and decreasing percentages of deciduous pollen types indicate opening of the tree cover. We suggest that this caused a change in the fuel loads to grass litter, and a shift in the fire regime, particularly after 4.0 kyr cal. BP. However, between 3.3 and 2.0 kyr cal. BP, increasing values of deciduous tree pollen and a rise in charcoal fluxes suggest that leaf litter was likely to have been an important component of the fuel load during this period of high fire activity.

While several authors have argued that fire and fire-related vegetation changes in tropical Asia are primarily caused by human activity^64^, there is no evidence from peninsular India for noticeable anthropogenic influence on the landscape before the onset of the Neolithic/Chalcolithic period dated to ca. 4.5 kyr cal. BP in the northwestern Deccan and 4.0 kyr cal. BP more widely (Figs. 3, 4), and associated with the emergence of agricultural villages^10,23,24^. The main influences during this period are likely to have been localized clearance for agriculture and grazing pressure rather than large scale burning.

### Savanna formation and cultural evolution in peninsular India since the mid-Holocene

Our results suggest that early agriculture played no active role in the initial establishment of savanna vegetation in South India because our savanna signal begins at least 500 to 1000 years earlier than the archaeological Neolithic. In contrast, the role of large scale climate and vegetation changes on the evolution of agriculture and sedentism in South India remains unclear till date. The Deccan is one of the centers of neolithisation in South Asia^10^. The shift to agriculture and later sedentism occurred largely independently from other core areas of early agriculture that were located on the Indo-Gangetic plains^10,24^. Existing models suggest pastoralists with domesticated fauna were first established east of the Thar Desert ~ 5.5 kyr cal BP and spread with their livestock throughout the central Indian peninsula ~ 5.0 kyr cal. BP, reaching southern Karnataka ca. 4.5 kyr cal BP^10,24^. Domestication of crops native to the landscapes of Peninsular India began between 5.0 and 4.0 kyr cal. BP^10,25,65^, either by local hunter-gatherer groups or immigrant pastoralists or mixed communities. Most likely the earliest cultivation involved regular multi-annual mobility, e. g. slash-and-burn cultivation^9^, with long-term sedentary farming emerging 4.2–3.9 kyr cal. BP^25–27^. Our new data provides detailed insights into the impact of mid-Holocene climate change on the previously proposed scenarios^24,42^ for the emergence of sedentary farming in Peninsular India. We suggest that the climate induced large scale changes in vegetation after 5.0 kyr cal. BP reduced the availability of forest derived wild foods, while increasing attractive grazing environments. This promoted (1) the spread of pastoral adaptations, which were established east of the Thar Desert ca. 5.5 kyr cal. BP and in the Deccan 5–4.5 kyr cal. BP; and (2) experimentation with the management and cultivation of reliable plant foods native to the woodland-savanna margins, which formed the basis for staple crops in the peninsular Neolithic^24^. Subsequent weakening of the ISM promoted the establishment of widespread farming and sedentism by 4.0 kyr cal. BP.

**Figure 5:**
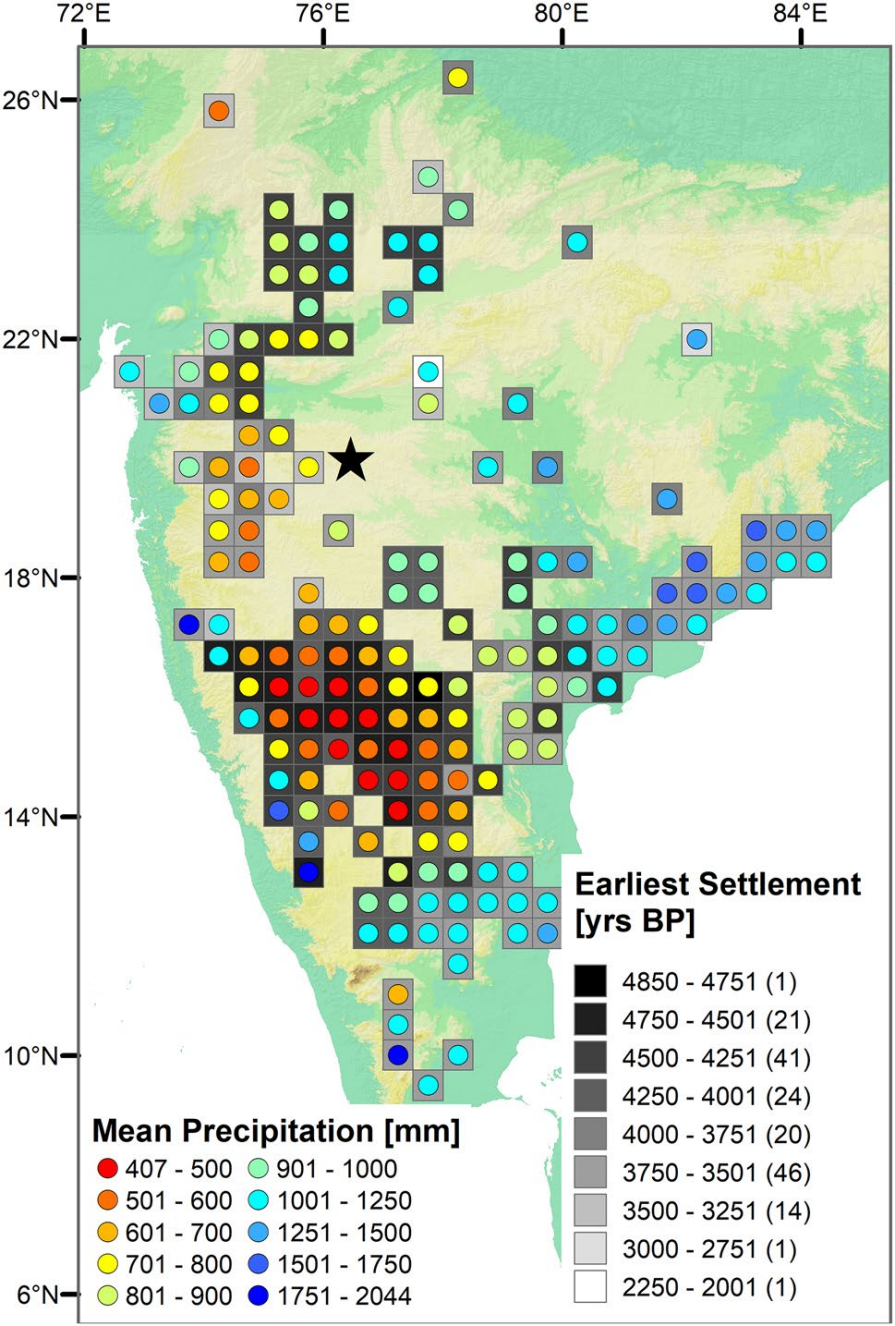
Distribution and first appearance of Neolithic/Chalcolithic settlements in peninsular India, along with modern patterns of MAP (Based on SI: Tab. 2). Map made with ArcGIS 10.7.1 (ESRI, 2019)^45^.

The modern distribution of the wild progenitors of crops domesticated within the Indian Neolithic reveals that they originated from two contrasting habitats: (1) moist deciduous forest formations of the Indian peninsula, which serves as setting for wild *Vigna* pulses and some tubers; and (2) savanna which is the habitat for indigenous millets and the legume *Macrotyloma uniflorum*^10,28,65^. Our data shows that moist deciduous forests had a much wider distribution throughout the peninsula during the early Holocene. Therefore, they were both food-sources for hunter-gatherers and habitat for the progenitors of different cultivated crops. When forest structure gradually began to change between 6.3 and 5.0 kyr cal. BP, hunter-gatherer communities would have lost some wild food resources, necessitating the cultivation of crops to maintain supplies of these species. Today, the most arid areas in peninsular India are in the lee of the Western Ghats, and would have been the first to feature savanna vegetation. In these proposed core areas of savannization, cattle pastoralism was established by ca. 5.0 kyr cal. BP^10,25^, together with slash-and-burn cultivation^66^. With the weakening of ISM after 5.0 kyr cal. BP, the resulting spread of savanna coupled with growing human population led to original slash-and-burn cultivation giving way to larger investments in field maintenance and increasing settlement permanence. It is notable that after 4.5 kyr cal. BP, permanent settlements first emerged in the Deccan focused on two parts of the suggested core area of savannization: (1) on the leeward side of the Western Ghats along the Narmada and Tapti river systems; and (2) on the granite peneplains along the middle of the Tungabhadra and Krishna rivers (Fig. 5; SI: Tab. 2). In the village-based Neolithic, more intensive strategies developed, including two seasons of cultivation^24,67^. This served to spread risks from high seasonality and inter-annual rainfall variability^66^, and may have involved some small-scale irrigation of winter crops^24,67^. Subsequently, after 4.0 kyr cal. BP, agricultural villages became widespread throughout the expanding savannas of the Deccan^68^ (Fig. 5). Interestingly, declines in these village societies, including widespread village abandonment took place at 3.3–3.2 kyr cal. BP^68,69^, coincident with the Lonar evidence for increased woody cover and burning, suggesting that environmental and subsistence changes may also be linked in this period.

## Conclusion

In conclusion, ISM weakening was the most significant factor in the reduction of forests and expansion of savanna vegetation in peninsular India during the mid-Holocene. Our results show that by the early Holocene, deciduous forests were prevalent across peninsular India and persisted through to the mid-Holocene. The weakening of the ISM that began in the mid-Holocene heralded the assembly and spread of the modern day savannas of peninsular India. We hypothesize that this savannization created attractive habitat for pastoralist immigrants, affected the food supply of hunter-gatherer communities and eventually triggered the transition to agriculture on the Deccan. We do not doubt that since the mid-Holocene, steadily increasing human impacts on the landscape have further altered the vegetation and helped to maintain open savannas in peninsular India. However, our data point unambiguously to a climate-driven origin of these savannas.

## Material and methods

### Site characteristics and chronology

The studied sediment record was retrieved from mid-Pleistocene-aged, highly alkaline Lonar Meteorite Crater Lake (19.98 N; 76.51 E) located on the basaltic Deccan Plateau of Maharashtra, India. The climate is semi-arid, with MAP of ~ 800 mm^32^. About 85% of MAP is derived from ISM and restricted to the summer month from mid-June to mid-September. Dry season lasts 8 month^32^. Lonar Lake is a terminal lake, fed by two perennial and ~ 15 seasonal tributaries. The area outside Lonar Crater is used for agriculture and livestock grazing, featuring thorn-shrub-like browsing resistant xeric woody plant species. The inner crater largely houses tree-savanna with a woody stratum composed of deciduous broadleaf elements and a grass layer exclusively build up from C_4_-grasses^32^. Under modern climate conditions the Lonar Crater is located close to the lower hygric end of the teak bearing mixed dry deciduous vegetation series^44^. Based on the climatic and vegetational features, we suggest that the Lonar Crater might be representative for the environmental history of the semi-arid south India. The age-depth model of the studied 10 m-long sediment profile is based on 23 AMS-^14^C dates from terrestrial plant macros and Gaylussite crystals^52^ (SI: Fig. 1). For the specific interpretation of the Lonar Lake carbonate ^18^O-record please refer to the supplementary information (SI).

### Pollen and charcoal analysis

The sediment core was continuously sampled in 1 cm intervals. Depending on the sedimentation rate, subsamples from the sediment core LON-23 were palynologically analyzed in 1–10 cm intervals, providing a temporal resolution of 150 years between 9.1 and 5.0 ka cal. BP and ca. 20–40 years from 5.0 ka cal. BP onwards. Sediment sample preparation for pollen analyses followed standard procedures using KOH, HCl, hot HF and acetolysis-mixture. All samples were sieved over 200 µm and ultrasonic sieved using 5 mesh gauze. Identification of pollen types is based on the pollen collection for South Asia at Senckenberg Research Station, Weimar (Germany) and pollen keys covering tropical Asia^70,71^. At least 600 terrestrial pollen types were counted per sample. Classification of the vegetation types followed the work of^8,44^. Based on these classifications, arboreal pollen types identified in the Lonar sediment record were attributed to three main hygric forest types (SI: Tab. 1). To test whether the arboreal pollen assemblages correspond to those modern forest types Principal Component Analysis (PCA) was applied (SI: Fig. 2). The results demonstrate an overall good correspondence of the fossil tree pollen spectra with the modern hygric forest types. A detailed pollen diagram is shown in Fig. 4.

Counting of micro-charcoal particles was undertaken along with pollen analysis following the guidelines of^72,73^. A minimum of 200 totally black, rectangular and opaque particles with a diameter of more than 10 µm were counted per sample. The results are presented as micro-charcoal accumulation rates (fluxes) as charcoal particles (CP) per cm^2^ per year. The fire regime is defined as the function of fire severity and fire frequency. However, the temporal resolution of the present study does not allow distinction between fire severity and fire frequency, thus changes in the charcoal fluxes can both be a result of alterations in the severity, but also in the recurrence rate of fire events^74^.

### Composite ISM-speleothem record

Multiple speleothem-based isotopic proxy records of Holocene moisture changes have been established in the ISM-realm of India during the last two decades. None of these provide a fully coverage of the time period under study because of the limited length of the individual speleothem sequence or hiatus which result from missing stalagmite growth. To cope with the fragmentary speleothem record and to rule out possible impacts of local climate and site specific factors^75,76^, as well as dating uncertainties, we constructed a stacked speleothem record, representing the south-west monsoon moisture supply since the Weichselian Late Glacial. The composite speleothem-based (Fig. 2B) record for the monsoon variability was constructed by using the z-scores of 10 proxy records from Baratang, Dandak, Hoti, Jhumar, Mawmluh, Qunf, Sahiya, Tianmen and Wahshikar cave^36–40^. Within overlapping moving windows of length 200 years (100 years overlap), for each proxy record the median of the z-score values were separately estimated and the numbers of observations (data points) of the proxy covered by this window were counted (SI: Fig. 3). Thus, for each time window one median value and the number of data points for each of the proxies are available. Next, these separate median values were weighted by the number of observations that are inside the window and these weighted median values are summed up. This provides a composite value of weighted averages for each window. The standard deviation across the weighted median values of the proxy records within one window allows for a rough evaluation of the spread of these values.

### Archaeological data

A database of reported Neolithic and Chalcolithic sites was compiled for the modern Indian states of Madhya Pradesh, Maharashtra, Karnataka, Andhra Pradesh, Telengana, and Tamil Nadu. A total of 812 Neolithic/Chalcolithic sites were analyzed for their distribution in time and space in south India. Description and age discrimination of those sites is based on an extended review of the archaeological literature (SI: Tab. 2). Since only a few sites have been absolutely dated via ^14^C-dating, the age discrimination is based on the known age of specific cultural artefacts, especially on ceramics. These chronological assignments have been drawn from primary references whenever possible, and otherwise determined by DF and judged in relation to his experience with South Indian assemblages related to the chronological frameworks outlined in^24,25,69,77^. The geolocations of sites when possible was taken from reported geo-coordinates in primary sources, if available, or determined in Google-Earth when location maps in primary reports made this possible, but when these more precise approaches proved impossible they were plotted to nearest Taluk or determined through digitization of larger distribution maps^78–80^. The distribution and the timing of the first appearance of the Neolithic/chalcolithic sites plotted against mean annual rainfall on a 50 × 50 km grid layer. We used this format to explore both sampling bias and clustering of the archaeological sites along river valleys.

## Supplementary Information

Supplementary Information.
