# Supplementary material for: Monsoon forced evolution of savanna and the spread of agro-pastoralism in peninsular India
Source: Sci Rep. 2021 Apr 27;11:9032. doi: 10.1038/s41598-021-88550-8 (PMC8079367; doi:10.1038/s41598-021-88550-8)
Supplement: Supplementary file 1 — Supplementary Information. [file 41598_2021_88550_MOESM1_ESM.docx]

**Supporting Information (SI) to:**

**Monsoon forced evolution of savanna and the spread of agro-pastoralism in peninsular India**

**Authors**

Nils Riedel*; Senckenberg Research Institute, Research Station of Quaternary Palaeontology, Am Jakobskirchhof 4, 99423 Weimar, Germany

Dorian Q. Fuller; University College London, Institute of Archaeology, 31-34 Gordon Square, London WC1H 0PY UK

Norbert Marwan; Potsdam Institute of Climate Impact Research, Telegrafenberg A56, 14412 Potsdam

Constantin Poretschkin; University of Bonn, Nees Institute for Biodiversity of Plants, Meckenheimer Allee 170, 53115 Bonn, Germany

Nathani Basavaiah; Indian Institute of Geomagnetism, Nanabhai Moos Marg, Navy Nagar, Colaba, Mumbai, Maharashtra 400005, India

Philip Menzel; University of Hamburg, Centre for Marine and Atmospheric Sciences, Bundesstraße 53, 20146 Hamburg, Germany

Jayashree Ratnam; Tata Institute of Fundamental Research, National Centre for Biological Sciences, GKVK Campus, Bellary Road, Canara Bank Layout, Rajiv Gandhi Nagar, Kodigehalli, Bengaluru, Karnataka 560065, India

Sushma Prasad; University of Potsdam, Institute of Earth and Environmental Sciences, Karl-Liebknecht-Straße 24/25, 14476 Potsdam, Germany

Dirk Sachse; University of Potsdam, Institute of Earth and Environmental Sciences, Karl-Liebknecht-Straße 24/25, 14476 Potsdam, Germany

Mahesh Sankaran; Tata Institute of Fundamental Research, National Centre for Biological Sciences, GKVK Campus, Bellary Road, Canara Bank Layout, Rajiv Gandhi Nagar, Kodigehalli, Bengaluru, Karnataka 560065, India

Saswati Sarkar; University of Potsdam, Institute of Earth and Environmental Sciences, Karl-Liebknecht-Straße 24/25, 14476 Potsdam, Germany

Martina Stebich; Senckenberg Research Institute, Research Station of Quaternary Palaeontology, Am Jakobskirchhof 4, 99423 Weimar, Germany

*Correspondence to nils_riedel@web.de

**SI: Chronology**

The age-depth model of the 10 m-long sediment profile is based on 23 AMS-^14^C dates from terrestrial plant macros and Gaylussite crystals ^1^ (Fig 1; SI).


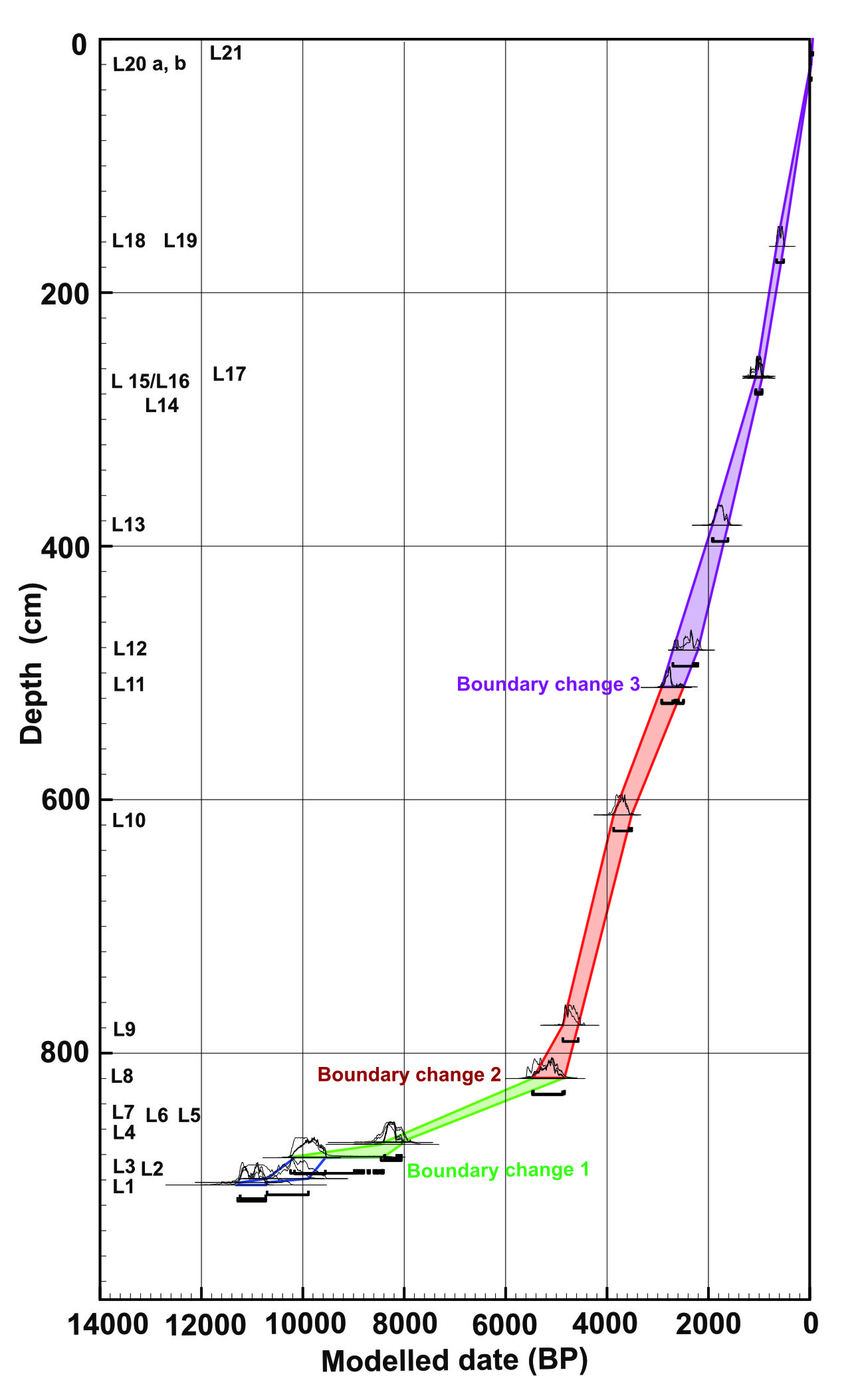


Fig. 1, SI: Chronology of the Lonar Lake composite sediment profile L23.

**Interpretation of the Lonar Lake carbonate δ^18^O-record**

The early Holocene evaporitive carbonates show less negative δ^18^O values that could be related to reduced dilution by the isotopically depleted stream inflow during periods of higher lake levels i.e. higher surface runoff as compared to groundwater inflow. However, the spatial δ^18^O isotopic variability of 4‰ in surface bulk carbonates^2^ cannot alone explain the range of variability seen in the core bulk carbonate isotopic composition. As this region does not receive any westerly precipitation, we conclude that the apparent "isotopic switch" at 6.0 kyrs-1 cal BP is related to a change in source water composition or precipitation pathways. Nevertheless, the high correlation coefficient (0.78) between the oxygen and carbon isotope values of calcium carbonate confirms their evaporitive origin^1^.

**SI: Pollen and charcoal analysis**

Tab. 1, SI: Main vegetation types, related climate parameters based on^3^ and attributed pollen types.


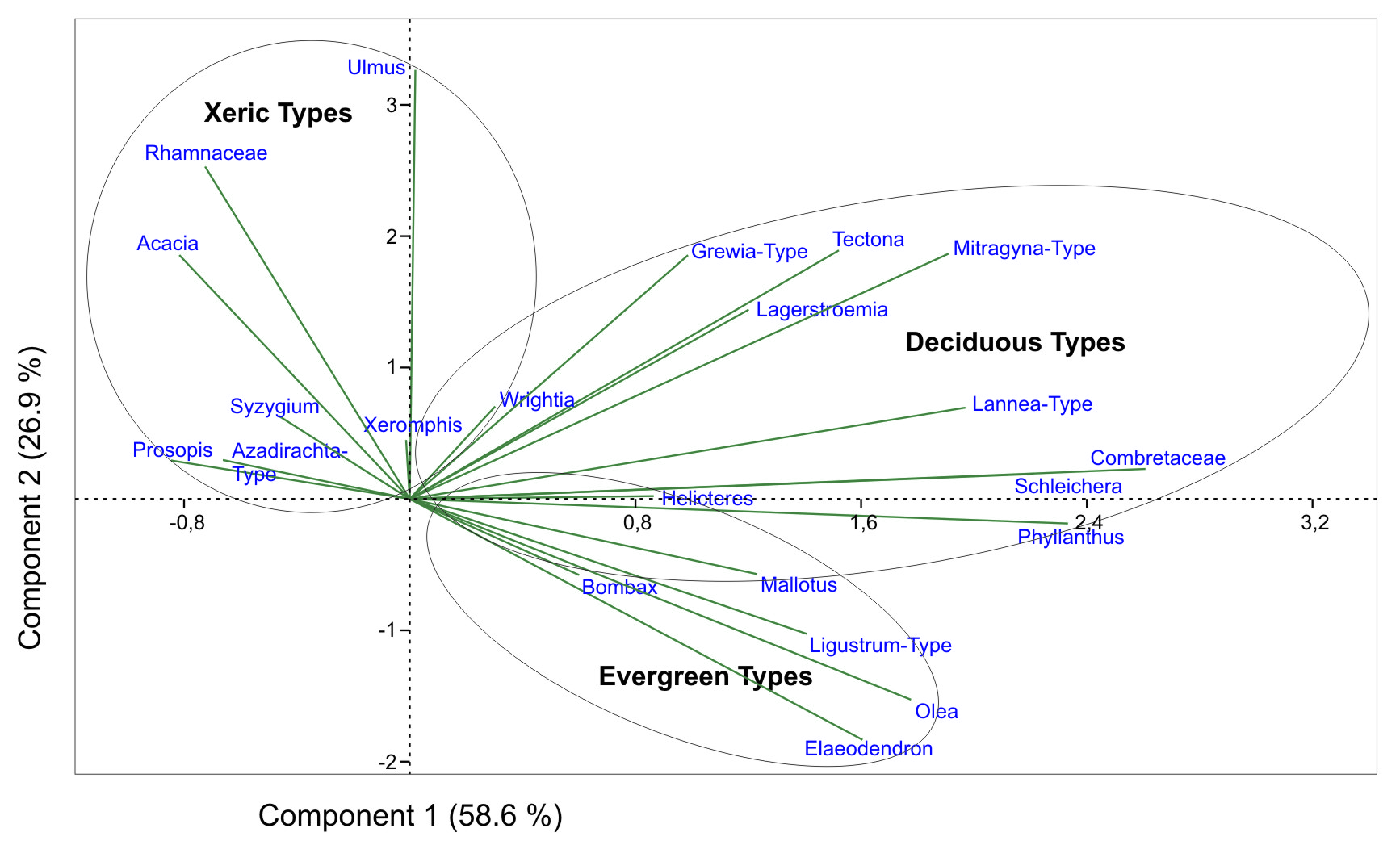


Fig. 2, SI: Results of Principal Component Analysis on the fossil tree pollen spectra.

**SI: Composite ISM-speleothem record**

Fig. 3, SI: Data coverage of the composite speleothem record.

**SI: Archaeological data**

**Table 2. Archaeological site data**

| Site | State | Latitude | Longitude | GeoRef Quality | Dating Quality | Cultural period | sub-phase | Start Date BC/AD | Finish Date BC/AD | Est. Date Median BC/AD | References |
| --- | --- | --- | --- | --- | --- | --- | --- | --- | --- | --- | --- |
| A Gokulapadu | Andhra Pradesh | 15.78472222 | 77.93472222 | 3 | ass | Neolithic |  | -2500 | -1200 | -1850 | ^12^ |
| Abbaragatte | Karnataka | 14.775 | 75.65833333 | 2 | ass | Neolithic | Ashmound IIB | -1800 | -1200 | -1500 | ^11^ |
| Abburu | Andhra Pradesh | 16.448998 | 80.162718 | 2 | ass | Neolithic |  | -2500 | -1200 | -1850 | ^15^ |
| Adam | Maharashtra | 20.98865 | 79.26999 | 1 | C14 | Microlithic (late) |  | -2000 | -1500 | -1750 | ^18^ |
| Adam | Maharashtra | 20.98865 | 79.26999 | 1 | ass | Chalcolithic |  | -1900 | -900 | -1400 | ^18^ |
| Adanur | Tamil Nadu | 12.138771 | 77.950719 | 3 | ass | Neolithic |  | -1700 | -1000 | -1350 | ^15,24^ |
| Adiyamankottai | Tamil Nadu | 12.073924 | 78.123273 | 2 | ass | Neolithic |  | -1700 | -1000 | -1350 | ^15^ |
| Akkamanahalli | Tamil Nadu | 12.15051 | 78.254879 | 2 | ass | Neolithic |  | -1700 | -1000 | -1350 | ^15^ |
| Akkammakonda | Andhra Pradesh | 14.63199537 | 77.13855455 | 1 | ass | Neolithic |  | -2500 | -1200 | -1850 | ^15,36^ |
| Aligambar | Karnataka | 18.002024 | 77.433933 | 3 | ass | Neolithic |  | -2200 | -1200 | -1700 | ^15,30^ |
| Allagadda | Andhra Pradesh | 15.69861111 | 78.4125 | 3 | ass | Neolithic |  | -1700 | -1200 | -1450 | ^12^ |
| Allur | Andhra Pradesh | 18.378 | 79.1 | 1 | ass | Neolithic |  | -2500 | -1200 | -1850 | ^15^ |
| Almel | Karnataka | 17.090375 | 76.219454 | 2 | ass | Neolithic |  | -2200 | -1200 | -1700 | ^15,37^ |
| Alugumillipadu | Andhra Pradesh | 16.573095 | 79.729827 | 2 | ass | Neolithic |  | -2500 | -1200 | -1850 | ^10,15^ |
| Amadala | Andhra Pradesh | 15.25555556 | 78.3 | 2 | ass | Neolithic |  | -1700 | -1200 | -1450 | ^12^ |
| Amalthe | Maharashtra | 21.35333333 | 74.25 | 3 | ass | Chalcolithic | Jorwe | -1500 | -1100 | -1300 | ^29^ |
| Amalthe | Maharashtra | 21.35333333 | 74.25 | 3 | ass | Early Historic |  | -350 | 300 | -25 | ^29^ |
| Amaravati | Andhra Pradesh | 16.578881 | 80.354711 | 3 | ass | Neolithic |  | -2500 | -1200 | -1850 | ^10,15,21^ |
| Amargol | Karnataka | 15.649101 | 75.397487 | 2 | ass | Neolithic |  | -2600 | -1800 | -2200 | ^15,38^ |
| Ambesangvi | Karnataka | 18.058783 | 77.266766 | 3 | ass | Neolithic |  | -2200 | -1200 | -1700 | ^15,30^ |
| Ambur | Tamil Nadu | 12.788066 | 78.716442 | 2 | ass | Neolithic |  | -1700 | -1000 | -1350 | ^15^ |
| Amirdhi | Tamil Nadu | 12.72603 | 79.058869 | 2 | ass | Neolithic |  | -1700 | -1000 | -1350 | ^11^ |
| Anacni | Karnataka | 16.94086261 | 75.92691175 | 1 | ass | Chalcolithic | Jorwe | -1500 | -1200 | -1350 | ^15^ |
| Anapur | Andhra Pradesh | 18.378 | 79.1 | 1 | ass | Neolithic |  | -2500 | -1200 | -1850 | ^15^ |
| Anaveri | Karnataka | 14.05833333 | 75.24166667 | 2 | ass | Neolithic |  | -2000 | -1200 | -1600 | ^11^ |
| Andepalle | Andhra Pradesh | 14.42252662 | 77.20255889 | 1 | ass | Neolithic |  | -2500 | -1200 | -1850 | ^15^ |
| Andhi | Maharashtra | 21.39798791 | 72.77050671 | 1 | ass | Chalcolithic | Jorwe | -1500 | -1200 | -1350 | ^27^ |
| Anegondi | Karnataka | 15.347143 | 76.492129 | 2 | ass | Neolithic |  | -2200 | -1200 | -1700 | ^15^ |
| Antargangi | Karnataka | 16.868453 | 76.279771 | 2 | ass | Neolithic |  | -2200 | -1200 | -1700 | ^15^ |
| Anturli | Maharashtra | 21.386862 | 74.713653 | 2 | ass | Chalcolithic | Salvada | -2300 | -2000 | -2150 | ^27,28^ |
| Apegaon | Maharashtra | 18.584874 | 76.260593 | 2 | C14 | Chalcolithic | Malwa-Jorwe | -1700 | -1200 | -1450 | ^16^ |
| Appukallu | Tamil Nadu | 12.859136 | 78.991607 | 2 | ass | Neolithic |  | -2000 | -1400 | -1700 | ^25^ |
| Appukallu | Tamil Nadu | 12.859136 | 78.991607 | 2 | ass | Megalithic |  | -1200 | -300 | -750 | ^25^ |
| Appukallu | Tamil Nadu | 12.859136 | 78.991607 | 2 | ass | Early Historic |  | -350 | 300 | -25 | ^25^ |
| Arabikotanur | Karnataka | 13.119419 | 78.040384 | 2 | ass | Neolithic |  | -2200 | -1200 | -1700 | ^15^ |
| Asangi | Karnataka | 16.46775621 | 75.0816878 | 1 | ass | Neolithic |  | -2200 | -1200 | -1700 | ^15^ |
| Ashrav | Gujarat | 21.515071 | 74.188462 | 2 | ass | Chalcolithic | Salvada | -2300 | -2000 | -2150 | ^27^ |
| Ashrav | Gujarat | 21.515071 | 74.188462 | 2 | ass | Early Historic |  | -350 | 300 | -25 | ^27^ |
| Asundi | Karnataka | 15.749906 | 75.023458 | 2 | ass | Neolithic |  | -2200 | -1200 | -1700 | ^15^ |
| Athani | Karnataka | 16.726325 | 75.066717 | 2 | ass | Neolithic |  | -2600 | -1800 | -2200 | ^15^ |
| Atudkhas | Madhya Pradesh | 22.114005 | 76.274419 | 3 | ass | Chalcolithic | Ahar | -2500 | -1700 | -2100 | ^31^ |
| Atudkhas | Madhya Pradesh | 22.114005 | 76.274419 | 3 | ass | Chalcolithic | Malwa | -1900 | -1500 | -1700 | ^31^ |
| Aurad | Karnataka | 18.259136 | 77.413941 | 3 | ass | Neolithic |  | -2200 | -1200 | -1700 | ^15,30^ |
| Avra | Madhya Pradesh | 24.208769 | 75.407608 | 2 | ass | Chalcolithic |  | -1700 | -1100 | -1400 | ^31^ |
| Ayanur | Karnataka | 14.15 | 75.625 | 2 | ass | Neolithic |  | -2000 | -1200 | -1600 | ^11^ |
| Azad Nagar | Madhya Pradesh | 22.69624 | 75.887493 | 3 | ass | Chalcolithic | Kaythata | -2450 | -1900 | -2175 | ^31^ |
| Azad Nagar | Madhya Pradesh | 22.69624 | 75.887493 | 3 | ass | Chalcolithic | Malwa | -1900 | -1500 | -1700 | ^31^ |
| Babhulde I | Maharashtra | 21.20694444 | 74.72083333 | 3 | ass | Chalcolithic | Malwa | -1700 | -1500 | -1600 | ^29^ |
| Babhulde II | Maharashtra | 21.2875 | 74.84166667 | 3 | ass | Chalcolithic | Salvada | -2300 | -2000 | -2150 | ^27–29^ |
| Babhulde II | Maharashtra | 21.2875 | 74.84166667 | 3 | ass | Chalcolithic | Malwa | -1700 | -1500 | -1600 | ^29^ |
| Baburdi-Shirkee | Maharashtra | 18.638068 | 74.635326 | 2 | ass | Early Historic |  | -350 | 300 | -25 | ^23^ |
| Badgal | Karnataka | 17.76111111 | 77.38055556 | 3 | ass | Neolithic |  | -2200 | -1200 | -1700 | ^15,30^ |
| Badnapur | Maharashtra | 19.79205212 | 75.77493001 | 1 | ass | Chalcolithic | Jorwe | -1500 | -1200 | -1350 | ^29^ |
| Bahal | Maharashtra | 20.589703 | 75.048487 | 3 | ass | Chalcolithic | Jorwe | -1500 | -1200 | -1350 | ^29^ |
| Bahurupa I | Gujarat | 21.521408 | 74.231174 | 3 | ass | Chalcolithic | Salvada | -2300 | -2000 | -2150 | ^27,28^ |
| Bahurupa I | Gujarat | 21.521408 | 74.231174 | 3 | ass | Chalcolithic | Late Harappan | -2000 | -1700 | -1850 | ^27^ |
| Bahurupa II | Gujarat | 21.52777778 | 74.23055556 | 3 | ass | Chalcolithic | Salvada | -2300 | -2000 | -2150 | ^27,28^ |
| Bahurupa II | Gujarat | 21.52777778 | 74.23055556 | 3 | ass | Chalcolithic | Late Harappan | -2000 | -1700 | -1850 | ^27^ |
| Bahurupa III | Gujarat | 21.524096 | 74.226903 | 3 | ass | Chalcolithic | Salvada | -2300 | -2000 | -2150 | ^28^ |
| Bahurupa III | Gujarat | 21.524096 | 74.226903 | 3 | ass | Chalcolithic | Late Harappan | -2000 | -1700 | -1850 | ^27^ |
| Bahyane I | Maharashtra | 21.40416667 | 74.47916667 | 2 | ass | Chalcolithic | Salvada | -2300 | -2000 | -2150 | ^28,29^ |
| Bahyane I | Maharashtra | 21.40416667 | 74.47916667 | 2 | ass | Chalcolithic | Late Harappan | -2000 | -1700 | -1850 | ^28,29^ |
| Bahyane I | Maharashtra | 21.40416667 | 74.47916667 | 2 | ass | Chalcolithic | Jorwe | -1500 | -1100 | -1300 | ^28,29^ |
| Bahyane II | Maharashtra | 21.414016 | 74.479736 | 2 | ass | Chalcolithic | Salvada | -2300 | -2000 | -2150 | ^28,29^ |
| Bahyane II | Maharashtra | 21.414016 | 74.479736 | 2 | ass | Chalcolithic | Late Harappan | -2000 | -1700 | -1850 | ^28,29^ |
| Bahyane II | Maharashtra | 21.414016 | 74.479736 | 2 | ass | Chalcolithic | Jorwe | -1500 | -1100 | -1300 | ^28,29^ |
| Bahyane III | Maharashtra | 21.40484 | 74.464767 | 2 | ass | Chalcolithic | Salvada | -2300 | -2000 | -2150 | ^28,29^ |
| Bahyane III | Maharashtra | 21.40484 | 74.464767 | 2 | ass | Chalcolithic | Late Harappan | -2000 | -1700 | -1850 | ^28,29^ |
| Bahyane III | Maharashtra | 21.40484 | 74.464767 | 2 | ass | Chalcolithic | Jorwe | -1500 | -1100 | -1300 | ^28,29^ |
| Balakundi | Karnataka | 15.527674 | 76.945798 | 2 | ass | Neolithic |  | -2500 | -1200 | -1850 | ^15^ |
| Baldane | Maharashtra | 21.35416667 | 74.47083333 | 2 | ass | Chalcolithic | Jorwe | -1500 | -1100 | -1300 | ^29^ |
| Balganur | Karnataka | 15.956087 | 76.75313 | 2 | ass | Neolithic |  | -2200 | -1200 | -1700 | ^15^ |
| Balijapalle | Andhra Pradesh | 14.466452 | 78.3308 | 3 | c14 | Neolithic |  | -1600 | -1300 | -1450 | ^33^ |
| Baloli | Karnataka | 17.22167372 | 75.82058521 | 1 | ass | Chalcolithic | Jorwe | -1500 | -1200 | -1350 | ^15,27^ |
| Balsane I | Maharashtra | 21.17083333 | 74.49166667 | 3 | ass | Chalcolithic | Jorwe | -1500 | -1100 | -1300 | ^29^ |
| Balsane II | Maharashtra | 21.156564 | 74.495295 | 2 | ass | Chalcolithic | Jorwe | -1500 | -1100 | -1300 | ^29^ |
| Balvand | Maharashtra | 21.28 | 74.39333333 | 3 | ass | Chalcolithic | Jorwe | -1500 | -1100 | -1300 | ^29^ |
| Banahalli | Karnataka | 13.184064 | 78.315613 | 2 | C-14 | Neolithic |  | -2150 | -1850 | -2000 | ^15^ |
| Banjaripeta | Andhra Pradesh | 18.50214263 | 83.99151179 | 2 | ass | Neolithic |  | -1700 | -1000 | -1350 | ^15^ |
| Bapur | Karnataka | 15.995306 | 77.074092 | 2 | ass | Neolithic |  | -2200 | -1200 | -1700 | ^15,30^ |
| Barangi | Andhra Pradesh | 17.47676754 | 81.90396187 | 2 | ass | Neolithic |  | -1700 | -1000 | -1350 | ^15^ |
| Bargur | Tamil Nadu | 12.542912 | 78.347779 | 2 | ass | Neolithic |  | -1700 | -1000 | -1350 | ^15^ |
| Bargur Mulavi | Tamil Nadu | 12.546644 | 78.337964 | 2 | ass | Neolithic |  | -1700 | -1000 | -1350 | ^15^ |
| Barkarwadi | Maharashtra | 18.074044 | 74.618325 | 2 | ass | Chalcolithic |  | -1700 | -1200 | -1450 | ^23^ |
| Barkhera Khurd | Madhya Pradesh | 24.218302 | 76.251843 | 2 | ass | Chalcolithic |  | -2450 | -1500 | -1975 | ^34^ |
| Basarkod | Karnataka | 16.401524 | 76.080578 | 2 | ass | Neolithic |  | -2600 | -1800 | -2200 | ^15^ |
| Bastipadu | Andhra Pradesh | 15.78888889 | 77.97222222 | 3 | ass | Neolithic |  | -2500 | -1200 | -1850 | ^12^ |
| Bastipadu | Andhra Pradesh | 15.708517 | 77.969859 | 3 | ass | Neolithic |  | -2500 | -1200 | -1850 | ^15^ |
| Battur | Karnataka | 15.13821151 | 75.60245039 | 1 | ass | Neolithic |  | -1700 | -1000 | -1350 | ^35^ |
| Beganpalli | Tamil Nadu | 11.660115 | 78.146372 | 1 | ass | Neolithic |  | -1700 | -1000 | -1350 | ^15^ |
| Belagal | Karnataka | 15.129368 | 76.83757 | 2 | ass | Neolithic |  | -2200 | -1200 | -1700 | ^15^ |
| Belavadi | Karnataka | 13.284739 | 75.996321 | 2 | ass | Neolithic |  | -2600 | -1800 | -2200 | ^15^ |
| Bellamallur | Karnataka | 14.271375 | 75.647694 | 3 | ass | Neolithic |  | -2100 | -1000 | -1550 | ^15^ |
| Bellamallur | Karnataka | 14.271375 | 75.647694 | 3 | ass | Megalithic |  | -1000 | 150 | -425 | ^15^ |
| Bellary | Karnataka | 15.17312297 | 76.95236011 | 1 | ass | Neolithic |  | -1700 | -1000 | -1350 | ^35,46^ |
| Belur | Karnataka | 14.621105 | 75.819554 | 3 | ass | Neolithic |  | -2100 | -1500 | -1800 | ^6,35^ |
| Benakanahalli | Karnataka | 14.225 | 75.725 | 2 | ass | Neolithic |  | -1800 | -800 | -1300 | ^11^ |
| Benkal | Karnataka | 15.45532392 | 76.45487183 | 1 | ass | Neolithic |  | -2500 | -1500 | -2000 | ^35^ |
| Benkanhalli | Karnataka | 16.74704788 | 76.86508146 | 1 | ass | Neolithic |  | -2200 | -1200 | -1700 | ^15,35^ |
| Benkanhalli tanda | Karnataka | 13.885951 | 76.311843 | 2 | ass | Neolithic |  | -2200 | -1200 | -1700 | ^15^ |
| Besnagar | Madhya Pradesh | 23.526739 | 77.814052 | 3 | ass | Chalcolithic |  | -1900 | -1100 | -1500 | ^31^ |
| Betavad | Maharashtra | 21.16666667 | 74.9125 | 3 | ass | Chalcolithic |  | -1700 | -1200 | -1450 | ^29^ |
| Betavad | Maharashtra | 21.16666667 | 74.9125 | 3 | ass | Early Historic |  | -350 | 300 | -25 | ^29^ |
| Bhadne | Maharashtra | 20.98333 | 74.16666667 | 2 | ass | Chalcolithic | Malwa | -1700 | -1500 | -1600 | ^27,29^ |
| Bhadne | Maharashtra | 20.98333 | 74.16666667 | 2 | ass | Chalcolithic | Jorwe | -1500 | -1200 | -1350 | ^27^ |
| Bhadvad-Khurd | Maharashtra | 21.3625 | 74.48333333 | 3 | ass | Chalcolithic | Jorwe | -1500 | -1200 | -1350 | ^29^ |
| Bhadvad-Khurd | Maharashtra | 21.3625 | 74.48333333 | 3 | ass | Early Historic |  | -350 | 300 | -25 | ^29^ |
| Bhairangi | Karnataka | 17.23257901 | 75.9841645 | 1 | ass | Chalcolithic | Jorwe | -1500 | -1200 | -1350 | ^15,27^ |
| Bhalki | Karnataka | 18.043049 | 77.20601 | 3 | ass | Neolithic |  | -2200 | -1200 | -1700 | ^15,30^ |
| Bhantnur | Karnataka | 16.566198 | 76.375305 | 2 | ass | Neolithic |  | -2200 | -1200 | -1700 | ^15^ |
| Bhatambra | Karnataka | 18.07042 | 77.161173 | 3 | ass | Neolithic |  | -2200 | -1200 | -1700 | ^15,30^ |
| Bhavani-Pada | Maharashtra | 21.41666667 | 74.09166667 | 2 | ass | Chalcolithic | Jorwe | -1500 | -1200 | -1350 | ^29^ |
| Bhavani-Pada | Maharashtra | 21.41666667 | 74.09166667 | 2 | ass | Early Historic |  | -350 | 300 | -25 | ^29^ |
| Bhimadolu | Andhra Pradesh | 16.85786252 | 81.41552871 | 2 | ass | Neolithic |  | -1700 | -1000 | -1350 | ^15^ |
| Bhojapur | Maharashtra | 19.73806839 | 74.16025546 | 1 | ass | Chalcolithic | Jorwe | -1500 | -1200 | -1350 | ^27^ |
| Bhona I | Maharashtra | 21.34 | 74.27083333 | 2 | ass | Chalcolithic | Malwa | -1700 | -1500 | -1600 | ^29^ |
| Bhona I | Maharashtra | 21.34 | 74.27083333 | 2 | ass | Early Historic |  | -350 | 300 | -25 | ^29^ |
| Bhona II | Maharashtra | 21.352199 | 74.267797 | 2 | ass | Chalcolithic | Malwa | -1700 | -1500 | -1600 | ^29^ |
| Bhona II | Maharashtra | 21.352199 | 74.267797 | 2 | ass | Early Historic |  | -350 | 300 | -25 | ^29^ |
| Bhongaon | Maharashtra | 21.05 | 74.18333333 | 2 | ass | Chalcolithic | Malwa | -1700 | -1500 | -1600 | ^29^ |
| Bhortek | Maharashtra | 21.63055556 | 74.60833333 | 3 | ass | Chalcolithic | Malwa | -1700 | -1500 | -1600 | ^29^ |
| Bhortek | Maharashtra | 21.63055556 | 74.60833333 | 3 | ass | Early Historic |  | -350 | 300 | -25 | ^27^ |
| Bhramagiri | Karnataka | 14.80364337 | 76.76325638 | 1 | C14 | Neolithic |  | -2200 | -1200 | -1700 | ^15^ |
| Bhupatipalem | Andhra Pradesh | 17.50687644 | 81.81028975 | 2 | ass | Neolithic |  | -1700 | -1000 | -1350 | ^15^ |
| Bijanuru | Andhra Pradesh | 15.66666667 | 78.43194444 | 2 | ass | Neolithic |  | -1700 | -1200 | -1450 | ^12^ |
| Bijasur | Karnataka | 16.59867418 | 76.82144214 | 1 | ass | Neolithic |  | -2200 | -1200 | -1700 | ^15,35^ |
| Bilgi | Karnataka | 16.37465899 | 75.60826897 | 1 | ass | Neolithic |  | -2200 | -1200 | -1700 | ^15^ |
| Billamarayan Gudda | Karnataka | 16.19719241 | 76.51596688 | 1 | ass | Neolithic |  | -2500 | -1200 | -1700 | ^15^ |
| Biramangala | Karnataka | 13.63410952 | 77.53130846 | 1 | ass | Neolithic |  | -2200 | -1200 | -1700 | ^15^ |
| Biswal | Karnataka | 16.38920543 | 75.40461879 | 1 | ass | Neolithic | Ashmound I/II | -2600 | -1600 | -2100 | ^15^ |
| Bobbepalle | Andhra Pradesh | 15.953757 | 80.137517 | 2 | ass | Neolithic |  | -1700 | -1000 | -1350 | ^15,21^ |
| Bodemmanuru | Andhra Pradesh | 15.06666667 | 78.41527778 | 2 | ass | Neolithic |  | -1700 | -1200 | -1450 | ^32^ |
| Boganapalli | Tamil Nadu | 12.534729 | 78.237051 | 2 | ass | Neolithic |  | -1700 | -1000 | -1350 | ^15^ |
| Bolladivalasa | Andhra Pradesh | 18.44359756 | 83.49304233 | 2 | ass | Neolithic |  | -1700 | -1000 | -1350 | ^15^ |
| Bondaladinne | Andhra Pradesh | 15.2 | 78.23472222 | 2 | ass | Neolithic |  | -1700 | -1200 | -1450 | ^32^ |
| Borale | Maharashtra | 21.47777778 | 74.37916667 | 3 | ass | Chalcolithic | Malwa | -1700 | -1500 | -1600 | ^29^ |
| Borale | Maharashtra | 21.47777778 | 74.37916667 | 3 | ass | Chalcolithic | Jorwe | -1500 | -1200 | -1350 | ^29^ |
| Borale | Maharashtra | 21.47777778 | 74.37916667 | 3 | ass | Early Historic |  | -350 | 300 | -25 | ^29^ |
| Boratvasti | Maharashtra | 18.16666667 | 74.88333333 | 2 | ass | Early Historic |  | -350 | 300 | -25 | ^23^ |
| Borgi | Karnataka | 18.139538 | 77.501566 | 2 | ass | Neolithic |  | -2200 | -1200 | -1700 | ^15^ |
| Bottadivalasa | Andhra Pradesh | 18.45697929 | 83.19697155 | 2 | ass | Neolithic |  | -1700 | -1000 | -1350 | ^15^ |
| Boyipalem | Andhra Pradesh | 17.75611116 | 83.21871686 | 2 | ass | Neolithic |  | -1700 | -1000 | -1350 | ^15^ |
| Brahmagiri | Karnataka | 14.806526 | 76.815968 | 3 | C14 | Neolithic |  | -2200 | -1200 | -1700 | ^15^ |
| Brahmanwel | Maharashtra | 21.15 | 74.20833333 | 2 | ass | Chalcolithic | Malwa | -1700 | -1500 | -1600 | ^29^ |
| Brahmanwel | Maharashtra | 21.15 | 74.20833333 | 2 | ass | Chalcolithic | Jorwe | -1500 | -1200 | -1350 | ^29^ |
| Budada | Andhra Pradesh | 16.47753 | 79.882568 | 1 | ass | Neolithic |  | -1700 | -1000 | -1350 | ^10,15^ |
| Budagavi | Andhra Pradesh | 15.05384215 | 77.22874249 | 1 | ass | Neolithic |  | -2500 | -1200 | -1850 | ^15,36^ |
| Budidapadu | Andhra Pradesh | 15.73611111 | 77.90555556 | 3 | ass | Neolithic |  | -2500 | -1200 | -1850 | ^12,15^ |
| Budigapalli | Andhra Pradesh | 18.2158 | 79.26 | 1 | ass | Neolithic | Phase I | -2500 | -1800 | -2150 | ^15,21^ |
| Budigudda | Karnataka | 13.617894 | 76.719631 | 1 | ass | Neolithic |  | -2200 | -1200 | -1700 | ^15^ |
| Budihal | Karnataka | 16.36011255 | 76.28322383 | 1 | C14 | Neolithic | Ashmound IIA | -2300 | -2200 | -2250 | ^15,53^ |
| Budihal | Karnataka | 16.36011255 | 76.28322383 | 1 | C14 | Neolithic | Ashmound IIB | -2250 | -1650 | -1950 | ^15,53^ |
| Budihal-S | Karnataka | 16.40957045 | 76.39086749 | 1 | ass | Neolithic | Ashmound I/II | -2600 | -1600 | -2100 | ^15^ |
| Budikote | Karnataka | 12.906314 | 78.138375 | 2 | ass | Neolithic |  | -2200 | -1200 | -1700 | ^15^ |
| Budili | Andhra Pradesh | 13.895889 | 77.702887 | 2 | ass | Neolithic |  | -2500 | -1200 | -1850 | ^15^ |
| Budinni | Karnataka | 16.2495596 | 76.84180716 | 1 | ass | Neolithic | Ashmound I/II | -2600 | -1600 | -2100 | ^15^ |
| Bukkacherla | Andhra Pradesh | 14.548229 | 77.524325 | 2 | ass | Neolithic |  | -2500 | -1200 | -1850 | ^15^ |
| Bukkapatna | Karnataka | 13.619857 | 76.756015 | 2 | ass | Neolithic |  | -2200 | -1200 | -1700 | ^15^ |
| Bukkasagara | Karnataka | 15.358044 | 76.526983 | 3 | C14 | Neolithic |  | -1600 | -1450 | -1525 | ^14^ |
| Bukkasagara | Karnataka | 15.358044 | 76.526983 | 3 | C14 | Iron Age |  | -1250 | -1000 | -1125 | ^14^ |
| Burivalasa | Andhra Pradesh | 18.38839792 | 83.4796606 | 2 | ass | Neolithic |  | -1700 | -1000 | -1350 | ^15^ |
| Buthamahal Hill | Andhra Pradesh | 15.0189307 | 77.16764744 | 1 | ass | Neolithic |  | -2500 | -1200 | -1850 | ^15,36^ |
| Byakod | Karnataka | 16.527254 | 76.106585 | 2 | ass | Neolithic |  | -2200 | -1200 | -1700 | ^15^ |
| Chakryapeta | Andhra Pradesh | 14.256684 | 78.495429 | 2 | ass | Neolithic |  | -2500 | -1200 | -1850 | ^15^ |
| Chalkapur | Karnataka | 17.896763 | 77.265144 | 3 | ass | Neolithic |  | -2200 | -1200 | -1700 | ^15,30^ |
| Chanddravalli | Karnataka | 14.208698 | 76.386634 | 3 | ass | Neolithic |  | -2200 | -1200 | -1700 | ^15^ |
| Chandoli | Maharashtra | 16.956921 | 73.838869 | 2 | ass | Chalcolithic | Malwa | -1700 | -1500 | -1600 | ^22^ |
| Chandoli | Maharashtra | 16.956921 | 73.838869 | 2 | ass | Chalcolithic | Jorwe | -1500 | -1200 | -1350 | ^22^ |
| Chandoli | Maharashtra | 18.95834047 | 74.15752914 | 1 | ass | Chalcolithic | Jorwe | -1500 | -1200 | -1350 | ^27^ |
| Chandru | Karnataka | 15.200484 | 76.635019 | 2 | ass | Neolithic |  | -2500 | -1200 | -1850 | ^15^ |
| Chanegaon | Karnataka | 17.01719961 | 75.92963807 | 1 | ass | Chalcolithic | Jorwe | -1500 | -1200 | -1350 | ^15^ |
| Channur | Karnataka | 14.901551 | 75.529685 | 2 | ass | Neolithic |  | -2200 | -1200 | -1700 | ^15^ |
| Chebrolu | Andhra Pradesh | 16.197109 | 80.527577 | 2 | ass | Neolithic |  | -2500 | -1200 | -1850 | ^21^ |
| Chennarayanapalli | Tamil Nadu | 12.958445 | 78.907903 | 2 | ass | Neolithic-Megalithic | | -1700 | -1000 | -1350 | ^47^ |
| Cherlopalle | Andhra Pradesh | 15.16666667 | 78.08333333 | 2 | ass | Neolithic |  | -1700 | -1200 | -1450 | ^32^ |
| Chetnepalli | Andhra Pradesh | 15.94699363 | 77.30438398 | 1 | ass | Neolithic |  | -2500 | -1200 | -1850 | ^15,36^ |
| Chevapadu | Andhra Pradesh | 16.528799 | 80.3858 | 3 | ass | Neolithic |  | -2500 | -1200 | -1850 | ^10,15^ |
| Chhadwel | Maharashtra | 20.00694444 | 74.25333333 | 3 | ass | Chalcolithic | Late Harappan | -2000 | -1700 | -1850 | ^29^ |
| Chichali | Madhya Pradesh | 22.13626 | 75.416125 | 3 | ass | Chalcolithic | Ahar | -2500 | -1700 | -2100 | ^31^ |
| Chichali | Madhya Pradesh | 22.13626 | 75.416125 | 3 | ass | Chalcolithic | Malwa | -1900 | -1500 | -1700 | ^31^ |
| Chichali | Madhya Pradesh | 22.13626 | 75.416125 | 3 | ass | Chalcolithic | Jorwe | -1500 | -1100 | -1300 | ^31^ |
| Chikalda | Madhya Pradesh | 22.084684 | 74.888456 | 3 | ass | Chalcolithic |  | -2450 | -1500 | -1975 | ^19^ |
| Chikhali | Maharashtra | 21.10972222 | 73.95 | 3 | ass | Chalcolithic | Late Harappan | -2000 | -1700 | -1850 | ^27^ |
| Chikse | Maharashtra | 20.95 | 74.14583333 | 2 | ass | Chalcolithic | Jorwe | -1500 | -1200 | -1350 | ^27,29^ |
| Chikse | Maharashtra | 20.95 | 74.14583333 | 2 | ass | Early Historic |  | -350 | 300 | -25 | ^29^ |
| Chilakalagedda | Andhra Pradesh | 18.170355 | 83.129551 | 1 | ass | Neolithic |  | -1700 | -1000 | -1350 | ^15^ |
| Chimmad | Karnataka | 16.38047757 | 75.15442001 | 1 | ass | Neolithic |  | -2200 | -1200 | -1700 | ^15^ |
| Chinchkede | Maharashtra | 25.93333333 | 74.45 | 2 | ass | Chalcolithic | Jorwe | -1500 | -1200 | -1350 | ^29^ |
| Chinchkede | Maharashtra | 25.93333333 | 74.45 | 1 |  | Early Historic |  | -350 | 300 | -25 | ^29^ |
| Chinchoda | Gujarat | 21.51 | 74.24166667 | 2 | ass | Chalcolithic | Salvada | -2300 | -2000 | -2150 | ^28,29^ |
| Chincholi | Maharashtra | 18.9746984 | 74.44106657 | 1 | ass | Chalcolithic | Jorwe | -1500 | -1200 | -1350 | ^27^ |
| Chindukuru | Andhra Pradesh | 15.63333333 | 78.45 | 2 | ass | Neolithic |  | -1700 | -1200 | -1450 | ^32^ |
| Chinnadandukonda | Andhra Pradesh | 14.87637558 | 77.65349856 | 1 | ass | Neolithic |  | -2500 | -1200 | -1850 | ^15,36^ |
| Chinnakopperla | Andhra Pradesh | 15.3125 | 78.33333333 | 2 | ass | Neolithic |  | -1700 | -1200 | -1450 | ^32^ |
| Chinnamarur | Andhra Pradesh | 15.986718 | 78.170483 | 3 | ass | Neolithic |  | -2500 | -1200 | -1850 | ^15^ |
| Chinnampalle | Andhra Pradesh | 15.71944444 | 78.26388889 | 3 | ass | Neolithic |  | -1700 | -1200 | -1450 | ^32^ |
| Chintaki | Karnataka | 18.221728 | 77.53131 | 2 | ass | Neolithic |  | -2200 | -1200 | -1700 | ^15,30^ |
| Chintalapalle | Andhra Pradesh | 15.73333333 | 78.28333333 | 2 | ass | Neolithic |  | -1700 | -1200 | -1450 | ^32^ |
| Chitta | Karnataka | 17.853464 | 77.511106 | 3 | ass | Neolithic |  | -2200 | -1200 | -1700 | ^15,30^ |
| Dahidula Khurd | Maharashtra | 21.59112941 | 73.66468031 | 1 | ass | Chalcolithic | Jorwe | -1500 | -1200 | -1350 | ^27^ |
| Dahindule | Maharashtra | 21.4 | 74.28333333 | 2 | ass | Chalcolithic | Malwa | -1700 | -1500 | -1600 | ^29^ |
| Dahindule | Maharashtra | 21.4 | 74.28333333 | 2 |  | Early Historic |  | -350 | 300 | -25 | ^29^ |
| Daimabad | Maharashtra | 19.507413 | 74.696237 | 3 | C14 | Savalda (2000-1700 BC) | | -2000 | -1700 | -1850 | ^27,28^ |
| Daimabad | Maharashtra | 19.507413 | 74.696237 | 3 | C14 | Malwa (1700-1500 BC) | | -1700 | -1500 | -1600 | ^27,28^ |
| Daimabad | Maharashtra | 19.507413 | 74.696237 | 3 | C14 | Jorwe (1500-1100 BC) | | -1500 | -1100 | -1300 | ^27,28^ |
| Damanukonda | Andhra Pradesh | 18.49377904 | 83.48467875 | 2 | ass | Neolithic |  | -1700 | -1000 | -1350 | ^15^ |
| Dangwada | Madhya Pradesh | 23.092425 | 75.520915 | 3 | ass | Chalcolithic | Kayatha | -2450 | -1900 | -2175 | ^31^ |
| Dangwada | Madhya Pradesh | 23.092425 | 75.520915 | 3 | ass | Chalcolithic | Ahar | -2000 | -1700 | -1850 | ^31^ |
| Dangwada | Madhya Pradesh | 23.092425 | 75.520915 | 3 | ass | Chalcolithic | Malwa | -1900 | -1500 | -1700 | ^31^ |
| Dangwada | Madhya Pradesh | 23.092425 | 75.520915 | 3 | ass | Early Historic (300BC-300AD) | | -300 | 300 | 0 | ^31^ |
| Darkhede | Maharashtra | 21.20694444 | 74.71666667 | 2 | ass | Chalcolithic | Malwa | -1700 | -1500 | -1600 | ^29^ |
| Darkhede | Maharashtra | 21.20694444 | 74.71666667 | 2 |  | Early Historic |  | -350 | 300 | -25 | ^29^ |
| Daroji | Karnataka | 15.46696108 | 76.91744865 | 1 | ass | Neolithic | Ashmound I/II | -2600 | -1600 | -2100 | ^15^ |
| Darsi | Andhra Pradesh | 15.771798 | 79.673199 | 2 | ass | Neolithic |  | -1700 | -1000 | -1350 | ^15,21^ |
| Dasarathuguda | Andhra Pradesh | 18.69952315 | 83.91623955 | 2 | ass | Neolithic |  | -1700 | -1000 | -1350 | ^15^ |
| Davamdinne | Andhra Pradesh | 15.81666667 | 77.59027778 | 3 | ass | Neolithic |  | -2500 | -1200 | -1850 | ^12^ |
| Deolali | Maharashtra | 19.43272039 | 74.50104564 | 1 | ass | Chalcolithic | Jorwe | -1500 | -1200 | -1350 | ^27^ |
| Devanuru | Andhra Pradesh | 15.725 | 78.26388889 | 3 | ass | Neolithic |  | -1700 | -1200 | -1450 | ^32^ |
| Devaruppala | Andhra Pradesh | 17.545026 | 79.349046 | 3 | ass | Neolithic |  | -2000 | -1000 | -1500 | ^15,21^ |
| Dhadne | Maharashtra | 20.95 | 74.21666667 | 2 | ass | Chalcolithic | Jorwe | -1500 | -1200 | -1350 | ^29^ |
| Dhadne | Maharashtra | 20.95 | 74.21666667 | 2 | ass | Early Historic |  | -350 | 300 | -25 | ^29^ |
| Dhanore | Maharashtra | 21.42083333 | 74.07916667 | 2 | ass | Chalcolithic | Jorwe | -1500 | -1200 | -1350 | ^29^ |
| Dharkarwadi | Maharashtra | 18.6 | 74.56666667 | 2 | ass | Early Historic |  | -350 | 300 | -25 | ^23^ |
| Dhoki | Maharashtra | 19.78168954 | 73.97759193 | 1 | ass | Chalcolithic | Jorwe | -1500 | -1200 | -1350 | ^27^ |
| Dhule | Maharashtra | 20.9 | 74.78333333 | 2 | ass | Chalcolithic | Late Harappan | -2000 | -1700 | -1850 | ^29^ |
| Dhulkhed | Karnataka | 17.3085405 | 75.92829067 | 1 | ass | Neolithic |  | -2200 | -1200 | -1700 | ^15^ |
| Dibbapalem | Andhra Pradesh | 17.879614 | 83.28298 | 2 | ass | Neolithic |  | -1700 | -1000 | -1350 | ^15^ |
| Didagur | Karnataka | 14.20833333 | 75.66666667 | 2 | ass | Neolithic |  | -1400 | -800 | -1100 | ^11^ |
| Doddahalla | Karnataka | 12.13335 | 77.326442 | 2 | ass | Neolithic |  | -2200 | -1200 | -1700 | ^15^ |
| Doddipadu | Andhra Pradesh | 15.74444444 | 77.93277778 | 3 | ass | Neolithic |  | -2500 | -1200 | -1850 | ^12^ |
| Donagapur | Karnataka | 18.117776 | 77.211041 | 3 | ass | Neolithic |  | -2200 | -1200 | -1700 | ^15,30^ |
| Donekal NE | Andhra Pradesh | 15.23712731 | 77.20255889 | 1 | ass | Neolithic |  | -2500 | -1200 | -1850 | ^15,36^ |
| Dornakal | Andhra Pradesh | 17.447299 | 80.154672 | 2 | ass | Neolithic |  | -2000 | -1000 | -1500 | ^15^ |
| Dronadula | Andhra Pradesh | 15.908137 | 80.136234 | 3 | ass | Neolithic |  | -1700 | -1000 | -1350 | ^15,21^ |
| Dusane | Maharashtra | 21.15333333 | 74.43333333 | 2 | ass | Chalcolithic | Jorwe | -1500 | -1200 | -1350 | ^29^ |
| Edara | Andhra Pradesh | 16.728718 | 80.754027 | 2 | ass | Neolithic |  | -1700 | -1000 | -1350 | ^10,15,21^ |
| Ekalbara | Madhya Pradesh | 21.824853 | 74.990786 | 3 | ass | Chalcolithic |  | -2450 | -1200 | -1825 | ^48^ |
| Eklara | Karnataka | 18.229301 | 77.446269 | 3 | ass | Neolithic |  | -2200 | -1200 | -1700 | ^15,30^ |
| Elamanchili | Andhra Pradesh | 17.52193088 | 82.79217421 | 2 | ass | Neolithic |  | -1700 | -1000 | -1350 | ^9^ |
| Endroy | Andhra Pradesh | 16.51255 | 80.379174 | 3 | ass | Neolithic |  | -1700 | -1000 | -1350 | ^10,15^ |
| Enugubala | Andhra Pradesh | 15.8 | 77.60277778 | 3 | ass | Neolithic |  | -2500 | -1200 | -1850 | ^12^ |
| Eran | Madhya Pradesh | 24.091883 | 78.164913 | 3 | ass | Chalcolithic | Malwa | -1900 | -1500 | -1700 | ^31^ |
| Eriyur | Tamil Nadu | 12.017225 | 77.804167 | 2 | ass | Neolithic |  | -1700 | -1000 | -1350 | ^15^ |
| Errabhayanahalli | Tamil Nadu | 12.140109 | 78.00822 | 1 | ass | Neolithic |  | -1700 | -1000 | -1350 | ^15^ |
| Erullapadu | Andhra Pradesh | 16.821939 | 80.398182 | 2 | ass | Neolithic |  | -1700 | -1000 | -1350 | ^10,15,21^ |
| Examba | Karnataka | 16.48230266 | 74.62492956 | 1 | ass | Neolithic |  | -2200 | -1200 | -1700 | ^15^ |
| French Rocks | Karnataka | 12.50821499 | 76.66143129 | 1 | ass | Neolithic |  | -2200 | -1200 | -1700 | ^15^ |
| Gadaburu | Andhra Pradesh | 17.857645 | 82.821717 | 2 | ass | Neolithic |  | -1700 | -1000 | -1350 | ^15^ |
| Gaddamakulapalli | Andhra Pradesh | 15.27361111 | 78.14444444 | 3 | ass | Neolithic |  | -1700 | -1200 | -1450 | ^12^ |
| Gadekal | Andhra Pradesh | 15.20512514 | 77.27238181 | 1 | ass | Neolithic |  | -2500 | -1200 | -1850 | ^15^ |
| Gadiganuru | Karnataka | 15.40877531 | 76.84471645 | 1 | ass | Neolithic | Ashmound I/II | -2600 | -1600 | -2100 | ^20,35^ |
| Gandluru | Andhra Pradesh | 16.430523 | 80.156075 | 2 | ass | Neolithic |  | -2500 | -2000 | -2250 | ^15,39^ |
| Gangakhedi | Madhya Pradesh | 23.623575 | 77.494389 | 2 | ass | Chalcolithic | Ahar | -2500 | -1700 | -2100 | ^31^ |
| Gangakhedi | Madhya Pradesh | 23.623575 | 77.494389 | 2 | ass | Chalcolithic | Malwa | -1900 | -1500 | -1700 | ^31^ |
| Gangdanahalli | Karnataka | 13.016378 | 77.469617 | 1 | ass | Neolithic | Ashmound I/II | -2600 | -1600 | -2100 | ^15^ |
| Gani | Andhra Pradesh | 15.66666667 | 78.43333333 | 2 | ass | Neolithic |  | -1700 | -1200 | -1450 | ^32^ |
| Garh Chandella | Chhattisgarh | 19.226951 | 81.548578 | 2 | ass | Neolithic |  | -2000 | -800 | -1400 | ^13^ |
| Gaudur | Karnataka | 16.31938252 | 76.74580065 | 1 | ass | Neolithic | Ashmound I/II | -2600 | -1600 | -2100 | ^15^ |
| Gaurimedu | Tamil Nadu | 11.87689945 | 79.80637182 | 1 | ass | Neolithic |  | -1700 | -1000 | -1350 | ^15^ |
| Gavaravaram | Andhra Pradesh | 17.115783 | 81.426874 | 3 | ass | Neolithic |  | -2000 | -1000 | -1500 | ^49^ |
| Geddakere | Karnataka | 15.19348799 | 76.5363319 | 1 | ass | Neolithic |  | -2200 | -1200 | -1700 | ^15^ |
| Ghodade | Maharashtra | 21.025 | 73.20694444 | 3 | ass | Chalcolithic | Jorwe | -1500 | -1200 | -1350 | ^29^ |
| Gidhade | Maharashtra | 21.296833 | 74.815299 | 3 | ass | Chalcolithic | Salvada | -2300 | -2000 | -2150 | ^28,29^ |
| Gidhade | Maharashtra | 21.296833 | 74.815299 | 3 | ass | Early Historic |  | -350 | 300 | -25 | ^29^ |
| Gilaulikhera | Madhya Pradesh | 26.365 | 78.4 | 1 | ass | Chalcolithic | Malwa (IB) & earlier | -2000 | -1500 | -1750 | ^31^ |
| Gokanakunda | Andhra Pradesh | 15.955631 | 79.874543 | 3 | ass | Neolithic-Megalithic-Early Historic | | -1800 | -1000 | -1400 | ^49^ |
| Gokarana | Karnataka | 14.547385 | 74.3173 | 2 | ass | Neolithic |  | -2200 | -1200 | -1700 | ^15^ |
| Gollapalli | Tamil Nadu | 12.750983 | 77.936947 | 2 | ass | Neolithic |  | -1700 | -1000 | -1350 | ^15^ |
| Gollapalli | Tamil Nadu | 11.660115 | 78.146372 | 1 | ass | Neolithic |  | -1700 | -1000 | -1350 | ^50^ |
| Gollarahatti | Karnataka | 13.419764 | 76.71219 | 2 | ass | Neolithic |  | -2200 | -1200 | -1700 | ^15^ |
| Gondas | Maharashtra | 20.96666667 | 74.31666667 | 2 | ass | Chalcolithic | Jorwe | -1500 | -1200 | -1350 | ^29^ |
| Gorantla | Andhra Pradesh | 15.6375 | 77.825 | 3 | ass | Neolithic |  | -2500 | -1200 | -1850 | ^12^ |
| Gorchinchelli | Karnataka | 18.043531 | 77.118023 | 3 | ass | Neolithic |  | -2200 | -1200 | -1700 | ^15,30^ |
| Gorkal | Karnataka | 16.000671 | 77.182044 | 2 | ass | Neolithic | Ashmound I/II | -2600 | -1600 | -2100 | ^15^ |
| Gospadu | Andhra Pradesh | 15.35 | 78.41805556 | 2 | ass | Neolithic |  | -1700 | -1200 | -1450 | ^32^ |
| Gotkhila | Maharashtra | 18.575531 | 74.542199 | 2 | ass | Chalcolithic | Jorwe | -1500 | -1100 | -1300 | ^23^ |
| Govindapalle | Andhra Pradesh | 15.05 | 78.34861111 | 2 | ass | Neolithic |  | -1700 | -1200 | -1450 | ^32^ |
| Guchimi | Andhra Pradesh | 18.51385164 | 83.39937022 | 2 | ass | Neolithic |  | -1700 | -1000 | -1350 | ^9,15^ |
| Gudekallu | Andhra Pradesh | 15.77243634 | 77.41784622 | 1 | ass | Neolithic | Ashmound I/II | -2600 | -1600 | -2100 | ^35^ |
| Gudihal | Karnataka | 15.821469 | 76.542254 | 2 | ass | Neolithic |  | -2200 | -1200 | -1700 | ^15^ |
| Gudipadu-east | Andhra Pradesh | 15.76666667 | 77.86666667 | 3 | ass | Neolithic |  | -2500 | -1200 | -1850 | ^12^ |
| Gudisagar | Karnataka | 15.601473 | 75.394257 | 3 | ass | Neolithic |  | -2200 | -1200 | -1700 | ^15^ |
| Gudivola | Andhra Pradesh | 17.866832 | 83.322208 | 2 | ass | Neolithic |  | -1700 | -1000 | -1350 | ^15^ |
| Gudur | Andhra Pradesh | 15.775 | 77.80416667 | 3 | ass | Neolithic |  | -2500 | -1200 | -1850 | ^12^ |
| Gugihal | Karnataka | 17.05264179 | 76.08776471 | 1 | ass | Chalcolithic | Jorwe | -1500 | -1200 | -1350 | ^27^ |
| Gulapalayam | Andhra Pradesh | 15.19930656 | 77.45275768 | 1 | ass | Neolithic |  | -2500 | -1200 | -1850 | ^15,36^ |
| Gunavadi | Maharashtra | 18.131131 | 74.582919 | 2 | ass | Chacolithic |  | -1700 | -1200 | -1450 | ^23^ |
| Gundupapala | Andhra Pradesh | 15.325 | 78.375 | 2 | ass | Neolithic |  | -1700 | -1200 | -1450 | ^32^ |
| Gunjalapadu | Andhra Pradesh | 15.18888889 | 78.3125 | 3 | ass | Neolithic |  | -1700 | -1200 | -1450 | ^32^ |
| Guttahalli | Karnataka | 13.031542 | 78.354541 | 2 | ass | Neolithic |  | -2200 | -1200 | -1700 | ^15^ |
| Guttur | Tamil Nadu | 12.444037 | 78.420578 | 2 | ass | Neolithic |  | -1700 | -1000 | -1350 | ^15^ |
| Hadargeri | Karnataka | 14.738508 | 75.705482 | 2 | ass | Neolithic | III/IV/Jorwe-oid | -1800 | -1000 | -1400 | ^15^ |
| Hadonahalli | Karnataka | 14.289 | 75.62579 | 2 | ass | Neolithic |  | -1800 | -800 | -1300 | ^11^ |
| Hajjargi | Karnataka | 17.825 | 77.34583333 | 3 | ass | Neolithic |  | -2200 | -1200 | -1700 | ^15,30^ |
| Halakundi | Karnataka | 15.11784649 | 76.87380933 | 1 | ass | Neolithic | Ashmound I/II | -2600 | -1600 | -2100 | ^15,35^ |
| Halasabalu | Karnataka | 14.51523 | 75.778508 | 2 | ass | Neolithic |  | -2500 | -1200 | -1850 | ^15^ |
| Halekota | Karnataka | 15.56587688 | 76.92035794 | 1 | ass | Neolithic |  | -2200 | -1200 | -1700 | ^15^ |
| Halelingapur | Karnataka | 14.1 | 75.70833333 | 2 | ass | Neolithic |  | -1400 | -800 | -1100 | ^11^ |
| Hallikhed (B) | Karnataka | 17.846346 | 77.265 | 3 | ass | Neolithic |  | -2200 | -1200 | -1700 | ^15,30^ |
| Hallur | Karnataka | 14.36583 | 75.62306 | 3 | AMS | Neolithic | Ashmound IIB | -2000 | -1750 | -1875 | ^15,53^ |
| Hallur | Karnataka | 14.36583 | 75.62306 | 3 | AMS | Neolithic | Ashmound III | -1750 | -1350 | -1550 | ^15,53^ |
| Hallur | Karnataka | 14.36583 | 75.62306 | 3 | AMS | Neolithic | Ashmound IV | -1350 | -800 | -1075 | ^15,53^ |
| Hanagandi | Karnataka | 16.42411689 | 75.0816878 | 1 | ass | Neolithic |  | -2200 | -1200 | -1700 | ^15^ |
| Hanchinal | Karnataka | 15.685753 | 75.238271 | 2 | ass | Neolithic |  | -2200 | -1200 | -1700 | ^15^ |
| Hanumantaraopeta | Andhra Pradesh | 14.925626 | 78.384854 | 3 | AMS | Neothic (1700-1400 BC) | | -1700 | -1400 | -1550 | ^15,53^ |
| Haraganahalli | Karnataka | 14.34166667 | 75.64166667 | 2 | ass | Neolithic |  | -2200 | -1200 | -1700 | ^11^ |
| Harnur | Karnataka | 16.955126 | 76.720524 | 2 | ass | Neolithic |  | -2200 | -1200 | -1700 | ^15^ |
| Hatnur | Maharashtra | 20.168641 | 75.141476 | 3 | ass | Chalcolithic | Late Harappan | -2000 | -1700 | -1850 | ^29^ |
| Hatnur | Maharashtra | 20.168641 | 75.141476 | 3 | ass | Chalcolithic | Jorwe | -1500 | -1200 | -1350 | ^29^ |
| Hatnur | Maharashtra | 20.168641 | 75.141476 | 3 | ass | Chalcolithic | Malwa | -1700 | 1500 | -100 | ^29^ |
| Hatoda | Gujarat | 21.52666667 | 74.20833333 | 3 | ass | Chalcolithic | Salvada | -2300 | -2000 | -2150 | ^27^ |
| Hatoda | Gujarat | 21.52666667 | 74.20833333 | 3 | ass | Early Historic |  | -350 | 300 | -50 | ^29^ |
| Hattibelagallu | Andhra Pradesh | 15.365369 | 77.215701 | 3 | AMS | Neolithic (2000-1400 BC) | | -2000 | -1400 | -1700 | ^15,53^ |
| Havalgi | Andhra Pradesh | 15.06257002 | 77.12982669 | 1 | ass | Neolithic |  | -2500 | -1200 | -1850 | ^15,36^ |
| Hemmige | Karnataka | 12.17364685 | 76.94945082 | 1 | ass | Neolithic |  | -2200 | -1200 | -1700 | ^15^ |
| Hingnir | Gujarat | 21.51111111 | 74.29166667 | 3 | ass | Chalcolithic | Salvada | -2300 | -2000 | -2150 | ^28,29^ |
| Hingoni-Budruk | Maharashtra | 21.334078 | 74.850021 | 3 | ass | Chalcolithic | Late Harappan | -2000 | -1700 | -1850 | ^38^ |
| Hippalagaon | Karnataka | 18.170558 | 77.341816 | 3 | ass | Neolithic |  | -2200 | -1200 | -1700 | ^15,30^ |
| Hippargi | Karnataka | 16.55794415 | 75.23878937 | 1 | ass | Neolithic |  | -2200 | -1200 | -1700 | ^15,35^ |
| Hiregudda | Karnataka | 15.187103 | 76.974387 | 3 | AMS | Neolithic (2000-1300 BC) | | -2000 | -1300 | -1650 | ^15,53^ |
| Hirehal | Karnataka | 15.79722 | 75.76396 | 2 | ass | Neolithic |  | -2600 | -1800 | -2200 | ^15^ |
| Hirur | Karnataka | 15.871429 | 75.152333 | 2 | ass | Neolithic |  | -2500 | -1200 | -1850 | ^15^ |
| Hole Hanasavadi | Karnataka | 13.94166667 | 75.625 | 2 | ass | Neolithic |  | -1800 | -800 | -1300 | ^11^ |
| Hole-Alur | Karnataka | 15.826159 | 75.65076 | 2 | ass | Neolithic |  | -2500 | -1200 | -1850 | ^15^ |
| Holebenavalli | Karnataka | 13.94166667 | 75.62083333 | 2 | ass | Neolithic | III/IV | -1400 | -800 | -1100 | ^11,15^ |
| Holehatti | Karnataka | 14.025 | 75.675 |  | ass | Neolithic |  | -1800 | -1200 | -1500 | ^11,15^ |
| Honnavar | Karnataka | 14.283054 | 74.445294 | 2 | ass | Neolithic |  | -2500 | -1200 | -1850 | ^15^ |
| Hosahalli | Karnataka | 12.878539 | 77.531901 | 1 | ass | Neolithic |  | -2200 | -1200 | -1700 | ^15^ |
| Hosahalli | Karnataka | 14.13333333 | 75.71666667 | 2 | ass | Neolithic |  | -1800 | -800 | -1300 | ^11^ |
| Hosalli | Karnataka | 14.780699 | 75.355543 | 2 | ass | Neolithic |  | -2500 | -1200 | -1850 | ^15^ |
| Hulagbal | Karnataka | 16.410305 | 76.242456 | 2 | ass | Neolithic |  | -2500 | -1200 | -1850 | ^15^ |
| Hulikallu | Andhra Pradesh | 14.614772 | 77.07427 | 2 | ass | Neolithic |  | -2500 | -1200 | -1850 | ^15^ |
| Hunsgi | Karnataka | 16.460402 | 76.516432 | 2 | ass | Neolithic |  | -2500 | -1200 | -1850 | ^15^ |
| Huvinhalli | Karnataka | 16.587108 | 76.434584 | 2 | ass | Neolithic |  | -2500 | -1200 | -1850 | ^15^ |
| Idukallu | Andhra Pradesh | 14.53889815 | 77.06000377 | 1 | ass | Neolithic |  | -2500 | -1200 | -1850 | ^15^ |
| Illuru Kothapeta | Andhra Pradesh | 15.24166667 | 78.2625 | 3 | ass | Neolithic |  | -1700 | -1200 | -1450 | ^32^ |
| Inamgaon | Maharashtra | 18.60806 | 74.54944 | 3 | C14 | Malwa |  | -1700 | -1500 | -1600 | ^27,28^ |
| Inamgaon | Maharashtra | 18.60806 | 74.54944 | 3 | C14 | Early Jorwe |  | -1500 | -1200 | -1350 | ^27,28^ |
| Inamgaon | Maharashtra | 18.60806 | 74.54944 | 3 | C14 | Chalcolithic | Jorwe | -1500 | -900 | -1200 | ^27,28^ |
| Inamgaon | Maharashtra | 18.60806 | 74.54944 | 3 | C14 | Late Jorwe |  | -1200 | -900 | -1050 | ^27,28^ |
| Indihalla | Karnataka | 14.289711 | 75.249176 | 2 | ass | Neolithic |  | -2500 | -1200 | -1850 | ^15^ |
| Ingalgi | Karnataka | 15.988591 | 76.124971 | 2 | ass | Chalcolithic | Jorwe-like | -1500 | -800 | -1150 | ^15,27^ |
| Injedu | Andhra Pradesh | 15.095593 | 78.422129 | 3 | AMS | Neolithic |  | -1700 | -1400 | -1550 | ^15,15^ |
| Ippamanuguda | Andhra Pradesh | 18.59079659 | 83.91289412 | 2 | ass | Neolithic |  | -1700 | -1000 | -1350 | ^9,15^ |
| Ite | Maharashtra | 17.6769694 | 75.51796353 | 1 | ass | Chalcolithic | Jorwe | -1500 | -1200 | -1350 | ^27^ |
| Itigyal | Karnataka | 18.29863 | 77.511073 | 3 | ass | Neolithic |  | -2200 | -1200 | -1700 | ^15,30^ |
| Itlampalle | Andhra Pradesh | 14.614772 | 77.07427 | 1 | ass | Neolithic |  | -2500 | -1200 | -1850 | ^15^ |
| Jagadal | Karnataka | 16.41829832 | 75.14278286 | 1 | ass | Neolithic |  | -2200 | -1200 | -1700 | ^15,35^ |
| Jagarampalli | Andhra Pradesh | 17.41989519 | 81.86883482 | 2 | ass | Neolithic |  | -1700 | -1000 | -1350 | ^9,15^ |
| Jalakanuru | Andhra Pradesh | 15.7 | 78.31666667 | 2 | ass | Neolithic |  | -1700 | -1200 | -1450 | ^32^ |
| Jalampalle | Andhra Pradesh | 18.105259 | 82.387547 | 2 | ass | Neolithic |  | -1700 | -1000 | -1350 | ^15^ |
| Jallapur | Karnataka | 14.967746 | 75.401448 | 2 | ass | Neolithic |  | -2500 | -1200 | -1850 | ^15^ |
| Jalod | Madhya Pradesh | 23.471962 | 75.179636 | 2 | ass | Chalcolithic |  | -2450 | -1500 | -1975 | ^34^ |
| Jami | Andhra Pradesh | 18.0270912 | 83.23377131 | 2 | ass | Neolithic |  | -1700 | -1000 | -1350 | ^9,15^ |
| Jangaon | Andhra Pradesh | 18.378 | 79.1 | 1 | ass | Neolithic |  | -2500 | -1200 | -1850 | ^15^ |
| Jattinga Rameshwar | Karnataka | 14.849704 | 76.790921 | 3 | ass | Neolithic |  | -2500 | -1200 | -1850 | ^15^ |
| Jirankalgi | Karnataka | 17.23257901 | 75.6924481 | 1 | ass | Chalcolithic | Jorwe | -1500 | -1200 | -1350 | ^15,27^ |
| Joladarasi | Andhra Pradesh | 15.34166667 | 78.03055556 | 3 | ass | Neolithic |  | -1700 | -1200 | -1450 | ^32^ |
| Jonnalagadda | Andhra Pradesh | 16.376951 | 80.459584 | 2 | ass | Neolithic |  | -1700 | -1000 | -1350 | ^15,21^ |
| Jorwe | Maharashtra | 19.543602 | 74.281354 | 3 | ass | Chalcolithic | Jorwe | -1500 | -1100 | -1300 | ^27,28^ |
| Julakallu | Andhra Pradesh | 15.80277778 | 77.76666667 | 3 | ass | Neolithic |  | -2500 | -1200 | -1850 | ^12^ |
| K**.** Nagalapuram | Andhra Pradesh | 15.75972222 | 77.91944444 | 3 | ass | Neolithic |  | -2500 | -1200 | -1850 | ^12^ |
| Kadambapur | Andhra Pradesh | 18.488899 | 79.329089 | 2 | ass | Neolithic | Phase I? | -2500 | -1400 | -1950 | ^15,21,51^ |
| Kadatur | Tamil Nadu | 12.086036 | 78.288466 | 2 | ass | Neolithic |  | -1700 | -1000 | -1350 | ^15,24^ |
| Kadebakele | Karnataka | 15.364014 | 76.502817 | 3 | ass | Neolithic |  | -2200 | -1200 | -1700 | ^7^ |
| Kadebakele | Karnataka | 15.364014 | 76.502817 | 3 | C14 | Iron Age |  | -850 | -550 | -700 | ^7^ |
| Kadit | Maharashtra | 19.57994175 | 74.54739311 | 1 | ass | Chalcolithic | Jorwe | -1500 | -1200 | -1350 | ^27^ |
| Kadwad | Karnataka | 17.821455 | 77.407693 | 3 | ass | Neolithic |  | -2200 | -1200 | -1700 | ^15,30^ |
| Kaidala | Karnataka | 13.299441 | 77.079928 | 2 | ass | Neolithic |  | -2200 | -1200 | -1700 | ^24^ |
| Kakarawada | Andhra Pradesh | 15.04861111 | 78.42638889 | 3 | ass | Neolithic |  | -1700 | -1200 | -1450 | ^32^ |
| Kakkera | Karnataka | 16.38629615 | 76.5857898 | 1 | ass | Neolithic | Ashmound I/II | -2600 | -1600 | -2100 | ^40^ |
| Kakrehta | Madhya Pradesh | 23.625238 | 80.03499 | 3 | ass | Chalcolithic |  | -2000 | -1100 | -1550 | ^31^ |
| Kakubalu | Karnataka | 15.35058955 | 76.74580065 | 1 | ass | Neolithic | Ashmound I/II | -2600 | -1600 | -2100 | ^20,35^ |
| Kalakonda | Andhra Pradesh | 16.801693 | 78.62721 | 2 | ass | Neolithic |  | -2000 | -1000 | -1500 | ^24^ |
| Kalas | Maharashtra | 18.166667 | 74.566667 | 2 | ass | Early Hisoric |  | -350 | 300 | -25 | ^23^ |
| Kalaskarwadi | Maharashtra | 18.556409 | 74.561001 | 2 | ass | Early Hisoric |  | -350 | 300 | -25 | ^23^ |
| Kaldurga | Karnataka | 13.72429745 | 75.82355629 | 1 | ass | Neolithic |  | -2200 | -1200 | -1700 | ^24^ |
| Kalianimankoil | Tamil Nadu | 11.660115 | 78.146372 | 1 | ass | Neolithic |  | -1700 | -1000 | -1350 | ^24^ |
| Kallerimalai | Tamil Nadu | 12.834926 | 78.757862 | 2 | ass | Neolithic |  | -1700 | -1000 | -1350 | ^25^ |
| Kallolli | Karnataka | 16.48521194 | 75.22133364 | 1 | ass | Neolithic |  | -2500 | -1200 | -1850 | ^35^ |
| Kallur | Karnataka | 16.19719241 | 77.25783537 | 1 | ass | Neolithic |  | -2200 | -1200 | -1700 | ^24^ |
| Kalugotla | Andhra Pradesh | 15.89305556 | 77.96805556 | 3 | ass | Neolithic |  | -2500 | -1200 | -1850 | ^12^ |
| Kalyandurg | Andhra Pradesh | 14.58544676 | 77.11237096 | 1 | ass | Neolithic |  | -2500 | -1200 | -1850 | ^15,36^ |
| Kambadahal | Andhra Pradesh | 15.82777778 | 77.625 | 3 | ass | Neolithic |  | -2600 | -1700 | -2150 | ^12^ |
| Kambampadu | Andhra Pradesh | 16.987693 | 80.65049 | 2 | ass | Neolithic |  | -1700 | -1000 | -1350 | ^21^ |
| Kamepalli | Andhra Pradesh | 16.566028 | 79.636108 | 2 | ass | Neolithic |  | -1700 | -1000 | -1350 | ^10^ |
| Kanaji | Karnataka | 17.91374 | 77.295956 | 3 | ass | Neolithic |  | -2200 | -1200 | -1700 | ^15,30^ |
| Kanakagiri | Karnataka | 15.54842115 | 76.43159752 | 1 | ass | Neolithic |  | -2200 | -1200 | -1700 | ^35^ |
| Kanakavidu | Andhra Pradesh | 15.85 | 77.56388889 | 3 | ass | Neolithic |  | -2500 | -1200 | -1850 | ^12^ |
| Kanakavidupeta | Andhra Pradesh | 15.8625 | 77.56666667 | 3 | ass | Neolithic |  | -2500 | -1200 | -1850 | ^12^ |
| Kanala | Andhra Pradesh | 15.11388889 | 78.28194444 | 2 | ass | Neolithic |  | -1700 | -1200 | -1450 | ^32^ |
| Kanciagara Bellagllu | Karnataka | 15.61824406 | 77.08618737 | 1 | ass | Neolithic | Ashmound I/II | -2600 | -1600 | -2100 | ^15^ |
| Kandhra | Maharashtra | 21.3407608 | 74.38717258 | 1 | ass | Chalcolithic | Jorwe | -1500 | -1200 | -1350 | ^27^ |
| Kanigiri | Andhra Pradesh | 15.405365 | 79.514531 | 2 | ass | Neolithic |  | -2500 | -1200 | -1850 | ^15^ |
| Kannekolur | Karnataka | 16.68595283 | 76.9290858 | 1 | ass | Neolithic |  | -2500 | -800 | -1650 | ^15,35^ |
| Kannolli | Karnataka | 16.858577 | 76.146654 | 2 | ass | Neolithic |  | -2500 | -1000 | -1750 | ^15,41^ |
| Kanyathirtham | Andhra Pradesh | 14.69018113 | 78.50010143 | 1 | ass | Neolithic |  | -2500 | -1200 | -1850 | ^15,33^ |
| Kaothe | Maharashtra | 21.005024 | 74.278597 | 2 | C14 | Chalcolithic, ca. 1900 bc (antiquity of seeds uncertain) | Late Harappan | -2000 | -1700 | -1850 | ^27,29^ |
| Karakmukkala | Andhra Pradesh | 15.10039077 | 77.15601028 | 1 | ass | Neolithic |  | -2500 | -1200 | -1850 | ^15^ |
| Karao | Maharashtra | 17.24348429 | 74.16025546 | 1 | ass | Chalcolithic | Jorwe | -1500 | -1200 | -1350 | ^27^ |
| Karapakala | Andhra Pradesh | 15.885849 | 78.137455 | 2 | ass | Neolithic |  | -2500 | -1200 | -1850 | ^15,21^ |
| Karondhia | Madhya Pradesh | 22.071172 | 74.826454 | 3 | ass | Chalcolithic |  | -2450 | -1500 | -1975 | ^19^ |
| Kasipatnam | Andhra Pradesh | 18.17182 | 83.07836 | 2 | ass | Neolithic |  | -1700 | -1000 | -1350 | ^9,15^ |
| Katamadevudu hill | Andhra Pradesh | 14.50398669 | 77.65931714 | 1 | ass | Neolithic |  | -2500 | -1200 | -1850 | ^15,36^ |
| Katapur | Andhra Pradesh | 18.161667 | 80.395112 | 2 | ass | Neolithic |  | -2000 | -1200 | -1600 | ^15^ |
| Kavital | Karnataka | 16.100289 | 76.795114 | 2 | ass | Neolithic | Ashmound I/II | -2600 | -1600 | -2100 | ^15^ |
| Kawlet | Karnataka | 14.51443655 | 75.68426914 | 1 | ass | Chalcolithic | Jorwe | -1500 | -1200 | -1350 | ^27^ |
| Kayatha | Madhya Pradesh | 23.241279 | 76.017479 | 3 | C14 | Chalcolithic 2300-1500 BC | | -2300 | -1500 | -1900 | ^31^ |
| Kendatti | Karnataka | 13.131166 | 78.014132 | 2 | ass | Neolithic |  | -2500 | -800 | -1650 | ^15^ |
| Kesarapali | Andhra Pradesh | 16.7721499 | 81.03716897 | 1 | ass | Neolithic |  | -1700 | -1000 | -1350 | ^10,15^ |
| Khadarmadalgi | Karnataka | 14.641924 | 75.516619 | 2 | ass | Neolithic |  | -2200 | -1200 | -1700 | ^15^ |
| Khairwada | Madhya Pradesh | 21.563393 | 77.793914 | 3 | ass | Early Historic |  | -300 | 300 | 0 | ^31^ |
| Khanapur | Andhra Pradesh | 19.69 | 78.5 | 1 | ass | Neolithic |  | -1700 | -1000 | -1350 | ^15^ |
| Khaparkhera | Madhya Pradesh | 22.075467 | 74.854285 | 3 | ass | Chalcolithic |  | -2450 | -1500 | -1975 | ^19^ |
| Kharde-Budruk | Maharashtra | 21.26 | 74.57083333 | 3 | ass | Chalcolithic | Late Harappan | -2000 | -1700 | -1850 | ^29^ |
| Kharde-Budruk | Maharashtra | 21.26 | 74.57083333 | 3 | ass | Chalcolithic | Malwa | -1700 | -1500 | -1600 | ^29^ |
| Kharde-Budruk | Maharashtra | 21.26 | 74.57083333 | 3 | ass | Chalcolithic | Jorwe | -1500 | -1200 | -1350 | ^29^ |
| Khed | Maharashtra | 18.379323 | 74.802877 | 3 | C14/ass | Chalcolithic | Malwa-Jorwe | -1700 | -1200 | -1450 | ^22^ |
| Khedgi | Karnataka | 17.18350522 | 76.12320689 | 1 | ass | Chalcolithic | Jorwe | -1500 | -1200 | -1350 | ^27^ |
| Khedinema | Madhya Pradesh | 22.435 | 77.02833333 | 2 | ass | Chalcolithic | Malwa | -1900 | -1500 | -1700 | ^31^ |
| Khedle Parmanand | Maharashtra | 19.42999407 | 74.74641457 | 1 | ass | Chalcolithic | Jorwe | -1500 | -1200 | -1350 | ^27^ |
| Khini-Ranjol | Karnataka | 17.794995 | 77.313869 | 2 | ass | Neolithic |  | -2200 | -1200 | -1700 | ^15,30^ |
| Kittur | Karnataka | 15.595011 | 74.788241 | 2 | ass | Neolithic |  | -2200 | -1200 | -1700 | ^15^ |
| Kodagallu | Andhra Pradesh | 12.770765 | 78.312139 | 1 | ass | Neolithic |  | -1700 | -1000 | -1350 | ^32^ |
| Kodangipatti | Tamil Nadu | 9.993371 | 77.431043 | 2 | ass | Neolithic |  | -1700 | -1000 | -1350 | ^15^ |
| Kodekal | Karnataka | 16.36884042 | 76.44323468 | 1 | ass | Neolithic | Ashmound I/II | -2600 | -1600 | -2100 | ^15^ |
| Kokapet | Andhra Pradesh | 17.390036 | 78.347805 | 2 | ass | Neolithic |  | -2500 | -1200 | -1850 | ^21^ |
| Kolakonda | Andhra Pradesh | 17.712292 | 79.145649 | 1 | ass | Neolithic | Phase I? | -2500 | -1200 | -1850 | ^21^ |
| Kolda | Maharashtra | 21.16192608 | 73.92220231 | 1 | ass | Chalcolithic | Jorwe | -1500 | -1200 | -1350 | ^27^ |
| Kolimipalayam | Karnataka | 14.23342289 | 77.03963876 | 1 | ass | Neolithic | Ashmound I/II | -2600 | -1600 | -2100 | ^15^ |
| Kondabuchampeta | Andhra Pradesh | 18.52890609 | 83.34249787 | 2 | ass | Neolithic |  | -1700 | -1400 | -1350 | ^9,15^ |
| Kondramutla | Andhra Pradesh | 16.119423 | 79.749957 | 2 | ass | Neolithic |  | -1700 | -1000 | -1350 | ^21^ |
| Konduru | Andhra Pradesh | 16.676276 | 80.570727 | 3 | ass | Neolithic |  | -1700 | -1000 | -1350 | ^10,15,21^ |
| Konidene | Andhra Pradesh | 15.814532 | 79.967626 | 1 | ass | Neolithic |  | -1700 | -1000 | -1350 | ^15,21^ |
| Konnur | Karnataka | 16.11573234 | 74.69184319 | 1 | ass | Neolithic | Ashmound I/II | -2600 | -1600 | -2100 | ^15^ |
| Konnur | Karnataka | 16.201724 | 74.745597 | 2 | ass | Neolithic |  | -2200 | -1200 | -1700 | ^15^ |
| Kontalpadu | Andhra Pradesh | 15.63333333 | 78.15 | 2 | ass | Neolithic |  | -1700 | -1200 | -1450 | ^32^ |
| Kopargaon | Maharashtra | 19.8662055 | 74.44106657 | 1 | ass | Chalcolithic | Jorwe | -1500 | -1200 | -1350 | ^27^ |
| Koparli | Maharashtra | 21.4166667 | 74.42916667 | 2 | ass | Chalcolithic | Salvada | -2300 | -2000 | -2150 | ^28,29^ |
| Koparli | Maharashtra | 21.4166667 | 74.42916667 | 2 | ass | Chalcolithic | Malwa | -1700 | -1500 | -1600 | ^28,29^ |
| Korat [=Korit] | Maharashtra | 21.503005 | 74.35215 | 3 | ass | Chalcolithic | Salvada | -2300 | -2000 | -2150 | ^28,29^ |
| Korat [=Korit] | Maharashtra | 21.503005 | 74.35215 | 3 | ass | Chalcolithic | Late Harappan | -2000 | -1700 | -1850 | ^29^ |
| Koregaon | Maharashtra | 18.647099 | 74.058092 | 3 | ass | Chalcolithic | Jorwe | -1500 | -1200 | -1350 | ^22^ |
| Korhale Khurd | Maharashtra | 18.086232 | 74.36932 | 3 | ass | Chalcolthic |  | -1700 | -1200 | -1450 | ^23^ |
| Kotehalu | Karnataka | 14.1375 | 75.70138889 | 2 | ass | Neolithic |  | -2100 | -1200 | -1650 | ^11^ |
| Kothali [=Kothli] | Maharashtra | 21.39027778 | 74.11666667 | 2 | ass | Chalcolithic | Jorwe | -1500 | -1200 | -1350 | ^27,28^ |
| Kothali [=Kothli] | Maharashtra | 21.39027778 | 74.11666667 | 2 | ass | Early Hisoric |  | -350 | 300 | -25 | ^29^ |
| Kotra | Madhya Pradesh | 22.940841 | 76.34704 | 2 | ass | Chalcolithic | Malwa & Pre | -2000 | -1500 | -1750 | ^31^ |
| Kovalanpattal | Tamil Nadu | 9.903688 | 78.099072 | 2 | ass | Neolithic |  | -1700 | -1000 | -1350 | ^15^ |
| Krishnarur | Karnataka | 12.36275058 | 76.81853285 | 1 | ass | Neolithic |  | -2200 | -1200 | -1700 | ^15^ |
| Kudachi | Karnataka | 16.56667201 | 74.93331411 | 1 | ass | Neolithic | Ashmound I/II | -2600 | -1600 | -2100 | ^15^ |
| Kudatini | Karnataka | 15.186115 | 76.711714 | 3 | ass | Neolithic | Ashmound I/II | -2600 | -1600 | -2100 | ^15^ |
| Kudaveli | Andhra Pradesh | 15.961468 | 78.216331 | 2 | ass | Neolithic |  | -2500 | -1200 | -1850 | ^15,21^ |
| Kukarmunda | Gujarat | 21.5125 | 74.125 | 2 | ass | Chalcolithic | Salvada | -2300 | -2000 | -2150 | ^28,29^ |
| Kullolli | Karnataka | 16.46193764 | 75.13987357 | 1 | ass | Neolithic |  | -2200 | -1200 | -1700 | ^35^ |
| Kumrej | Maharashtra | 20.25 | 74.75833333 | 2 | ass | Chalcolithic | Malwa | -1700 | -1500 | -1600 | ^29^ |
| Kumrej | Maharashtra | 20.25 | 74.75833333 | 2 | ass | Chalcolithic | Jorwe | -1500 | -1200 | -1350 | ^29^ |
| Kumshi | Karnataka | 17.13443143 | 76.33858628 | 1 | ass | Chalcolithic | Jorwe | -1500 | -1200 | -1350 | ^29^ |
| Kunbev | Karnataka | 14.6543 | 75.592834 | 3 | ass | Chalcolithic |  | -1800 | -1000 | -1400 | ^6,35^ |
| Kundurpi | Andhra Pradesh | 14.34979441 | 77.03672947 | 1 | ass | Neolithic |  | -2500 | -1200 | -1850 | ^15^ |
| Kupgal | Karnataka | 15.23712731 | 77.01927374 | 1 | ass | Neolithic | Ashmound I/II | -2600 | -1600 | -2100 | ^15,42^ |
| Kurikuppa | Karnataka | 15.34186169 | 76.89708363 | 1 | ass | Neolithic | Ashmound I/II | -2600 | -1600 | -2100 | ^20,35^ |
| Kurkunti | Karnataka | 16.21173885 | 76.87380933 | 1 | ass | Neolithic | Ashmound I/II | -2600 | -1600 | -2100 | ^15^ |
| Kurugodu | Karnataka | 15.351914 | 76.838124 | 3 | AMS | Neolithic (2000-1400 BC) | | -2000 | -1400 | -1700 | ^15,53^ |
| Kurukwade | Maharashtra | 21.32916667 | 74.63333333 | 2 | ass | Chalcolithic | Salvada | -2300 | -2000 | -2150 | ^28,29^ |
| Kurukwade | Maharashtra | 21.32916667 | 74.63333333 | 2 | ass | Chalcolithic | Malwa | -1700 | -1500 | -1600 | ^29^ |
| Kurukwade | Maharashtra | 21.32916667 | 74.63333333 | 2 | ass | Chalcolithic | Jorwe | -1500 | -1200 | -1350 | ^29^ |
| Kutwar | Madhya Pradesh | 24.984456 | 77.806614 | 3 | ass | Chalcolithic |  | -1400 | -1000 | -1200 | ^31^ |
| Labbarti | Andhra Pradesh | 17.56040336 | 82.2050508 | 2 | ass | Neolithic |  | -1700 | -1000 | -1350 | ^9,15^ |
| Lagori | Maharashtra | 18.6 | 74.55 | 2 | ass | Chalcolithic |  | -1700 | -1200 | -1450 | ^23^ |
| Lagori | Maharashtra | 18.6 | 74.55 | 2 | ass | Early Historic |  | -350 | 300 | -25 | ^23^ |
| Lakhangaon 1 | Karnataka | 18.171668 | 77.143236 | 3 | ass | Neolithic |  | -2200 | -1200 | -1700 | ^15,30^ |
| Lakhangaon 2 | Karnataka | 18.171999 | 77.14789 | 3 | ass | Neolithic |  | -2200 | -1200 | -1700 | ^15,30^ |
| Lakkavaram | Andhra Pradesh | 17.40316803 | 82.07625164 | 2 | ass | Neolithic |  | -1700 | -1000 | -1350 | ^9,15^ |
| Lakkundi | Karnataka | 15.388243 | 75.715263 | 2 | ass | Neolithic |  | -2200 | -1200 | -1700 | ^15^ |
| Lam | Andhra Pradesh | 16.378771 | 80.434585 | 3 | ass | Neolithic |  | -1700 | -1000 | -1350 | ^10,15,21^ |
| Lanjapoluru | Andhra Pradesh | 15.69970413 | 77.99388528 | 1 | ass | Neolithic |  | -2500 | -1200 | -1850 | ^15^ |
| Lanjawada | Karnataka | 18.12083333 | 77.15277778 | 3 | ass | Neolithic |  | -2200 | -1200 | -1700 | ^15,30^ |
| Lankalapalem | Andhra Pradesh | 17.59051225 | 83.06649968 | 2 | ass | Neolithic |  | -1700 | -1000 | -1350 | ^9,15^ |
| Lankapalli | Andhra Pradesh | 17.24425998 | 81.68483603 | 2 | ass | Neolithic |  | -1700 | -1000 | -1350 | ^9,15^ |
| Lattavaram | Andhra Pradesh | 14.96074493 | 77.31602113 | 1 | ass | Neolithic |  | -2500 | -1200 | -1850 | ^15^ |
| Lingadahalli | Karnataka | 15.52223755 | 77.03382018 | 1 | ass | Neolithic | Ashmound I/II | -2600 | -1600 | -2100 | ^15,35^ |
| Lingadhal | Karnataka | 15.581376 | 75.550688 | 2 | ass | Neolithic |  | -2200 | -1200 | -1700 | ^15^ |
| Lingamdinne | Andhra Pradesh | 15.08611111 | 78.28333333 | 2 | ass | Neolithic |  | -1700 | -1200 | -1450 | ^32^ |
| Lonkhede [a] | Maharashtra | 21.56666667 | 74.49166667 | 2 | ass | Chalcolithic | Malwa | -1700 | -1500 | -1600 | ^29^ |
| Lonkhede [b] | Maharashtra | 21.42333333 | 74.20333333 | 3 | ass | Chalcolithic | Malwa | -1700 | -1500 | -1600 | ^29^ |
| Lonkhedi | Maharashtra | 21.08333333 | 74.51666667 | 2 | ass | Chalcolithic | Kayatha | -2450 | -2000 | -2225 | ^28,29^ |
| Lonkhedi | Maharashtra | 21.08333333 | 74.51666667 | 2 | ass | Chalcolithic | Salvada | -2300 | -2000 | -2150 | ^28,29^ |
| Lonkhedi | Maharashtra | 21.08333333 | 74.51666667 | 2 | ass | Chalcolithic | Jorwe | -1500 | -1200 | -1350 | ^28,29^ |
| Lundopeta | Andhra Pradesh | 17.21749652 | 81.661418 | 2 | ass | Neolithic |  | -1700 | -1000 | -1350 | ^9,15^ |
| Madapur | Karnataka | 14.71636472 | 75.648999 | 1 | ass | Neolithic | IIB/III/IV/Jorwe | -2000 | -1200 | -1600 | ^6,15^ |
| Madhuravada | Andhra Pradesh | 17.80294722 | 83.2739165 | 2 | ass | Neolithic |  | -1700 | -1000 | -1350 | ^15^ |
| Magallu | Andhra Pradesh | 16.813129 | 80.316522 | 2 | ass | Neolithic |  | -1700 | -1000 | -1350 | ^10,15,21^ |
| Maheshwar | Madhya Pradesh | 22.170835 | 75.58934 | 3 | C14 | Chalcolithic | Malwa-type | -1700 | -1100 | -1400 | ^31^ |
| Mahidpur | Madhya Pradesh | 23.496581 | 75.660626 | 2 | C14 | Chalcolithic | Kaythata | -2450 | -1900 | -2175 | ^31^ |
| Mahidpur | Madhya Pradesh | 23.496581 | 75.660626 | 2 | ass | Chalcolithic | Malwa | -1900 | -1500 | -1700 | ^31^ |
| Mahidpur | Madhya Pradesh | 23.496581 | 75.660626 | 2 | ass | Chalcolithic |  | -1500 | -600 | -1050 | ^31^ |
| Malapuram | Andhra Pradesh | 15.09457219 | 77.20255889 | 1 | ass | Neolithic |  | -2500 | -1200 | -1850 | ^15,21^ |
| Malhar | Chhattisgarh | 22.09 | 82.13 | 1 | ass | Chalcolithic |  | -1000 | -300 | -650 | ^31^ |
| Mallappadi | Tamil Nadu | 12.528566 | 78.375732 | 1 | ass | Neolithic-Megalithic | | -1700 | -1000 | -1350 | ^15,25^ |
| Mallappadi | Tamil Nadu | 12.528566 | 78.375732 | 1 | ass | Megalithic |  | -1000 | -300 | -650 | ^15,25^ |
| Mallappakonda | Andhra Pradesh | 15.06257002 | 77.17928459 | 1 | ass | Neolithic |  | -2500 | -1200 | -1850 | ^15,36^ |
| Mallapuram | Andhra Pradesh | 15.81666667 | 77.81666667 | 3 | ass | Neolithic |  | -2500 | -1200 | -1850 | ^12^ |
| Malllipalli | Andhra Pradesh | 14.55635387 | 77.19674032 | 1 | ass | Neolithic |  | -2500 | -1200 | -1850 | ^15,35^ |
| Mallur | Karnataka | 16.46484693 | 76.47523685 | 1 | ass | Neolithic | Ashmound I/II | -2600 | -1600 | -2100 | ^15,35^ |
| Mamidilova | Andhra Pradesh | 17.841131 | 83.290377 | 2 | ass | Neolithic |  | -1700 | -1000 | -1350 | ^47^ |
| Manchanapalli | Andhra Pradesh | 16.11864163 | 77.55458276 | 1 | ass | Neolithic | Ashmound I/II | -2600 | -1600 | -2100 | ^35^ |
| Mandewal | Karnataka | 17.004327 | 76.546996 | 2 | ass | Neolithic |  | -2200 | -1200 | -1700 | ^15^ |
| Mandlem | Andhra Pradesh | 15.85972222 | 78.32916667 | 3 | ass | Neolithic |  | -1700 | -1200 | -1450 | ^32^ |
| Mandsaur | Madhya Pradesh | 24.075525 | 75.064299 | 2 | ass | Chalcolithic | Ahar | -2500 | -1700 | -2100 | ^31^ |
| Mangalam | Tamil Nadu | 11.80707654 | 79.78309752 | 1 | ass | Neolithic |  | -1700 | -1000 | -1350 | ^35^ |
| Manginipadu | Andhra Pradesh | 15.218534 | 79.897254 | 2 | ass | Neolithic |  | -1700 | -1000 | -1350 | ^43^ |
| Mannur | Karnataka | 16.790186 | 76.114895 | 2 | ass | Neolithic |  | -2200 | -1200 | -1700 | ^15^ |
| Manori | Maharashtra | 19.43544672 | 74.63190907 | 1 | ass | Chalcolithic | Jorwe | -1500 | -1200 | -1350 | ^27^ |
| Manoti | Madhya Pradesh | 24.422168 | 75.437359 | 2 | ass | Chalcolithic | Ahar/Malwa | -2100 | -1500 | -1800 | ^31^ |
| Manvi | Karnataka | 16.04009085 | 77.04545733 | 1 | ass | Neolithic | Ashmound I/II | -2600 | -1600 | -2100 | ^15,35^ |
| Marihal | Karnataka | 15.884759 | 74.670093 | 2 | ass | Neolithic |  | -2200 | -1200 | -1700 | ^15^ |
| Markavalasa | Andhra Pradesh | 17.83640155 | 83.27057107 | 2 | ass | Neolithic |  | -1700 | -1000 | -1350 | ^9,15^ |
| Marrivedu | Andhra Pradesh | 17.48847656 | 82.15988746 | 2 | ass | Neolithic |  | -1700 | -1000 | -1350 | ^9,15^ |
| Masali Khurd | Karnataka | 17.07990501 | 76.01142771 | 1 | ass | Chalcolithic | Jorwe | -1500 | -1200 | -1350 | ^27^ |
| Masimodu | Karnataka | 17.90416667 | 77.24166667 | 3 | ass | Neolithic |  | -2200 | -1200 | -1700 | ^15,30^ |
| Maski | Karnataka | 15.97608651 | 76.6352477 | 1 | ass | Neolithic |  | -2200 | -1200 | -1700 | ^15,35^ |
| Masur | Karnataka | 14.376257 | 75.459548 | 2 | ass | Neolithic |  | -2000 | -1000 | -1500 | ^6^ |
| Mattighatta | Karnataka | 13.94166667 | 75.69166667 | 2 | ass | Neolithic |  | -2000 | -1200 | -1600 | ^11^ |
| Mayaluru | Andhra Pradesh | 15.10277778 | 78.35555556 | 3 | ass | Neolithic |  | -1700 | -1200 | -1450 | ^32^ |
| Medad | Maharashtra | 18.2 | 74.55 | 2 | ass | Early Historic |  | -350 | 300 | -25 | ^23^ |
| Mekhali | Maharashtra | 18.049829 | 74.616909 | 3 | ass | Chalcolithic |  | -1700 | -1200 | -1450 | ^23^ |
| Mekhali | Maharashtra | 18.049829 | 74.616909 | 3 | ass | Early Historic |  | -350 | 300 | -25 | ^23^ |
| Methi | Maharashtra | 21.17916667 | 74.64166667 | 3 | ass | Chalcolithic | Late Harappan | -2000 | -1700 | -1850 | ^29^ |
| Metlapadu | Andhra Pradesh | 17.44498594 | 82.0511609 | 2 | ass | Neolithic |  | -1700 | -1000 | -1350 | ^9,15^ |
| Mettimel Kunda | Karnataka | 18.11111111 | 77.14027778 | 3 | ass | Neolithic |  | -2200 | -1200 | -1700 | ^15,30^ |
| Mettimel Kundwadi | Karnataka | 18.1125 | 77.0025 | 3 | ass | Neolithic |  | -2200 | -1200 | -1700 | ^15,30^ |
| Mettupalli | Andhra Pradesh | 15.23333333 | 78.1 | 2 | ass | Neolithic |  | -1700 | -1200 | -1450 | ^32^ |
| Minajgi | Karnataka | 17.226336 | 76.724648 | 2 | ass | Neolithic |  | -2200 | -1200 | -1700 | ^15^ |
| Mittasomapuram | Andhra Pradesh | 15.88333333 | 77.58333333 | 3 | ass | Neolithic |  | -2500 | -1200 | -1850 | ^12^ |
| Modur | Tamil Nadu | 12.222754 | 78.163669 | 2 | ass | Neolithic |  | -1700 | -1000 | -1350 | ^15,24^ |
| Muchehalapuri | Andhra Pradesh | 15.15 | 78.28333333 | 2 | ass | Neolithic |  | -1700 | -1200 | -1450 | ^32^ |
| Muchlamb | Karnataka | 17.95833333 | 77.175 | 3 | ass | Neolithic |  | -2200 | -1200 | -1700 | ^15,30^ |
| Mudavad | Maharashtra | 21.22333333 | 74.94027778 | 3 | ass | Chalcolithic | Malwa | -1700 | -1500 | -1600 | ^29^ |
| Mudavad | Maharashtra | 21.22333333 | 74.94027778 | 3 | ass | Early Historic |  | -350 | 300 | -25 | ^29^ |
| Mudenur | Karnataka | 14.491765 | 75.705599 | 3 | ass | Chalcolithic |  | -2000 | -1100 | -1550 | ^6,15^ |
| Mudhale | Maharashtra | 18.183735 | 74.39083 | 3 | ass | Early Historic |  | -350 | 300 | -25 | ^23^ |
| Mudigallu | Andhra Pradesh | 14.5811 | 77.17003 | 3 | ass | Neolithic |  | -2500 | -1200 | -1850 | ^15,21,35^ |
| Mukkamala | Andhra Pradesh | 15.15 | 78.25 | 2 | ass | Neolithic |  | -1700 | -1200 | -1450 | ^32^ |
| Mulakalura | Andhra Pradesh | 16.053023 | 79.748731 | 2 | ass | Neolithic |  | -1800 | -1200 | -1500 | ^49^ |
| Muppalla | Andhra Pradesh | 16.238949 | 79.845094 | 2 | ass | Neolithic |  | -1800 | -1200 | -1500 | ^21^ |
| Musalwadi | Maharashtra | 19.41090982 | 74.56102471 | 1 | ass | Chalcolithic | Jorwe | -1500 | -1200 | -1350 | ^27^ |
| Mushturu | Andhra Pradesh | 14.104166 | 78.175129 | 2 | ass | Neolithic |  | -2500 | -1200 | -1850 | ^21^ |
| Musurmalli | Andhra Pradesh | 17.35800469 | 81.80694432 | 2 | ass | Neolithic |  | -1700 | -1000 | -1350 | ^9,15^ |
| Muttur | Tamil Nadu | 11.794029 | 77.803015 | 2 | ass |  |  | -1700 | -1000 | -1350 | ^15^ |
| Mylavaram | Andhra Pradesh | 16.761486 | 80.646597 | 2 | ass | Neolithic |  | -1700 | -1000 | -1350 | ^15,21^ |
| Nadharalahalli | Karnataka | 14.565132 | 75.810047 | 2 | ass | Neolithic |  | -2200 | -1200 | -1700 | ^6,35^ |
| Nadiharhalli | Karnataka | 14.6098578 | 75.716985 | 1 | ass | Chalcolithic | Jorwe | -1500 | -900 | -1200 | ^27^ |
| Nadividi | Andhra Pradesh | 17.39647716 | 81.8052716 | 2 | ass | Neolithic |  | -1700 | -1000 | -1350 | ^9,15^ |
| Nagalapuram | Karnataka | 14.3032458 | 77.22292391 | 1 | ass | Neolithic | Ashmound I/II | -2600 | -1600 | -2100 | ^15^ |
| Nagaldinne | Andhra Pradesh | 15.91499146 | 77.56331063 | 1 | ass | Neolithic |  | -2500 | -1200 | -1850 | ^15^ |
| Nagampalli | Karnataka | 18.218317 | 77.562571 | 3 | ass | Neolithic |  | -2200 | -1200 | -1700 | ^15,30^ |
| Naganala | Karnataka | 13.193709 | 78.050502 | 2 | ass | Neolithic |  | -2200 | -1200 | -1700 | ^15^ |
| Nagarhalli | Karnataka | 17.27347383 | 76.09049103 | 1 | ass | Chalcolithic | Jorwe | -1500 | -1200 | -1350 | ^15,27^ |
| Nagarjunakonda | Andhra Pradesh | 16.46775621 | 79.28851852 | 1 | ass | Neolithic |  | -1700 | -1000 | -1350 | ^15,21^ |
| Nagarkudal | Tamil Nadu | 12.076452 | 78.053766 | 2 | ass |  |  | -1700 | -1000 | -1350 | ^15^ |
| Nagasamudra | Karnataka | 14.04166667 | 75.7 | 2 | ass | Neolithic |  | -2000 | -1200 | -1600 | ^11,15^ |
| Nagda | Madhya Pradesh | 23.455 | 75.40896 | 3 | ass | Chalcolithic |  | -2500 | -1700 | -2100 | ^31^ |
| Nagda | Madhya Pradesh | 23.455 | 75.40896 | 3 | ass | Ea Hist. |  | -300 | 300 | 0 | ^31^ |
| Nagoor (1) | Karnataka | 18.024749 | 77.372276 | 3 | ass | Neolithic |  | -2200 | -1200 | -1700 | ^15,30^ |
| Nagoor (2) | Karnataka | 18.02178 | 77.381973 | 3 | ass | Neolithic |  | -2200 | -1200 | -1700 | ^15,30^ |
| Nagur-Babli | Karnataka | 18.144485 | 77.395603 | 3 | ass | Neolithic |  | -2200 | -1200 | -1700 | ^15,30^ |
| Nakane | Maharashtra | 20.90833333 | 74.74166667 | 3 | ass | Chalcolithic | Malwa | -1700 | -1500 | -1600 | ^29^ |
| Nakane | Maharashtra | 20.90833333 | 74.74166667 | 3 | ass | Chalcolithic | Jorwe | -1500 | -1200 | -1350 | ^29^ |
| Nalave-Budruk | Maharashtra | 21.4 | 74.21666667 | 2 | ass | Chalcolithic | Malwa | -1700 | -1500 | -1600 | ^29^ |
| Nalave-Budruk | Maharashtra | 21.4 | 74.21666667 | 2 | ass | Chalcolithic | Jorwe | -1500 | -1200 | -1350 | ^29^ |
| Nallagonda | Andhra Pradesh | 14.87876 | 79.306787 | 1 | ass | Neolithic |  | -1700 | -1000 | -1350 | ^15,21^ |
| Nalwagal | Karnataka | 14.54987873 | 75.74697453 | 1 | ass | Chalcolithic | Jorwe | -1500 | -900 | -1200 | ^15,27^ |
| Namdapur | Karnataka | 17.871591 | 77.249274 | 3 | ass | Neolithic |  | -2200 | -1200 | -1700 | ^15,30^ |
| Nandurbar | Maharashtra | 21.36666667 | 74.23333333 | 2 | ass | Chalcolithic | Malwa | -1700 | -1500 | -1600 | ^29^ |
| Nandurbar | Maharashtra | 21.36666667 | 74.23333333 | 2 | ass | Chalcolithic | Jorwe | -1500 | -1200 | -1350 | ^29^ |
| Nangal | Maharashtra | 21.61258957 | 72.95649482 | 1 | ass | Chalcolithic | Jorwe | -1500 | -1200 | -1350 | ^27^ |
| Naramalapadu | Andhra Pradesh | 16.475708 | 79.884597 | 1 | ass | Neolithic |  | -2000 | -1200 | -1600 | ^10,15,21^ |
| Narapadu | Andhra Pradesh | 17.733775 | 83.039694 | 2 | ass | Neolithic |  | -1700 | -1000 | -1350 | ^15^ |
| Narava | Andhra Pradesh | 17.743798 | 83.181182 | 1 | ass | Neolithic |  | -1700 | -1000 | -1350 | ^21^ |
| Narsapur | Andhra Pradesh | 19.69 | 78.5 | 1 | ass | Neolithic |  | -1700 | -1000 | -1350 | ^15^ |
| Nasik | Maharashtra | 19.97525836 | 73.79765471 | 1 | ass | Chalcolithic | Jorwe | -1500 | -1200 | -1350 | ^27^ |
| Nasinde | Maharashtra | 21.45416667 | 74.39166667 | 3 | ass | Chalcolithic | Malwa | -1700 | -1500 | -1600 | ^29^ |
| Nasinde | Maharashtra | 21.45416667 | 74.39166667 | 3 | ass | Early Historic |  | -350 | 300 | -25 | ^29^ |
| Navadagi | Karnataka | 16.508282 | 76.413893 | 2 | ass | Neolithic |  | -2200 | -1200 | -1700 | ^15^ |
| Navdatoli | Madhya Pradesh | 22.163068 | 75.58468 | 3 | C14 | Chalcolithic |  | -1700 | -1200 | -1450 | ^54^ |
| Navdatoli | Madhya Pradesh | 22.163068 | 75.58468 | 3 | C14 | Chalcolithic | Jorwe | -1500 | -1100 | -1300 | ^54^ |
| Nawarkheri | Madhya Pradesh | 22.106017 | 75.212263 | 3 | ass | Chalcolithic |  | -2400 | -1200 | -1800 | ^19^ |
| Nepatvatan | Maharashtra | 18.197954 | 74.517506 | 2 | ass | Chalcolithic |  | -1700 | -1200 | -1450 | ^23^ |
| Nepatvatan | Maharashtra | 18.197954 | 74.517506 | 2 | ass | Early Historic |  | -350 | 300 | -25 | ^23^ |
| Neravada | Andhra Pradesh | 15.79166667 | 77.96805556 | 3 | ass | Neolithic |  | -2500 | -1200 | -1850 | ^12^ |
| Nevasa | Maharashtra | 19.547247 | 74.917681 | 3 | C14 | Chalcolithic | Malwa-Jorwe | -1700 | -1200 | -1450 | ^27^ |
| Nevasa | Maharashtra | 19.547247 | 74.917681 | 3 | ass | Early Historic |  | -150 | 200 | 25 | ^27^ |
| Nichchenametla | Andhra Pradesh | 15.21666667 | 78.13333333 | 2 | ass | Neolithic |  | -1700 | -1200 | -1450 | ^32^ |
| Nidagundi | Karnataka | 16.486931 | 74.845905 | 2 | ass | Neolithic |  | -2200 | -1200 | -1700 | ^15^ |
| Nidzuru | Andhra Pradesh | 15.86666667 | 77.98333333 | 3 | ass | Neolithic |  | -2500 | -1200 | -1850 | ^12^ |
| Nilaskal | Karnataka | 13.7875 | 75.5 | 2 | ass | Neolithic |  | -2000 | -1200 | -1600 | ^11^ |
| Nilunagondla | Andhra Pradesh | 15.24722222 | 78.24861111 | 3 | ass | Neolithic |  | -1700 | -1200 | -1450 | ^32^ |
| Nimbapur | Karnataka | 15.42332175 | 76.74580065 | 1 | ass | Neolithic | Ashmound I/II | -2600 | -1600 | -2100 | ^15^ |
| Nimbegondi | Karnataka | 14.08333333 | 75.73333333 | 2 | ass | Neolithic |  | -1800 | -800 | -1300 | ^11^ |
| Nimbhel | Maharashtra | 21.38 | 74.41388889 | 3 | ass | Chalcolithic | Malwa-Jorwe | -1700 | -1200 | -1450 | ^29^ |
| Nipania | Madhya Pradesh | 23.645595 | 76.345007 | 2 | ass | Chalcolithic | Kayatha | -2450 | -1900 | -2175 | ^47^ |
| Nipania | Madhya Pradesh | 23.645595 | 76.345007 | 2 | ass | Chalcolithic | Malwa-type | -1900 | -1500 | -1700 | ^47^ |
| Niralgi | Karnataka | 14.94355 | 75.649104 | 2 | ass | Neolithic |  | -2000 | -1200 | -1600 | ^6,35^ |
| Nishane | Maharashtra | 21.18333333 | 74.71666667 | 2 | ass | Chalcolithic | Malwa | -1700 | -1500 | -1600 | ^29^ |
| Nishane | Maharashtra | 21.18333333 | 74.71666667 | 2 | ass | Early Historic |  | -350 | 300 | -25 | ^29^ |
| Nittur | Karnataka | 18.11655 | 77.354168 | 3 | ass | Neolithic |  | -2200 | -1200 | -1700 | ^15,30^ |
| Nyahli | Maharashtra | 21.3375 | 74.46333333 | 3 | ass | Chalcolithic | Jorwe | -1500 | -1200 | -1350 | ^29^ |
| Nyahli | Maharashtra | 21.3375 | 74.46333333 | 3 | ass | Early Historic |  | -350 | 300 | -25 | ^29^ |
| Nyamti | Karnataka | 14.1625 | 75.59166667 | 2 | ass | Neolithic |  | -2000 | -1200 | -1600 | ^11^ |
| Orathi | Tamil Nadu | 12.379635 | 79.686786 | 2 | ass | Neolithic |  | -1700 | -1000 | -1350 | ^15^ |
| Osrel | Maharashtra | 21.43333333 | 74.46666667 | 2 | ass | Chalcolithic | Jorwe | -1500 | -1200 | -1350 | ^29^ |
| Owk | Andhra Pradesh | 15.06666667 | 78 | 2 | ass | Neolithic |  | -1700 | -1200 | -1450 | ^32^ |
| Padeknur | Karnataka | 16.494895 | 76.135936 | 2 | ass | Neolithic |  | -2200 | -1200 | -1700 | ^15^ |
| Padipalli | Andhra Pradesh | 18.209398 | 79.706986 | 2 | ass | Neolithic |  | -2000 | -1000 | -1500 | ^15^ |
| Paithan | Maharashtra | 19.463482 | 75.382385 | 3 | ass | Chalcolithic | Jorwe | -1500 | -1200 | -1350 | ^29^ |
| Paithan | Maharashtra | 19.463482 | 75.382385 | 3 | C14 | Early Historic | Paithan 1 | -350 | 250 | -50 | ^27^ |
| Paiyampalli | Tamil Nadu | 12.549968 | 78.432852 | 2 | C14 | Chalcolithic |  | -2000 | -1450 | -1725 | ^15^ |
| Palapadu | Andhra Pradesh | 16.248089 | 79.976389 | 2 | ass | Neolithic |  | -1700 | -1000 | -1350 | ^10,15,21^ |
| Palavoy | Andhra Pradesh | 14.50398669 | 77.1763753 | 1 | C14 | Neolithic |  | -2350 | -1600 | -1975 | ^15,36,53^ |
| Pallagiri | Andhra Pradesh | 16.780338 | 80.326239 | 2 | ass | Neolithic |  | -1700 | -1000 | -1350 | ^15,21^ |
| Pandipadu | Andhra Pradesh | 15.75138889 | 78 | 3 | ass | Neolithic |  | -2500 | -1200 | -1850 | ^12,32,35^ |
| Pangir | Andhra Pradesh | 19.69 | 78.5 | 1 | ass | Neolithic |  | -1700 | -1000 | -1350 | ^15^ |
| Pannagaram | Tamil Nadu | 12.129603 | 77.894185 | 2 | ass | Neolithic |  | -1700 | -1000 | -1350 | ^15^ |
| Paparajanahalli | Karnataka | 13.13439 | 78.091633 | 2 | ass | Neolithic |  | -2200 | -1200 | -1700 | ^15^ |
| Paradesipalem | Andhra Pradesh | 17.856483 | 83.368741 | 2 | ass | Neolithic |  | -1700 | -1000 | -1350 | ^15^ |
| Paramata Singavaram | Andhra Pradesh | 15.86805556 | 77.78611111 | 3 | ass | Neolithic |  | -2500 | -1200 | -1850 | ^12^ |
| Parasamal I | Maharashtra | 21.246856 | 74.737923 | 3 | ass | Chalcolithic | Malwa | -1700 | -1500 | -1600 | ^29^ |
| Parasamal II | Maharashtra | 21.24166667 | 74.73601 | 3 | ass | Chalcolithic | Malwa | -1700 | -1500 | -1600 | ^29^ |
| Parasamal III | Maharashtra | 21.247154 | 74.740965 | 3 | ass | Chalcolithic | Malwa | -1700 | -1500 | -1600 | ^29^ |
| Pareshwar | Andhra Pradesh | 19.69 | 78.5 | 1 | ass | Neolithic |  | -1700 | -1000 | -1350 | ^15^ |
| Pargaon | Maharashtra | 21.89583333 | 74.10833333 | 3 | ass | Chalcolithic | Jorwe | -1500 | -1200 | -1350 | ^29^ |
| Pargaon | Maharashtra | 21.89583333 | 74.10833333 | 3 | ass | Early Historic |  | -350 | 300 | -25 | ^29^ |
| Patan | Maharashtra | 21.27666667 | 74.75833333 | 2 | ass | Chalcolithic | Late Harappan | -2000 | -1700 | -1850 | ^29^ |
| Pathre Khurd | Maharashtra | 19.47906786 | 74.78185675 | 1 | ass | Chalcolithic | Jorwe | -1500 | -1200 | -1350 | ^27^ |
| Pattikonda | Andhra Pradesh | 15.39131958 | 77.50221558 | 1 | ass | Neolithic |  | -2500 | -1200 | -1850 | ^35^ |
| Patupadu | Andhra Pradesh | 15.32777778 | 78.15555556 | 2 | ass | Neolithic |  | -2000 | -1200 | -1600 | ^15^ |
| Payagod Hill | Karnataka | 13.25590205 | 75.90501636 | 1 | ass | Neolithic |  | -2500 | -1200 | -1850 | ^15^ |
| Peddabankuru | Andhra Pradesh | 18.378 | 79.1 | 1 | ass | Neolithic |  | -2000 | -1000 | -1500 | ^15^ |
| Peddadandukonda | Andhra Pradesh | 14.82691768 | 77.65931714 | 1 | ass | Neolithic |  | -2500 | -1200 | -1850 | ^35^ |
| Peddakopperla | Andhra Pradesh | 15.3 | 78.32777778 | 2 | ass | Neolithic |  | -1700 | -1200 | -1450 | ^32^ |
| Peddammanuru | Andhra Pradesh | 15.08 | 78.43333333 | 2 | ass | Neolithic |  | -1700 | -1200 | -1450 | ^32^ |
| Peddamudiyam (2) | Andhra Pradesh | 15.013967 | 78.448833 | 3 | ass | Neothic (1700-1400 BC) | | -1700 | -1400 | -1550 | ^33,53^ |
| Peetnagar | Madhya Pradesh | 22.184277 | 75.957716 | 3 | ass | Chalcolithic | Ahar | -2500 | -1700 | -2100 | ^31^ |
| Peetnagar | Madhya Pradesh | 22.184277 | 75.957716 | 3 | ass | Chalcolithic | Malwa-type | -1700 | -1200 | -1450 | ^31^ |
| Penchikalapadu | Andhra Pradesh | 15.75138889 | 77.89583333 | 3 | ass | Neolithic |  | -2000 | -1200 | -1600 | ^15^ |
| Periyatanda | Tamil Nadu | 11.660115 | 78.146372 | 1 | ass |  |  | -1700 | -1000 | -1350 | ^15^ |
| Perumanallur | Tamil Nadu | 11.210628 | 77.360537 | 2 | ass |  |  | -1700 | -1000 | -1350 | ^15^ |
| Phootipal | Madhya Pradesh | 23.327772 | 76.007173 | 2 | ass | Chalcolithic |  | -2450 | -1500 | -1975 | ^34^ |
| Piklihal IIIA | Karnataka | 15.979285 | 76.444632 | 3 | AMS | Southern Neolithic |  | -1800 | -1400 | -1600 | ^15^ |
| Piklihal IIIB | Karnataka | 15.979285 | 76.444632 | 3 | AMS | Southern Neolithic |  | -1400 | -800 | -1100 | ^15^ |
| Pilangere | Karnataka | 13.95833333 | 75.63333333 | 2 | ass | Neolithic | IIA | -2100 | -1700 | -1900 | ^11^ |
| Pillalapalli | Andhra Pradesh | 14.58253747 | 77.06291306 | 1 | ass | Neolithic |  | -2500 | -1200 | -1850 | ^15^ |
| Pimpalgaon | Maharashtra | 19.53086797 | 74.60737218 | 1 | ass | Chalcolithic | Jorwe | -1500 | -1200 | -1350 | ^27^ |
| Pimpalsuti | Maharashtra | 18.612313 | 74.514147 | 2 | ass | Chalcolithic-Iron Age | Jorwe-Iron Age | -1500 | -500 | -1000 | ^22,23^ |
| Pimplas [=Pimlas] | Gujarat | 21.52916667 | 74.17083333 | 3 | ass | Chalcolithic | Salvada | -2300 | -2000 | -2150 | ^27,28^ |
| Pimplod | Maharashtra | 21.643613 | 74.401555 | 3 | ass | Chalcolithic | Jorwe | -1500 | -1200 | -1350 | ^27^ |
| Pimpri | Maharashtra | 21.17916667 | 74.68333333 | 2 | ass | Chalcolithic | Malwa | -1700 | -1500 | -1600 | ^29^ |
| Pimprivalan | Maharashtra | 19.40273086 | 74.70006711 | 1 | ass | Chalcolithic | Jorwe | -1500 | -1200 | -1350 | ^27^ |
| Pipilya Lorka | Madhya Pradesh | 23.094415 | 77.560022 | 2 | ass | Chalcolithic | Kayatha | -2450 | -2000 | -2225 | ^49^ |
| Pipri | Madhya Pradesh | 22.067155 | 74.989608 | 3 | ass | Chalcolithic |  | -2450 | -1500 | -1975 | ^19^ |
| Polakonda | Andhra Pradesh | 17.704549 | 79.14948 | 1 | ass | Neolithic |  | -2000 | -1000 | -1500 | ^15,21^ |
| Polavaram | Andhra Pradesh | 17.43996779 | 81.88221655 | 2 | ass | Neolithic |  | -1700 | -1000 | -1350 | ^9,15^ |
| Ponakaladinne | Andhra Pradesh | 15.84166667 | 77.6 | 3 | ass | Neolithic |  | -2000 | -1200 | -1600 | ^12^ |
| Ponnekallu | Andhra Pradesh | 15.78611111 | 77.77916667 | 3 | ass | Neolithic |  | -2000 | -1200 | -1600 | ^12^ |
| Ponnekallu | Andhra Pradesh | 16.407154 | 80.398676 | 3 | ass | Neolithic |  | -1700 | -1000 | -1350 | ^10,15^ |
| Prakash II [=Ramat Barada] | Maharashtra | 21.5157 | 74.334507 | 3 | ass | Chalcolithic | Jorwe | -1500 | -1200 | -1350 | ^29^ |
| Prakashe I | Maharashtra | 21.508036 | 74.358638 | 3 | ass | Chalcolithic | Late Harappan | -2000 | -1700 | -1850 | ^29^ |
| Prakashe I | Maharashtra | 21.508036 | 74.358638 | 3 | C14 | Chalcolithic | Malwa | -1700 | -1500 | -1600 | ^38^ |
| Prakashe I | Maharashtra | 21.508036 | 74.358638 | 3 | ass | Chalcolithic | Jorwe | -1500 | -1200 | -1350 | ^29^ |
| Pravar Sangam | Maharashtra | 19.61538393 | 74.99996246 | 1 | ass | Chalcolithic | Jorwe | -1500 | -1200 | -1350 | ^29^ |
| Pusalapadu | Andhra Pradesh | 15.42332175 | 79.10814266 | 1 | ass | Neolitihic |  | -1700 | -1000 | -1350 | ^15^ |
| Pushpattur | Tamil Nadu | 10.525417 | 77.422872 | 2 | ass |  |  | -1700 | -1000 | -1350 | ^15^ |
| Raipalli | Karnataka | 18.255368 | 77.559091 | 3 | ass | Neolithic |  | -2200 | -1200 | -1700 | ^15,30^ |
| Rajankolur | Karnataka | 16.376712 | 76.459466 | 2 | ass | Neolithic | Ashmound I/II | -2600 | -1600 | -2100 | ^15,35^ |
| Ramadurga Hill | Karnataka | 15.28076663 | 77.10946167 | 1 | ass | Neolithic |  | -2500 | -1200 | -1850 | ^15^ |
| Ramapuram | Andhra Pradesh | 15.083333 | 78.083333 | 2 | C14 | Neolitihic |  | -1800 | -1200 | -1500 | ^53,55^. |
| Ramatirtha | Karnataka | 14.296888 | 74.448527 | 2 | ass | Neolithic |  | -2200 | -1000 | -1600 | ^15,44^ |
| Rampachoda-varam | Andhra Pradesh | 17.4717494 | 81.8454168 | 2 | ass | Neolithic |  | -1700 | -1000 | -1350 | ^9,15^ |
| Rangai | Madhya Pradesh | 23.503 | 77.782952 | 3 | ass | Chalcolithic |  | -2400 | -1200 | -1800 | ^31^ |
| Ranjale | Maharashtra | 21.28333333 | 21.75 | 2 | ass | Chalcolithic | Jorwe | -1500 | -1200 | -1350 | ^29^ |
| Ranjale | Maharashtra | 21.28333333 | 21.75 | 2 | ass | Early Historic |  | -350 | 300 | -25 | ^29^ |
| Ravivalasa | Andhra Pradesh | 18.45530657 | 84.10860193 | 2 | ass | Neolithic |  | -1700 | -1000 | -1350 | ^9,15^ |
| Rayadrug | Andhra Pradesh | 14.7221833 | 76.8796279 | 1 | ass | Neolithic |  | -2500 | -1000 | -1750 | ^15,36^ |
| Reddinagalpalem | Andhra Pradesh | 17.18738763 | 81.68650875 | 2 | ass | Neolithic |  | -1700 | -1000 | -1350 | ^9,15^ |
| Remaduru | Andhra Pradesh | 15.66666667 | 77.91388889 | 3 | ass | Neolithic |  | -2000 | -1200 | -1600 | ^12^ |
| Remata-Northeast | Andhra Pradesh | 65.04166667 | 77.8625 | 3 | ass | Neolithic |  | -2000 | -1200 | -1600 | ^12^ |
| Remata-South | Andhra Pradesh | 15.825 | 77.85326087 | 3 | ass | Neolithic |  | -2000 | -1200 | -1600 | ^12^ |
| Rodalkunda | Karnataka | 15.72588773 | 76.88253719 | 1 | ass | Neolithic |  | -2200 | -1000 | -1600 | ^15^ |
| Rodgi | Karnataka | 17.19168418 | 76.21862814 | 1 | ass | Chalcolithic | Jorwe | -1500 | -1200 | -1350 | ^27^ |
| Rui | Maharashtra | 18.16666667 | 74.51666667 | 2 | ass | Early Historic |  | -350 | 300 | -25 | ^23^ |
| Runija | Madhya Pradesh | 23.155126 | 75.262163 | 3 | ass | Chalcolithic | Kayatha | -2450 | -2000 | -2225 | ^31^ |
| Runija | Madhya Pradesh | 23.155126 | 75.262163 | 3 | ass | Chalcolithic | Ahar | -2100 | -1700 | -1900 | ^31^ |
| Runija | Madhya Pradesh | 23.155126 | 75.262163 | 3 | ass | Chalcolithic | Malwa | -1800 | -1400 | -1600 | ^31^ |
| Runija | Madhya Pradesh | 23.155126 | 75.262163 | 3 | ass | Sunga Period (185-73 BC) | | -185 | -73 | -129 | ^31^ |
| Rupabhada | Andhra Pradesh | 18.72628661 | 84.03834784 | 2 | ass | Neolithic |  | -1700 | -1000 | -1350 | ^9,15^ |
| Rupanagudi | Andhra Pradesh | 15.098262 | 78.372746 | 3 | C14 | Neolithic |  | -1700 | -1400 | -1550 | ^15,53^ |
| Sadalga | Karnataka | 16.51139554 | 74.48819301 | 1 | ass | Neolithic | Ashmound I/II | -2600 | -1600 | -2100 | ^15^ |
| Saitana | Maharashtra | 21.27083333 | 74.42361111 | 2 | ass | Chalcolithic | Jorwe | -1500 | -1200 | -1350 | ^27^ |
| Saitana | Maharashtra | 21.27083333 | 74.42361111 | 2 | ass | Chalcolithic |  | -350 | 300 | -25 | ^27^ |
| Sakri | Maharashtra | 20.99027778 | 74.31666667 | 2 | ass | Chalcolithic | Jorwe | -1500 | -1200 | -1350 | ^27,29^ |
| Sakshal Pimpri | Maharashtra | 19.100877 | 75.602101 | 3 | C14 | Neo/Meso |  | -5800 | -2000 | -3900 | ^19^ |
| Sambowaza-Mal | Maharashtra | 18.16666667 | 74.88333333 | 2 | ass | Early Historic |  | -350 | 300 | -25 | ^29^ |
| Sanavaspur | Karnataka | 15.4378682 | 76.96690655 | 1 | ass | Neolithic | Ashmound I/II | -2600 | -1600 | -2100 | ^15^ |
| Sangamedu | Tamil Nadu | 12.015991 | 79.299409 | 2 | ass |  |  | -1700 | -1000 | -1350 | ^15^ |
| Sangamner | Maharashtra | 19.51178372 | 74.16843443 | 1 | ass | Chalcolithic | Jorwe | -1500 | -1200 | -1350 | ^27^ |
| Sanganakallu | Karnataka | 15.18009 | 76.961889 | 3 | AMS | Neolithic | Ashmound IIB-IIB | -2000 | -1300 | -1650 | ^15,26,46,53^ |
| Sangannal | Karnataka | 18.288953 | 77.297084 | 3 | ass | Neolithic |  | -2200 | -1200 | -1700 | ^15,30^ |
| Sangavi | Maharashtra | 18.058964 | 74.481709 | 3 | ass | Chalcolithic |  | -1700 | -1200 | -1450 | ^23^ |
| Sangnal | Karnataka | 18.050822 | 77.4098 | 3 | ass | Neolithic |  | -2200 | -1200 | -1700 | ^15,30^ |
| Sangwar | Karnataka | 16.523576 | 77.256762 | 2 | ass | Neolithic |  | -2200 | -1200 | -1700 | ^15^ |
| Sanjamala | Andhra Pradesh | 15.15138889 | 78.28333333 | 2 | ass | Neolithic |  | -1700 | -1200 | -1450 | ^32^ |
| Sanjamala East | Andhra Pradesh | 15.15138889 | 78.28333333 | 2 | ass | Neolithic |  | -1700 | -1200 | -1450 | ^32^ |
| Santamagaluru | Andhra Pradesh | 16.133033 | 79.957939 | 2 | ass | Neolithic |  | -1700 | -1000 | -1350 | ^21^ |
| Sarangkheda | Maharashtra | 21.430539 | 74.529781 | 2 | ass | Chalcolithic | Salvada | -2300 | -2000 | -2150 | ^28^ |
| Saravanipalem | Andhra Pradesh | 17.672533 | 82.996772 | 1 | ass | Neolithic |  | -1700 | -1000 | -1350 | ^15^ |
| Sashtewadi | Maharashtra | 18.528795 | 74.054104 | 3 | ass | Chalcolithic | Malwa-Jorwe | -1700 | -1200 | -1450 | ^22^ |
| Sasivehalli | Karnataka | 14.15 | 75.70833333 | 2 | ass | Neolithic |  | -2100 | -1700 | -1900 | ^11^ |
| Satola | Gujarat | 21.51389 | 74.265065 | 2 | ass | Chalcolithic | Salvada | -2300 | -2000 | -2150 | ^28^ |
| Satral | Maharashtra | 19.45998361 | 74.41107703 | 1 | ass | Chalcolithic | Jorwe | -1500 | -1200 | -1350 | ^27^ |
| Satti | Karnataka | 16.59867418 | 75.05550421 | 1 | ass | Neolithic |  | -2500 | -1200 | -1850 | ^35^ |
| Savadi | Karnataka | 16.586017 | 75.15487 | 2 | ass | Neolithic |  | -2500 | -1800 | -2150 | ^15^ |
| Savalda | Gujarat | 21.511524 | 74.325986 | 3 | ass | Chalcolithic | Salvada | -2300 | -2000 | -2150 | ^28,29^ |
| Senagapadu | Andhra Pradesh | 16.772858 | 80.293124 | 1 | ass | Neolithic |  | -1700 | -1000 | -1350 | ^10,15,21^ |
| Senapatipakalu | Andhra Pradesh | 17.34127753 | 82.07457893 | 2 | ass | Neolithic |  | -1700 | -1000 | -1350 | ^9,15^ |
| Shahada | Maharashtra | 21.54820908 | 74.52308697 | 1 | ass | Chalcolithic | Jorwe | -1500 | -1200 | -1350 | ^27^ |
| Shakapur | Karnataka | 16.80523364 | 76.72543563 | 1 | ass | Neolithic | Ashmound I/II | -2600 | -1600 | -2100 | ^15,35^ |
| Shegunashi | Karnataka | 16.584895 | 75.582132 | 2 | ass | Neolithic |  | -2200 | -1200 | -1700 | ^15^ |
| Shemballi | Karnataka | 18.133405 | 77.473702 | 3 | ass | Neolithic |  | -2200 | -1200 | -1700 | ^15,30^ |
| Sheriwadi | Maharashtra | 18.632531 | 74.103776 | 3 | ass | Chalcolithic | Late Jorwe | -1200 | -800 | -1000 | ^22^ |
| Shettihalli | Karnataka | 12.67986299 | 77.09491523 | 1 | ass | Neolithic |  | -2200 | -1200 | -1700 | ^15^ |
| Shirguppi | Karnataka | 16.52303269 | 75.19224076 | 1 | ass | Neolithic |  | -2200 | -1200 | -1700 | ^15,35^ |
| Shirol | Karnataka | 16.393137 | 75.267172 | 2 | ass | Neolithic |  | -2200 | -1200 | -1700 | ^15,44^ |
| Shivani | Karnataka | 18.150925 | 77.08959 | 3 | ass | Neolithic |  | -2200 | -1200 | -1700 | ^15,30^ |
| Shivapura | Karnataka | 15.351434 | 76.476446 | 2 | ass | Neolithic |  | -2000 | -1000 | -1500 | ^20^ |
| Shivapura | Karnataka | 15.351434 | 76.476446 | 2 | ass | Iron Age |  | -1000 | -300 | -650 | ^14,20^ |
| Singanapalli | Andhra Pradesh | 1.363636364 | 78.15 | 2 | ass | Neolithic |  | -2000 | -1200 | -1600 | ^15,32^ |
| Sircarasamakkulam | Tamil Nadu | 11.135102 | 77.029995 | 2 | ass |  |  | -1700 | -1000 | -1350 | ^15^ |
| Sirpur | Andhra Pradesh | 18.49879719 | 83.89449424 | 2 | ass | Neolithic |  | -1700 | -1000 | -1350 | ^9,15^ |
| Sirsi | Karnataka | 17.815408 | 77.362936 | 2 | ass | Neolithic |  | -2200 | -1200 | -1700 | ^15,30^ |
| Sitapalli | Andhra Pradesh | 17.38811358 | 81.83705321 | 2 | ass | Neolithic |  | -1700 | -1000 | -1350 | ^9,15^ |
| Sivapuram | Andhra Pradesh | 15.790913 | 79.239661 | 2 | ass | Neolithic |  | -1700 | -1000 | -1350 | ^21^ |
| Sivarakottai | Tamil Nadu | 9.741608 | 77.984639 | 1 | ass |  |  | -1700 | -1000 | -1350 | ^15^ |
| Sivavaram | Andhra Pradesh | 15.20833333 | 78.18333333 | 3 | ass | Neolithic |  | -1700 | -1200 | -1450 | ^15,32^ |
| Sonagadh | Andhra Pradesh | 19.69 | 78.5 | 1 | ass | Neolithic |  | -1700 | -1000 | -1350 | ^15^ |
| Sonegaon | Maharashtra | 18.071227 | 74.656652 | 3 | C14 | Chalcolithic | Malwa | -1700 | -1500 | -1600 | ^22^ |
| Sonegaon | Maharashtra | 18.071227 | 74.656652 | 3 | C14 | Chalcolithic | Jorwe | -1500 | -900 | -1200 | ^22,23^ |
| Souralli | Karnataka | 18.087834 | 77.533665 | 3 | ass | Neolithic |  | -2200 | -1200 | -1700 | ^15,30^ |
| Sriwavasupur | Karnataka | 13.25881134 | 78.18880759 | 1 | ass | Neolithic |  | -2200 | -1200 | -1700 | ^35^ |
| Suguru | Karnataka | 15.49314467 | 76.79816784 | 1 | ass | Neolithic | Ashmound I/II | -2600 | -1600 | -2100 | ^15^ |
| Suguru | Andhra Pradesh | 15.81666667 | 77.51805556 | 3 | ass | Neolithic |  | -2000 | -1200 | -1600 | ^12^ |
| Sulebailu | Karnataka | 14.56666667 | 75.9 | 2 | ass | Neolithic | IIB | -1800 | -1200 | -1500 | ^11,15^ |
| Sulikunte | Karnataka | 13.025517 | 78.111868 | 2 | ass | Neolithic |  | -2200 | -1200 | -1700 | ^15^ |
| Sulwade | Maharashtra | 21.29166667 | 74.78333333 | 2 | ass | Chalcolithic | Malwa | -1700 | -1500 | -1600 | ^29^ |
| Sulwade | Maharashtra | 21.29166667 | 74.78333333 | 2 | ass | Early Historic |  | -350 | 300 | -25 | ^29^ |
| Surkod | Karnataka | 15.686611 | 75.471185 | 2 | ass | Neolithic |  | -2200 | -1200 | -1700 | ^15^ |
| Surpan | Maharashtra | 21.01666667 | 74.21666667 | 2 | ass | Chalcolithic | Late Harappan | -2000 | -1700 | -1850 | ^29^ |
| Surpan | Maharashtra | 21.01666667 | 74.21666667 | 2 | ass | Early Historic |  | -350 | 300 | -25 | ^29^ |
| T. Kallupatti | Tamil Nadu | 9.7184 | 77.85354 | 2 | ass | Neolithic |  | -1700 | -1000 | -1350 | ^15^ |
| T. Narsipur | Karnataka | 12.29001838 | 76.87090004 | 1 | C14 | Neolithic |  | -2200 | -1000 | -1700 | ^15^ |
| Tadipatri | Andhra Pradesh | 14.906552 | 78.009736 | 2 | ass | Neolithic |  | -2500 | -1200 | -1850 | ^15^ |
| Tallihalli | Karnataka | 14.939301 | 75.381081 | 2 | ass | Neolithic |  | -2200 | -1200 | -1700 | ^15^ |
| Talmari | Andhra Pradesh | 16.00517939 | 77.53421775 | 1 | ass | Neolithic | Ashmound I/II | -2600 | -1600 | -2100 | ^15^ |
| Tamasvadi | Maharashtra | 20.95833333 | 74.40833333 | 3 | ass | Chalcolithic | Jorwe | -1500 | -1200 | -1350 | ^29^ |
| Tamasvadi | Maharashtra | 20.95833333 | 74.40833333 | 3 | ass | Early Historic |  | -350 | 300 | -25 | ^29^ |
| Tamdallapalle | Andhra Pradesh | 13.821088 | 78.446728 | 2 | ass | Neolithic |  | -2000 | -1200 | -1600 | ^15,32^ |
| Tanakal | Andhra Pradesh | 13.916114 | 78.192841 | 2 | ass | Neolithic |  | -2500 | -1200 | -1850 | ^15^ |
| Tandali | Maharashtra | 18.549354 | 74.575531 | 3 | ass | Early Historic |  | -350 | 300 | -25 | ^23^ |
| Tangadancha | Andhra Pradesh | 15.85833333 | 78.35416667 | 3 | ass | Neolithic |  | -1700 | -1200 | -1450 | ^32^ |
| Tanguturu | Andhra Pradesh | 15.35 | 78.35 | 2 | ass | Neolithic |  | -1700 | -1200 | -1450 | ^32^ |
| Tarakadu | Tamil Nadu | 11.660115 | 78.146372 | 1 | ass | Neolithic |  | -1700 | -1000 | -1350 | ^15^ |
| Tardoli | Maharashtra | 18.262936 | 74.344504 | 3 | ass | Chalcolithic |  | -1700 | -1200 | -1450 | ^23^ |
| Tarlapadu | Andhra Pradesh | 15.65578 | 79.224303 | 2 | ass | Neolithic |  | -1700 | -1000 | -1350 | ^21^ |
| Tarturu | Andhra Pradesh | 15.84722222 | 78.33055556 | 3 | ass | Neolithic |  | -1700 | -1200 | -1450 | ^32^ |
| Tavarager | Karnataka | 15.765923 | 76.407234 | 2 | ass | Neolithic | Ashmound I/II | -2600 | -1600 | -2100 | ^15^ |
| Tavaragera | Karnataka | 15.79571064 | 76.46069041 | 1 | ass | Neolithic |  | -2500 | -1200 | -1850 | ^15^ |
| Tegampur | Karnataka | 18.243195 | 77.467177 | 3 | ass | Neolithic |  | -2200 | -1200 | -1700 | ^15,30^ |
| Tekal | Karnataka | 12.985925 | 78.073256 | 2 | ass | Neolithic |  | -2200 | -1200 | -1700 | ^15^ |
| Tekkalakota | Karnataka | 15.52041 | 76.869302 | 3 | AMS | Neolithic |  | -1700 | -1400 | -1550 | ^15,53^ |
| Tekwad | Maharashtra | 20.53600456 | 75.2026589 | 1 | ass | Chalcolithic | Jorwe | -1500 | -1200 | -1350 | ^27^ |
| Tekwada | Maharashtra | 21.369276 | 74.768057 | 3 | ass | Chalcolithic | Jorwe | -1500 | -1100 | -1300 | ^56^ |
| Tellapadu | Andhra Pradesh | 15.6634 | 79.948877 | 2 | ass | Neolithic |  | -1700 | -1000 | -1350 | ^21^ |
| Terdal | Karnataka | 16.45902835 | 75.03222991 | 1 | C14 | Neolithic |  | -2300 | -1900 | -2100 | ^15,53^ |
| Thalner | Maharashtra | 21.25 | 74.95 | 2 | ass | Chalcolithic | Jorwe | -1500 | -1200 | -1350 | ^27,28^ |
| Thameshwansa | Maharashtra | 20.018087 | 79.74393 | 1 | ass | Chalcolithic |  | -2000 | -1500 | -1750 | ^45^ |
| Thanmandi Thanda | Karnataka | 16.43866333 | 76.55669692 | 1 | ass | Neolithic | Ashmound I/II | -2600 | -1600 | -2100 | ^15,35^ |
| Theur | Maharashtra | 18.519583 | 74.058417 | 3 | ass | Chalcolithic | Late Jorwe | -1200 | -800 | -1000 | ^22^ |
| Thogarrai | Andhra Pradesh | 16.927093 | 79.950889 | 3 | ass | Neolithic | Phase I | -2500 | -1800 | -2150 | ^21^ |
| Tilali | Maharashtra | 21.27916667 | 74.35 | 2 | ass | Chalcolithic | Jorwe | -1500 | -1200 | -1350 | ^29^ |
| Tilapur | Maharashtra | 19.53632061 | 74.79821468 | 1 | ass | Chalcolithic | Jorwe | -1500 | -1200 | -1350 | ^27^ |
| Tirth | Karnataka | 16.40375187 | 76.486874 | 1 | ass | Neolithic | Ashmound I/II | -2600 | -1600 | -2100 | ^15,35^ |
| Tirumalai | Tamil Nadu | 12.866827 | 79.088723 | 1 | ass | Neolithic |  | -1700 | -1000 | -1350 | ^15^ |
| Togarappali | Tamil Nadu | 12.450551 | 78.348205 | 2 | ass | Neolithic |  | -1700 | -1000 | -1350 | ^15^ |
| Toranagallu | Karnataka | 15.19411 | 76.675003 | 3 | ass | Neolithic | Ashmound I/II | -2600 | -1600 | -2100 | ^8^ |
| Tsallakudluru | Andhra Pradesh | 15.81805556 | 77.53333333 | 3 | ass | Neolithic |  | -2200 | -1500 | -1850 | ^12^ |
| Tuljapur | Karnataka | 18.222905 | 77.430798 | 3 | ass | Neolithic |  | -2200 | -1200 | -1700 | ^15,30^ |
| Tuljapur Garhi | Maharashtra | 21.17523 | 77.59271 | 2 | C14 | Chalcolithic | Jorwe-like | -1500 | -1100 | -1300 | ^17^ |
| Ubhad | Gujarat | 21.52916667 | 74.32083333 | 3 | ass | Chalcolithic | Salvada | -2300 | -2000 | -2150 | ^57^ |
| Ubhad | Gujarat | 21.52916667 | 74.32083333 | 3 | ass | Early Historic |  | -350 | 300 | -50 | ^29^ |
| Ujjain | Madhya Pradesh | 23.17723 | 75.78845 | 1 | ass | Early Historic |  | -300 | 300 | 0 | ^31^ |
| Ujjini | Karnataka | 18.177643 | 77.54704 | 3 | ass | Neolithic |  | -2200 | -1200 | -1700 | ^15,30^ |
| Umraj | Karnataka | 17.33072658 | 75.6924481 | 1 | ass | Chalcolithic | Jorwe | -1500 | -1200 | -1350 | ^15,27^ |
| Unchahera | Madhya Pradesh | 23.404218 | 75.151977 | 2 | ass | Chalcolithic |  | -2450 | -1500 | -1975 | ^49^ |
| Untavad | Maharashtra | 21.321852 | 74.860663 | 3 | ass | Chalcolithic | Salvada | -2300 | -2000 | -2150 | ^28^ |
| Uparpinde | Maharashtra | 21.317899 | 74.802975 | 2 | ass | Chalcolithic | Salvada | -2300 | -2000 | -2150 | ^28,29^ |
| Uparpinde | Maharashtra | 21.317899 | 74.802975 | 2 | ass | Early Historic |  | -350 | 300 | -25 | ^28,29^ |
| Uppalapadu | Andhra Pradesh | 17.45334952 | 82.01436114 | 2 | ass | Neolithic |  | -1700 | -1000 | -1350 | ^9,15^ |
| Uravakonda | Andhra Pradesh | 14.98692853 | 77.24037964 | 1 | ass | Neolithic |  | -2200 | -1200 | -1700 | ^15,36^ |
| Urchan | Karnataka | 17.24348429 | 76.175007 | 1 | ass | Chalcolithic | Jorwe | -1500 | -1200 | -1350 | ^15,27^ |
| Utnur | Andhra Pradesh | 16.06045587 | 77.5953128 | 1 | C14 | Neolithic |  | -2900 | -2100 | -2500 | ^15^ |
| Uttawad | Madhya Pradesh | 22.073757 | 75.001903 | 3 | ass | Chalcolithic |  | -2450 | -1500 | -1975 | ^19,58^ |
| Uttawad | Madhya Pradesh | 22.073757 | 75.001903 | 3 | ass | Chalcolithic |  | -2000 | -1200 | -1600 | ^19,58^ |
| Uyyalawada | Andhra Pradesh | 15.1 | 78.4 | 2 | ass | Neolithic |  | -1700 | -1200 | -1450 | ^32^ |
| Vaderpura | Karnataka | 14.06666667 | 75.74166667 | 2 | ass | Neolithic |  | -2000 | -1200 | -1600 | ^11^ |
| Vadhode | Maharashtra | 21.23333333 | 74.93333333 | 2 | ass | Chalcolithic | Malwa | -1700 | -1500 | -1600 | ^29^ |
| Vadhode | Maharashtra | 21.23333333 | 74.93333333 | 2 | ass | Chalcolithic | Jorwe | -1500 | -1200 | -1350 | ^29^ |
| Vaindane | Maharashtra | 21.26666667 | 74.475 | 1 | ass | Chalcolithic | Jorwe | -1500 | -1200 | -1350 | ^27^ |
| Vaindane | Maharashtra | 21.26666667 | 74.475 | 1 | ass | Early Historic |  | -350 | 300 | -25 | ^27^ |
| Vajrakaruru | Andhra Pradesh | 15.08875361 | 77.35675117 | 1 | ass | Neolithic |  | -2200 | -1200 | -1700 | ^15,36^ |
| Vallampadu | Andhra Pradesh | 15.31666667 | 78.35 | 2 | ass | Neolithic |  | -1700 | -1200 | -1450 | ^32^ |
| Vanahalli | Karnataka | 15.523052 | 75.158908 | 2 | ass | Neolithic |  | -2500 | -1200 | -1850 | ^15^ |
| Varsus | Maharashtra | 21.29889 | 74.775613 | 2 | ass | Chalcolithic | Kayatha | -2450 | -2000 | -2225 | ^28^ |
| Veerapuram | Andhra Pradesh | 15.905668 | 78.248414 | 1 | C14 | Neolithic |  | -2200 | -1200 | -1700 | ^15^ |
| Vehergaon | Maharashtra | 21.10833333 | 74.4125 | 3 | ass | Chalcolithic | Jorwe | -1500 | -1200 | -1350 | ^29^ |
| Velagaturu | Andhra Pradesh | 15.25138889 | 78.29583333 | 3 | ass | Neolithic |  | -1700 | -1200 | -1450 | ^32^ |
| Velpumudugu | Andhra Pradesh | 15.050837 | 77.206956 | 3 | AMS | Neolithic | Ashmound IIIB | -1500 | -1300 | -1400 | ^15,53^ |
| Vemavaram | Andhra Pradesh | 16.594262 | 80.622715 | 2 | ass | Neo/CHalco |  | -1700 | -1000 | -1350 | ^21^ |
| Venkatapura | Karnataka | 15.3486392 | 75.856001 | 2 | ass | Neolithic |  | -2500 | -1200 | -1850 | ^15^ |
| Venkatapura | Karnataka | 13.093588 | 78.030804 | 2 | ass | Neolithic |  | -2000 | -1400 | -1700 | ^15^ |
| Venkatareddipalli | Andhra Pradesh | 14.41670804 | 77.25492608 | 1 | ass | Neolithic |  | -2200 | -1200 | -1700 | ^15^ |
| Vidupanakallu | Andhra Pradesh | 15.1411208 | 77.16764744 | 1 | ass | Neolithic |  | -2200 | -1200 | -1700 | ^15,36^ |
| Vitlampalli | Andhra Pradesh | 14.61744893 | 77.08909665 | 1 | ass | Neolithic |  | -2200 | -1200 | -1700 | ^15,36^ |
| Wadigiri | Karnataka | 16.81978009 | 76.52178546 | 1 | ass | Neolithic |  | -2500 | -1200 | -1850 | ^15^ |
| Wagzai-devi-deol | Maharashtra | 18.16666667 | 74.86666667 | 2 | ass | Early Historic |  | -350 | 300 | -25 | ^23^ |
| Walkhede | Maharashtra | 21.10972222 | 74.86666667 | 2 | ass | Chalcolithic | Salvada | -2300 | -2000 | -2150 | ^28^ |
| Walkhede | Maharashtra | 21.10972222 | 74.86666667 | 2 | ass | Chalcolithic | Late Harappan | -2000 | -1700 | -1850 | ^28^ |
| Walki | Maharashtra | 18.59001 | 74.22546 | 2 | C14 | Chalcolithic | Late Jorwe | -1200 | -800 | -1000 | ^22^ |
| Wandalli | Karnataka | 16.28156177 | 76.80980499 | 1 | ass | Neolithic | Ashmound I/II | -2600 | -1600 | -2100 | ^15,35^ |
| Wangdari I | Maharashtra | 18.604609 | 74.552738 | 3 | ass | Early Historic |  | -350 | 300 | -25 | ^23^ |
| Wanmarapalli | Karnataka | 18.303323 | 77.426964 | 3 | ass | Neolithic |  | -2200 | -1200 | -1700 | ^15,30^ |
| Wardhamanukota | Andhra Pradesh | 17.133412 | 79.642005 | 2 | ass | Neolithic |  | -2200 | -1200 | -1700 | ^21^ |
| Wardhan | Maharashtra | 21.34791419 | 72.8420406 | 1 | ass | Chalcolithic | Jorwe | -1500 | -1200 | -1350 | ^27^ |
| Watgal | Karnataka | 16.100153 | 76.750473 | 3 | C14 | 2800 BC-1000 BC |  | -2800 | -1000 | -1900 | ^15,53^ |
| Yatakal | Andhra Pradesh | 14.55635387 | 77.09782452 | 1 | ass | Neolithic |  | -2200 | -1200 | -1700 | ^15,36^ |
| Yavagal | Karnataka | 15.697675 | 75.519147 | 2 | ass | Neolithic |  | -2600 | -1800 | -2200 | ^15^ |
| Yellavaram | Andhra Pradesh | 17.49516742 | 82.03777917 | 2 | ass | Neolithic |  | -1700 | -1000 | -1350 | ^9,15^ |
| Yengunda | Karnataka | 18.239677 | 77.483648 | 3 | ass | Neolithic |  | -2200 | -1200 | -1700 | ^15,30^ |
| Yenkura | Karnataka | 17.93163 | 77.185985 | 3 | ass | Neolithic |  | -2200 | -1200 | -1700 | ^15,30^ |
| Yergatti | Karnataka | 15.93244719 | 75.06714136 | 1 | ass | Neolithic |  | -2200 | -1200 | -1700 | ^35^ |
| Yergunti | Karnataka | 16.24083174 | 76.49269258 | 1 | ass | Neolithic | Ashmound I/II | -2600 | -1600 | -2100 | ^35^ |
| Yesar | Maharashtra | 20.98333333 | 74.325 | 2 | ass | Chalcolithic | Jorwe | -1500 | -1200 | -1350 | ^29^ |
| Yesar | Maharashtra | 20.98333333 | 74.325 | 2 | ass | Early Historic |  | -350 | 300 | -25 | ^29^ |
| Yetakallu | Andhra Pradesh | 14.518718 | 77.068269 | 2 | ass | Neolithic |  | -2200 | -1200 | -1700 | ^15^ |
| Zoo area | Andhra Pradesh | 17.73603857 | 83.2638802 | 2 | ass | Neolithic |  | -1700 | -1000 | -1350 | ^9,15^ |

**REFERENCES:**

1. Prasad, S. *et al.* Prolonged monsoon droughts and links to Indo-Pacific warm pool: A Holocene record from Lonar Lake, central India. *Earth Planet. Sci. Lett.* **391**, 171–182 (2014).

2. Anoop, A. *et al.* Palaeoenvironmental implications of evaporative gaylussite crystals from Lonar Lake, central India. *J. Quat. Sci.* **28**, 349–359 (2013).

3. Gaussen, H., et al. *International Map of the Vegetation at Scale 1:1.000.000*. (French Institute of Pondycherry, 1964).

4. McDermott, F. Palaeo-climate reconstruction from stable isotope variations in speleothems: a review. *Quat. Sci. Rev.* **23**, 901–918 (2004).

5. Baldini, J., McDermott, F. & Fairchild, I. Spatial variability in cave drip water hydrochemistry: Implications for stalagmite paleoclimate records. *Chem. Geol.* **235**, 390–404 (2006).

6. Angadi, S. S. Archaeology of the Tungabhadra Valley with special reference to Haveri district. (Karnatak University, 2012).

7. Bauer, A. M. Socializing environments and ecologizing politics: Social differentiation and the production of nature in Iron Age northern Karnataka. (University of Chicago, 2010).

8. Boivin, N. Landscape and Cosmology in the South Indian Neolithic: New Perspectives on the Deccan Ashmounds. *Camb. Archaeol. J.* **14**, 235–257 (2004).

9. David Raju, B. The Settlement and Subsistence Pattern of Neolithic Culture of Lower Krishna Valley. in *Proceedings of the Indian History Congress* **49**, 629–639 (1988).

10. David Raju, B. Settlement and subsistence pattern of the Neolithic Culture of the Lower Krishna Valley. *Man Environ.* **15**, 45–51 (1990).

11. Poonancha. Archaeology of Malnad Region. (Karnatak University, 1990).

12. Venkatasubbaiah, P. C. A Preliminary study on the Ashmound sites in the Lower Tungabhadra Region of Andhra Pradesh. *Anc. Asia* **3**, 36–38

13. Jha, V. D. The Archaeology of Baster Region. (University of Saugar, 1980).

14. Johansen, P. G. Early ironworking in Iron Age South India: new evidence for the social organization of production from northern Karnataka. *J. Field Archaeol.* **39**, 256–275 (2014).

15. Korisettar, R., Venkatasubbaiah, P. C. & Fuller, D. Q. Appendix to: Brahmagiri and Beyond: the Archaeology of the Southern Neolithic. in *Indian Archaeology in Retrospect* (eds. Settar, S. & Korisettar, R.) **Volume I. Prehistory**, 436–478 (Manohar, 2001).

16. Kajale, M. D. On the occurrence of ancient agricultural patterns during the Chalcolithic periods (c. 1600–1000 BC) at Apegaon, District Aurangabad in central Godavari valley, Maharashtra. in *Apegaon Excavations (1976)* (eds. Deo, S. B., Dhavalikar, M. K. & Ansari, Z. D.) 50–56 (1979).

17. Kajale, M. D. Ancient plant economy at Chalcolithic Tuljapur Garhi. District Amraoti, Maharashtra. *Curr. Sci.* **57**, 377–379 (1988).

18. Kajale, M. D. Archaeobotanical investigations on a multicultural site at Adam, Maharashtra, with special reference to the development of tropical agriculture in arts of India. in *Tropical archaeobotany: Applications and new developments* (ed. Hather, J.) 34–50 (London, 1994).

19. Mishra, S., Ota, S. B., Shete, G., Naik, S. & Deotare, B. C. Late Quaternary Alluvial History and Archaeological sites in the Nimar Region of Western Madhya Pradesh, India. *Man Environ.* **24**, 149–157 (1999).

20. Morrison, K. *Daroji Valley: Landscape History, Place, and the Making of a Dryland Reservoir System*. (Manohar, 2009).

21. Murty, M. L. K. *Pre- and Protohistoric Andhra Pradesh up to 500 BC*. (Orient Longman, 2003).

22. Naik, S. & Mishra, S. The Chalcolithic Phase in the Bhima Basin, Maharashtra: a Review. *Man Environ.* **22**, 45–58 (1997).

23. Panja, S. Mobility strategies, site structure and subsistence-settlement organization: a case study of the Chalcolithic in the middle Bhima valley with special reference to Inamgaon. (University of Poona, 1995).

24. Rajan, K. Archaeology of Dharmapuri District, Tamil Nadu. *Man Environ.* (1991).

25. Rajan, K. *Archaeology of Palat Basin*. (Tamil University, 2000).

26. Roberts, P. *et al.* Local diversity in settlement, demography and subsistence across the southern Indian Neolithic-Iron Age transition: site growth and abandonment at Sanganakallu-Kupgal. *Archaeol. Anthropol. Sci.* **8**, 575–599 (2016).

27. Shinde, V. S. New light on the origin, settlement system and decline of the Jorwe culture in the Deccan India. *South Asian Stud.* **5**, 59–72 (1989).

28. Shinde, V. S. Settlement pattern of the Savalda culture—the first farming community of Maharashtra. *Bull. Deccan Coll. Res. Inst.* **49-50**, 417–426 (1990).

29. Shinde, V. S. *Early Settlements in the Central Tapi Basin*. (Munshiram Manoharlal, 1998).

30. Shadaksharaiah, R. M. Archaeology of Bidar District with Special Reference to the Manjra and Karanja Valleys. (Karnatak University, 1995).

31. Sharma, R. K. & Misra, O. P. *Archaeological Excavations in Central India (Madhya Pradesh and Chhattisgarh)*. (Mittal Publications, 2003).

32. Venkatasubbaiah, P. C. Recent archaeological investigations in the Kurnool District, Andhra Pradesh. in *Proceedings of the Andhra Pradesh History Congress* **22**, 8–12 (1998).

33. Venkatasubbaiah, P. C. & Kajale, M. D. Biological remains from Neolithic and Early Historic Sites in Cuddapah District, Andhra Pradesh. *Man Environ.* **16**, 85–97 (1991).

34. Thapar, B. K. *Indian Archaeology 1974-75 - a review*. (Archaeological Survey of India, 1979).

35. Paddayya, K. *Investigations into the neolithic culture of the Shorapur Doab, South India*. **3**, (Brill, 1973).

36. Rami Reddy, V. *The Prehistoric and Protohistoric Cultures of Southwestern Andhra Pradesh*. (Government of Andhra Pradesh, 1976).

37. Archaeological Survey of India. *IAR Report 1959-60*. (Government of India).

38. Archaeological Survey of India. *IAR Report 1966-67*. (Government of India).

39. Archaeological Survey of India. *IAR Report 1982-83*. (Government of India).

40. Archaeological Survey of India. *IAR Report 1965-66*. (Government of India).

41. Archaeological Survey of India. *IAR Report 1958-59*. (Government of India).

42. Archaeological Survey of India. *IAR Report 1964-65*. (Government of India).

43. Archaeological Survey of India. *IAR Report 1984-85*. (Archaeological Survey of India).

44. Archaeological Survey of India. *IAR Report 1968-69*. (Government of India).

45. Archaeological Survey of India. *IAR Report 1983-84*. (Government of India).

46. Subbarao, B. Archaeological explorations in Bellary. *Bull. Deccan Coll. Res. Inst.* **8**, 209–224 (1947).

47. Nagaraja Rao, M. S. *Indian Archaeology 1983-84 - a review*. (Archaeological Survey of India, 1986).

48. Babu Rajeev, C. *Indian Archaeology 2000-01 - a review*. (Archaeological Survey of India, 2006).

49. Tripathi, R. C. *Indian Archaeology 1984-85 - a review*. (Archaeological Survey of India, 1987).

50. Ghosh, A. *Indian Archaeology 1964-65 - a review*. (Archaeological Survey of India, 1969).

51. Krishna Sastry, V. V. *Proto and Early Historical cultures of Andhra Pradesh*. (Government of Andhra Pradesh, 1983).

52. Fuller, D. Q. Agricultural Origins and Frontiers in South Asia: A Working Synthesis. *J. World Prehistory* **20**, 1–86 (2006).

53. Fuller, D. Q., Boivin, N. & Korisettar, R. Dating the Neolithic of South India: new radiometric evidence for key economic, social and ritual transformations. *Antiquity* **81**, 755–778 (2007).

54. Vishnu-Mittre. *Plant economy in ancient Navdatoli. Technical reports on archaeological remains*. **2**, (Deccan College Postgraduate and Research Institute, 1961).

55. Debala-Mittra. *Indian Archaeology 1980-81 - a review*. (Archaeological Survey of India, 1983).

56. Dhavalikar, M. K. Chalcolithic burials: the Tekwada evidence. *Pratattva* **2**, 35–40 (1968).

57. Pappu, R. S. & Shinde, V. Site Catchment analysis of the Deccan Chalcolithic Cultures in the Central Tapi basin. *Bull. Deccan Coll. Post-Grad. Res. Inst.* 317–338 (1990).

58. Ota, S. B. & Sahu, P. Palas leaf impression on burial pot from Chalcolithic Utawad, Madhya Pradesh. *Man Environ.* **31**, (2006).
